# Computational and NMR spectroscopy insights into the conformation of cyclic di-nucleotides

**DOI:** 10.1038/s41598-017-16794-4

**Published:** 2017-11-29

**Authors:** Baifan Wang, Zhenghua Wang, Uroš Javornik, Zhen Xi, Janez Plavec

**Affiliations:** 1 Slovenian NMR Center, National Institute of Chemistry, Hajdrihova 19, Ljubljana, Slovenia; 2 State Key Laboratory of Elemento-Organic Chemistry and Department of Chemical Biology, Nankai University, Collaborative Innovation Center of Chemical Science and Engineering, Tianjin, 300071 P. R. China; 3 EN-FIST Center of Excellence, Trg OF 13, 1000 Ljubljana, Slovenia; 4 Faculty of Chemistry and Chemical Technology, University of Ljubljana, Večna pot 113, Ljubljana, Slovenia

## Abstract

Cyclic di-nucleotides (CDNs) are second messengers in bacteria and metazoan that are as such controlling important biological processes. Here the conformational space of CDNs was explored systematically by a combination of extensive conformational search and DFT calculations as well as NMR methods. We found that CDNs adopt pre-organized conformations in solution in which the ribose conformations are North type and glycosidic bond conformations are *anti* type. The overall flexibility of CDNs as well as the backbone torsion angles depend on the cyclization of the phosphodiester bond. Compared to di-nucleotides, CDNs display high rigidity in the macrocyclic moieties. Structural comparison studies demonstrate that the pre-organized conformations of CDNs highly resemble the biologically active conformations. These findings provide information for the design of small molecules to modulate CDNs signalling pathways in bacteria or as vaccine adjuvants. The rigidity of the backbone of CDNs enables the design of high order structures such as molecular cages based on CDNs analogues.

## Introduction

**Figure 1:**
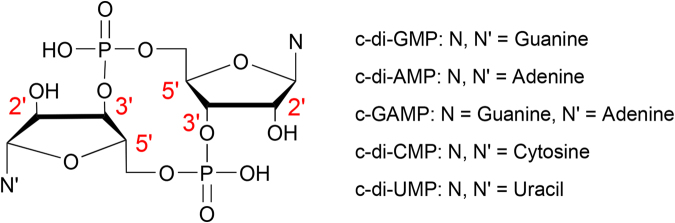
Chemical structures of cyclic di-nucleotides.

Cyclic di-nucleotides (CDNs) are composed of two nucleosides joined by two phosphate groups in a macrocycle (Fig. 1). CDNs have emerged as important second messengers in mammalian and bacteria cells^1,2^. In mammalian cells, cyclic GMP-AMP whose phosphate groups connect the two nucleosides from the 2′- and 5′- positions of guanosine and the 3′- and 5′- positions of adenosine (denoted as 2′3′-cGAMP), serves as a second messenger in the cell signalling pathway. 2′3′-cGAMP triggers the innate immune system by activating the adaptor protein stimulator of IFN genes (STING), which links the upstream cytosolic DNA detection and the downstream cytokine production^3,4^. In bacteria, bis-(3′–5′)-cyclic dimeric guanosine monophosphate (c-di-GMP) regulates a variety of processes such as cell motility, intercellular interactions, biofilm formation, dispersal and responses to oxidation^1,2,5,6^. Bis-(3′–5′)-cyclic dimeric adenosine monophosphate (c-di-AMP) was identified as a crucial second messenger in the regulation of cell size, envelope stress control, fatty acid synthesis, ion transport and metabolite balance^7–10^. Bis-(3′–5′)-cyclic guanosine monophosphate-adenosine monophosphate (c-GAMP) has been implicated in affecting bacteria intestinal colonization^11^. Modulating CDN signalling pathways in bacteria could represent a new way of controlling life processes in medical and industrial settings. CDNs are recognized also by mammalian immune systems as a uniquely bacterial molecule and therefore are considered promising vaccine adjuvants^1,2^.

There is a significant amount of data on the structural biology of CDN receptors. For example, crystal structures of c-di-GMP bound to various protein receptors which contain GGDEF I site, EAL, PilZ, BldD and VpsT domains as well as STING have been reported^1,12^. In the bound states c-di-GMP can adopt conformations ranging from a stacked form to a more extended form^12^. Moreover, c-di-GMP can bind to proteins in monomeric, dimeric or even tetrameric form^13^. For c-di-AMP, the structures of its complex with tricarboxylic acid cycle enzyme pyruvate carboxylase^10^, CTD domain^14,15^, BsDisA diadenylate cyclase^16^, phosphodiesterase PgpH HD domain^17^ and PstA^18–21^ have been determined. For c-GAMP, currently only two crystal structures of the molecule bound to mammalian STING are available^22^. Besides the protein receptors, CDNs have been shown to bind to riboswitches^23–25^. However, the conformation and dynamics of CDNs were rarely studied experimentally in solution.

Despite the increasing interest in using CDNs to study signalling pathways in cells or as therapeutic agents, the current information on CDNs physicochemical properties is insufficient. It has been shown that the conformation of CDNs in solution is crucial for the evaluation of the binding constants to their receptors^26^. Such data are helpful for the understanding of the structure-activity relationship of either CDNs or their analogues and the design of potent therapeutic agents based on CDNs scaffold. The absence of comprehensive understanding on the conformation of free CDNs prompted us to perform thorough computational and NMR studies of the effect of nucleobase and cyclization through the phosphate linker. Specifically, we studied the conformation of CDNs composed of four major types of nucleobases (Fig. 1) by replica exchange molecular dynamics (REMD) simulations in implicit and explicit water environment and DFT calculation. To supplement the computational results, we synthesised five CDNs and analyzed their conformations in aqueous solution by NMR. The computational and NMR analysis provide parameters such as ribose puckering, conformational preference across glycosidic bond and backbone torsion angles.

## Results and Discussion

### Computational study of CDNs

**Figure 2:**
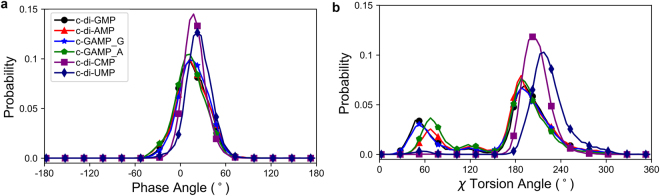
The probability distribution of phase angle of pseudorotation (**a**) and χ torsion angle (**b**) for CDNs. Data were obtained from REMD simulations at 300 K. The same colour scheme and style are used in both graphs.

Firstly Replica exchange molecular dynamics (REMD)^27^ simulations were performed on CDNs with 24 temperature states ranging from 273.0 to 583.5 K with simulation time up to 60 ns in implicit solvent. For temperature state at 300 K, a total of 30 000 conformations of CDNs were sampled. REMD was proved to effectively search the conformational space of CDNs as reflected in the transformation of χ torsion angle from *anti* to *syn* and conversions between North (N-) and South (S-) type ribose conformations (see Supplementary Fig. S1). At 300 K the phase angles of pseudorotation^28,29^ for all five CDNs are in the range from −60° to 60° corresponding to the N-type conformation (Fig. 2a).

**Figure 3:**
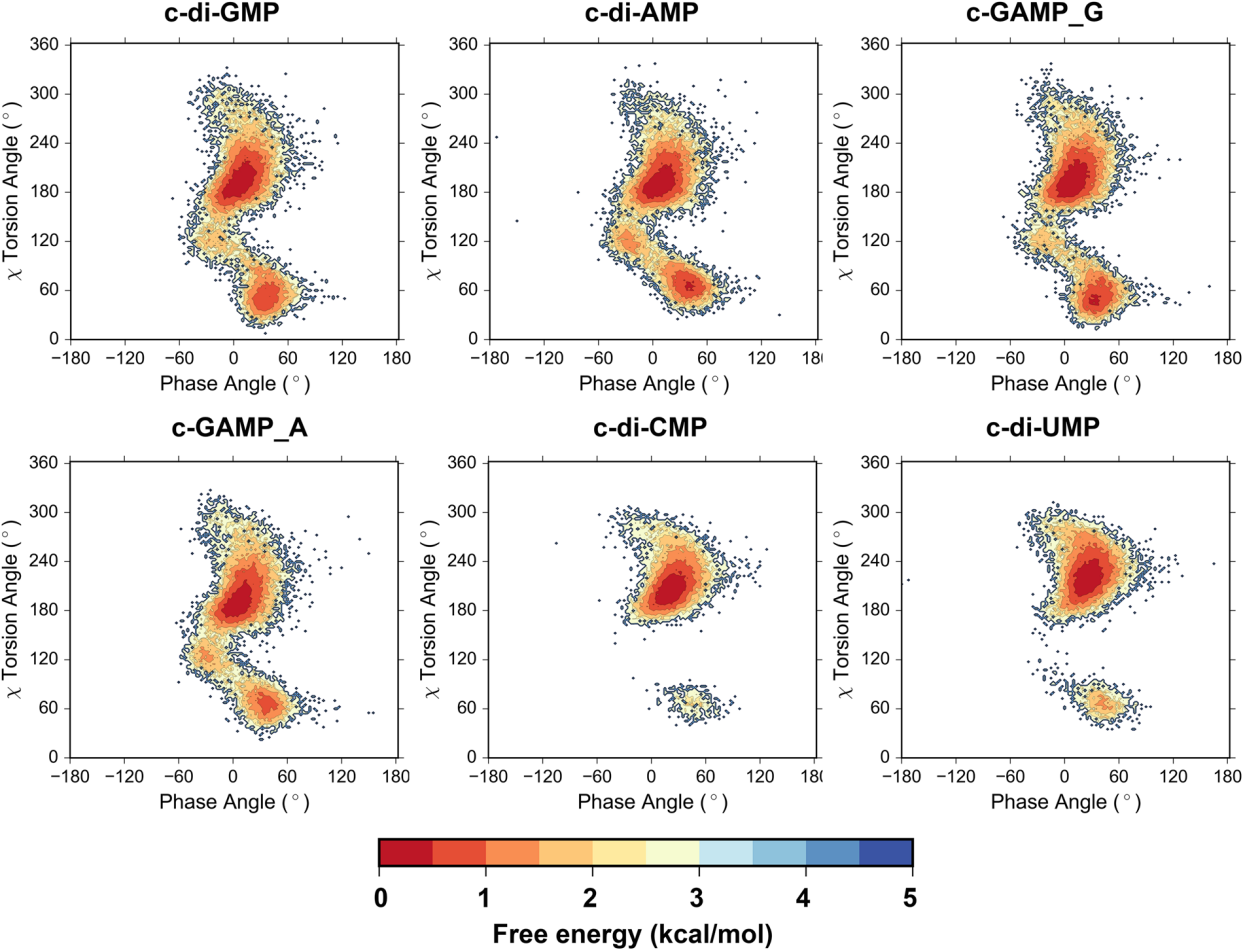
Population based free energy plot on χ-phase angles plane for the CDNs based on REMD simulations at 300 K. Free energy estimates were calculated using the following equation: G_i_ = −k_B_T*ln(N_i_/N_max_), where k_B_ is Boltzmann constant, T is temperature, N_i_ is the population of bin i and N_max_ is the population of the most populated bin.

As for the conformation across glycosidic bonds, CDNs which are composed of pyrimidine bases mostly adopt *anti* conformation, while their counterparts with purines in addition display minor, but perceptible population of *syn* conformers (Fig. 2b). It is interesting to note that guanine and adenine moieties display slight differences in populations of *syn* conformers. As a consequence, free energy landscapes for χ torsion angles and pseudorotational equilibria of sugar moieties show that CDNs composed of purine bases possess two stable states, of which the glycosidic bond in the *anti* region exhibits lower energy (about 1 kcal/mol) compared to the *syn* region; while the CDNs composed of pyrimidine bases exhibit a single state (Fig. 3).

**Figure 4:**
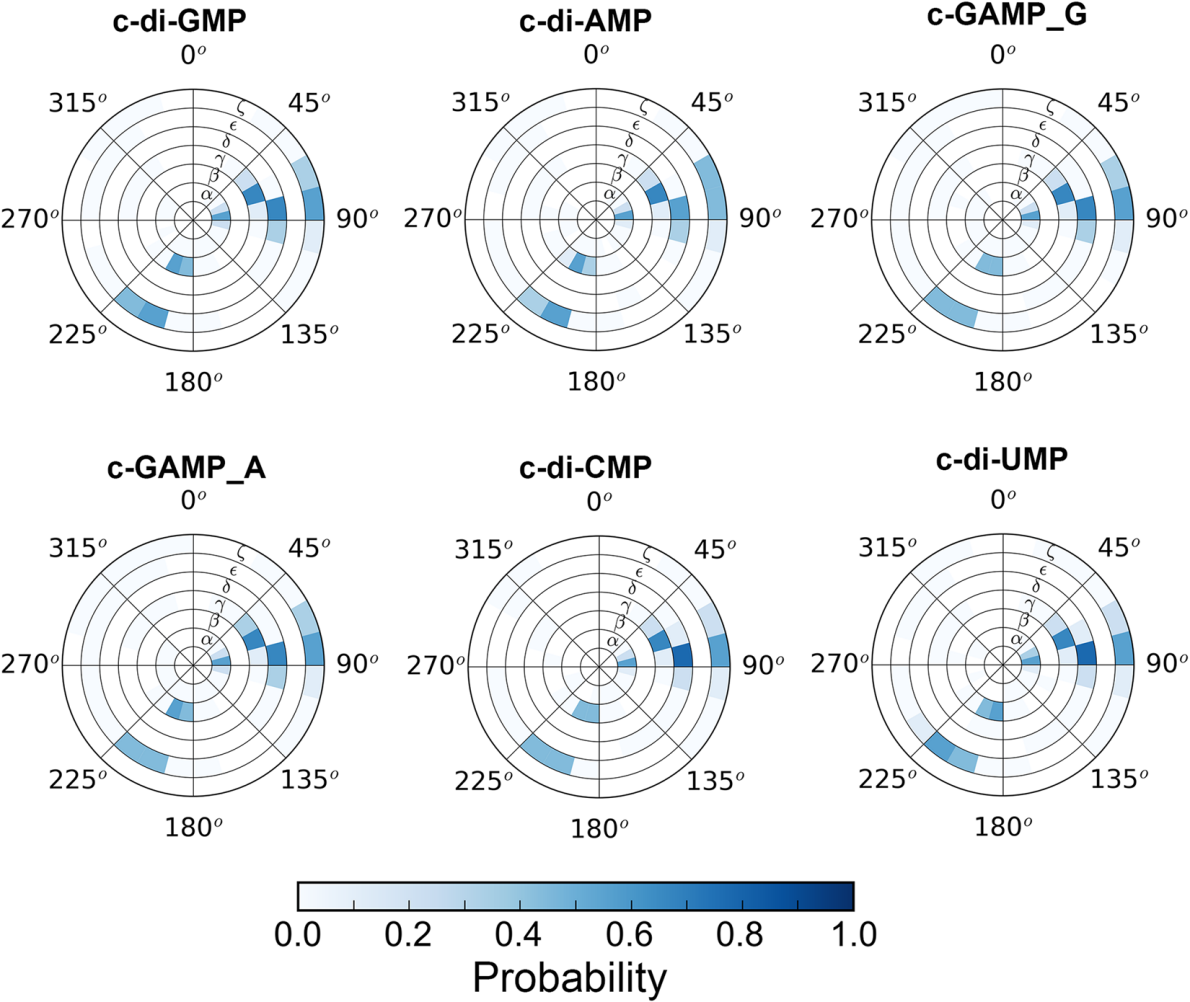
Conformational wheels for CDNs from REMD simulations in implicit solvent at 300 K.

The influence of cyclization of the phosphodiester groups on CDNs backbone torsion angles was analyzed by REMD simulations. The distribution of the six backbone torsion angles α, β, γ, δ, ε and ζ for individual CDN is shown in Fig. 4. Compared to the allowed ranges of backbone torsion angles in nucleosides, nucleotides, oligo- and poly-nucleotides^30^, it can be seen that torsion angles of CDNs show very narrow distributions. The dominant conformations of α (*g* ^+^), β (*t*), γ (*g* ^+^), δ (*g* ^+^), ε (*t*) and ζ (*g* ^+^) torsion angles are in accord with spatial restraints imposed by a 12-member macrocyclic moiety.

To evaluate the effect of solvent on the conformational space of CDNs, REMD simulations were also performed in explicit water solvent. In the presence of explicit water molecules phase angles of pseudorotation are N-type for all five CDNs (see Supplementary Fig. S2). Noteworthy, similar observations can be made based on implicit solvent simulations. For the conformation across glycosidic bonds, the population of *syn* conformers of the guanine and uracil moieties (50% for guanine and 25% for uracil moiety) were significantly higher compared to REMD simulations in implicit solvent. CDNs composed of adenine and cytosine moieties displayed similar populations as in implicit solvent simulations. Examination of the CDN conformations showed that the amino group of guanine base forms a hydrogen bond with the phosphate group when the guanine moiety is in *syn* glycosidic conformation (see Supplementary Fig. S3). Comparison of the backbone torsion angles of CDNs obtained by simulations in implicit and explicit solvent showed no significant differences (see Supplementary Fig. S4).

### NMR study of CDNs

All five CDNs examined by REMD simulations were synthesized (see Supplementary text) and subjected to NMR study. NMR spectra were measured at five temperatures ranging from 273 to 353 K to investigate the influence of temperature on the conformation of CDNs. The coupling constants between sugar protons, carbon and phosphorus (J_H-H_, J_H-P_ and J_C-P_) were determined for CDNs by various NMR spectra (see Supplementary Fig. S5–S9). The analysis of the ^3^J_H-H_ coupling constants (Supplementary Table S1) defining the sugar pucker and N-S populations showed that for c-di-CMP, c-di-UMP and c-di-GMP, the N-type ribose conformers dominate at 298 K (Table 1). The ^3^J_H-H_ coupling constants for the guanine moiety of c-GAMP cannot be determined at 298 K, but at 333 K the population of N-type conformers is about 95% (Table 1 and Supplementary Table S1). At 333 and 353 K, there is a slight tendency for CDNs composed of purine moieties to exhibit minor populations of S-type conformations, while CDNs composed of pyrimidines display 100% of N-type conformation (Supplementary Table S1).

**Table 1:** The predominant glycosidic bond and ribose conformations of CDNs calculated from NMR measurements and DFT calculations. ^1^Values in parenthesis are from DFT calculation. ^2^The value of phase angle of pseudorotation for ribose of guanine moiety of c-GAMP was determined at 333 K.

CDNs	χ [type]	P_N_ [deg]	ψ_N_ [deg]	ribose conformer
c-di-GMP	*anti* (211)^1^	0 (15)	41 (37)	100% N-type (N-type)
c-di-AMP	*anti* (210)	4 (13)	42 (35)	100% N-type (N-type)
c-GAMP (guanine moiety)	*anti* (210)	4 (13)	40 (34)	95% N-type^2^ (N-type)
c-GAMP (adenine moiety)	*anti* (212)	1 (13)	40 (34)	100% N-type (N-type)
c-di-CMP	*anti* (201)	19 (12)	43 (37)	100% N-type (N-type)
c-di-UMP	*anti* (204)	18 (12)	40 (36)	100% N-type (N-type)

The conformation of χ torsion angles of the CDNs in solution was estimated from NOESY and ROESY spectra (see Supplementary Fig. S10–S14). The relative intensities of cross-peaks between aromatic H6 or H8 protons and ribose ring protons indicate a general preference for *anti* orientation of χ torsion angle. The presence of H2-H2′ and H2-H3′ correlations for adenine moieties, which are not possible for *anti* orientation of χ, indicates that a fraction of the population takes up *syn* conformation.

A series of transient 1D NOESY experiments was conducted for all five CDNs to obtain further semi-quantitative information about the conformational preferences of χ torsion angles. The results show a general preference for *anti* conformation for all CDNs, reflected by higher intensities of H8/H6 to H2′/H3′ relative to H8/H6 to H1′ NOE enhancements. The preference is greater for pyrimidine analogues, where η(H6-H1′) is undetectable for c-di-CMP. η(H6-H1′) enhancement is approximately 8 times lower than η(H6-H2′/H3′) for c-di-UMP. The ratio of η(H8-H1′) to η(H8-H2′/H3′) is approximately 1:2 for c-di-GMP and 1:4 for c-di-AMP. This indicates that significant populations of χ-*syn* conformers exist for these CDNs. The data also suggests that the prevalence of *syn* conformation of χ torsion angle is higher in c-di-GMP than c-di-AMP (Supplementary Table S2).

The results of REMD simulations in implicit solvent indicated significant population of molecules with *syn* conformation of χ, but did not indicate any difference in populations between c-di-GMP and c-di-AMP. On the other hand, REMD simulations with explicit solvent display higher fraction of *syn* conformers for guanine in comparison to adenine moiety. However, the fraction of *syn* conformers of χ for guanine moiety (about 50%) was overestimated according to NMR results. We suggest that formation of a hydrogen bond between the guanine amino and phosphate groups plays a role in the conformational equilibrium of χ conformers. Explicit water molecules can stabilize this bonding, but with the applied force field, this effect might have been exaggerated.

The populations of *g* ^+^, *g* ^*−*^ and *t* conformers across C4′-C5′ bond were determined through the analysis of ^3^J_H4′-H5′_ and ^3^J_H4′-H5″_ coupling constants (Supplementary Table S3). At 298 K, all CDNs adopt 100% *g* ^+^ conformer population along γ torsion angle. The ^3^J_H4′-H5′_ and ^3^J_H4′-H5″_ coupling constants for c-GAMP could not be determined. The β and ε torsion angles which describe the rotation of O5′-C5′ and C3′-O3′ bonds were calculated based on ^3^J_H-P_, ^4^J_H-P_ and ^3^J_C-P_ coupling constants (Supplementary Table S3) using the parametrization of Karplus equation for CCOP fragments^31,32^. The β torsion angles for all CDNs are in the range from −170° to −160°, while the ε torsion angles are in the range from −160° to −150°.

**Figure 5:**
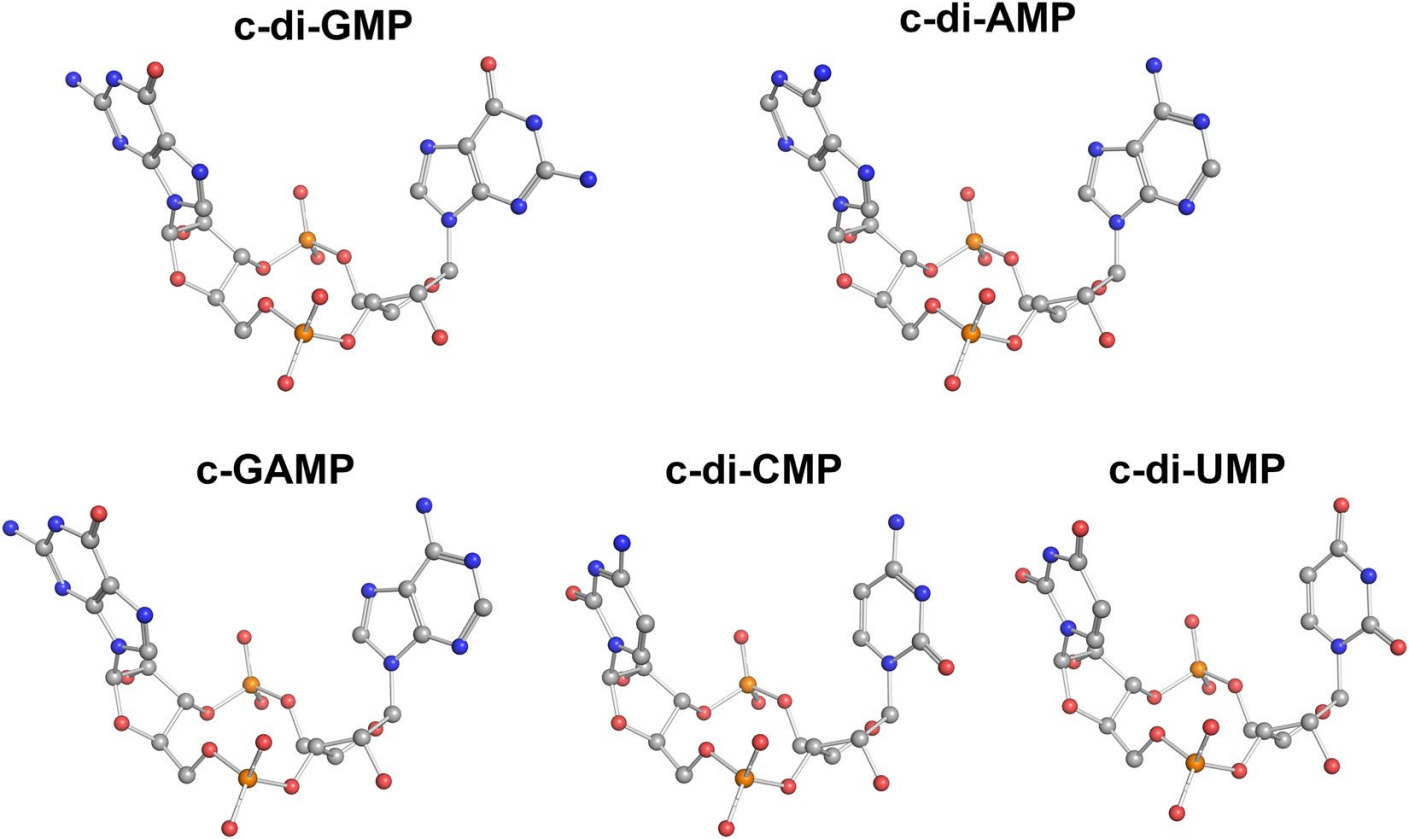
Lowest energy structures of CDNs optimized at B3LYP/6–31 G(d,p) level.

Since the ribose and backbone conformations of CDNs predicted by both implicit and explicit REMD simulations are highly similar, and considering that the predominant glycosidic bond population observed by NMR is *anti*, we chose the lowest energy conformation observed on the free energy landscape of CDNs from implicit REMD simulation (Fig. 3) for further analysis. DFT calculations were performed on these structures. The comparison of DFT optimized structures shows that despite different orientations of nucleobases in CDNs, conformations of their macrocyclic moieties highly resemble each other (Fig. 5). The structural parameters computed from DFT optimized CDNs show nice agreement with NMR measurements (Tables 1 and 2).

**Table 2:** Backbone parameters of CDNs calculated from NMR measurements and DFT calculations. ^1^Values in parenthesis are from DFT calculation. n.d.: not determined.

CDNs	α [deg]	β [deg]	γ [type]	δ [deg]	ε [deg]	ζ [deg]
c-di-GMP	n.d. (75)^1^	−170 (−173)	g^+^ (g^+^)	86 (84)	−150 (−155)	n.d. (69)
c-di-AMP	n.d. (70)	−170 (−174)	g^+^ (g^+^)	84 (87)	−150 (−154)	n.d. (70)
c-GAMP (guanine moiety)	n.d. (71)	−160 (−174)	n.d. (g^+^)	86 (87)	−150 (−155)	n.d. (69)
c-GAMP (adenine moiety)	n.d. (71)	−170 (−174)	g^+^ (g^+^)	87 (88)	−160 (−155)	n.d. (69)
c-di-CMP	n.d. (73)	−170 (−170)	g^+^ (g^+^)	79 (81)	−150 (−161)	n.d. (73)
c-di-UMP	n.d. (76)	−170 (−174)	g^+^ (g^+^)	82 (81)	−150 (−158)	n.d. (71)

### Comparison of the conformation between CDNs and linear di-nucleotides

**Figure 6:**
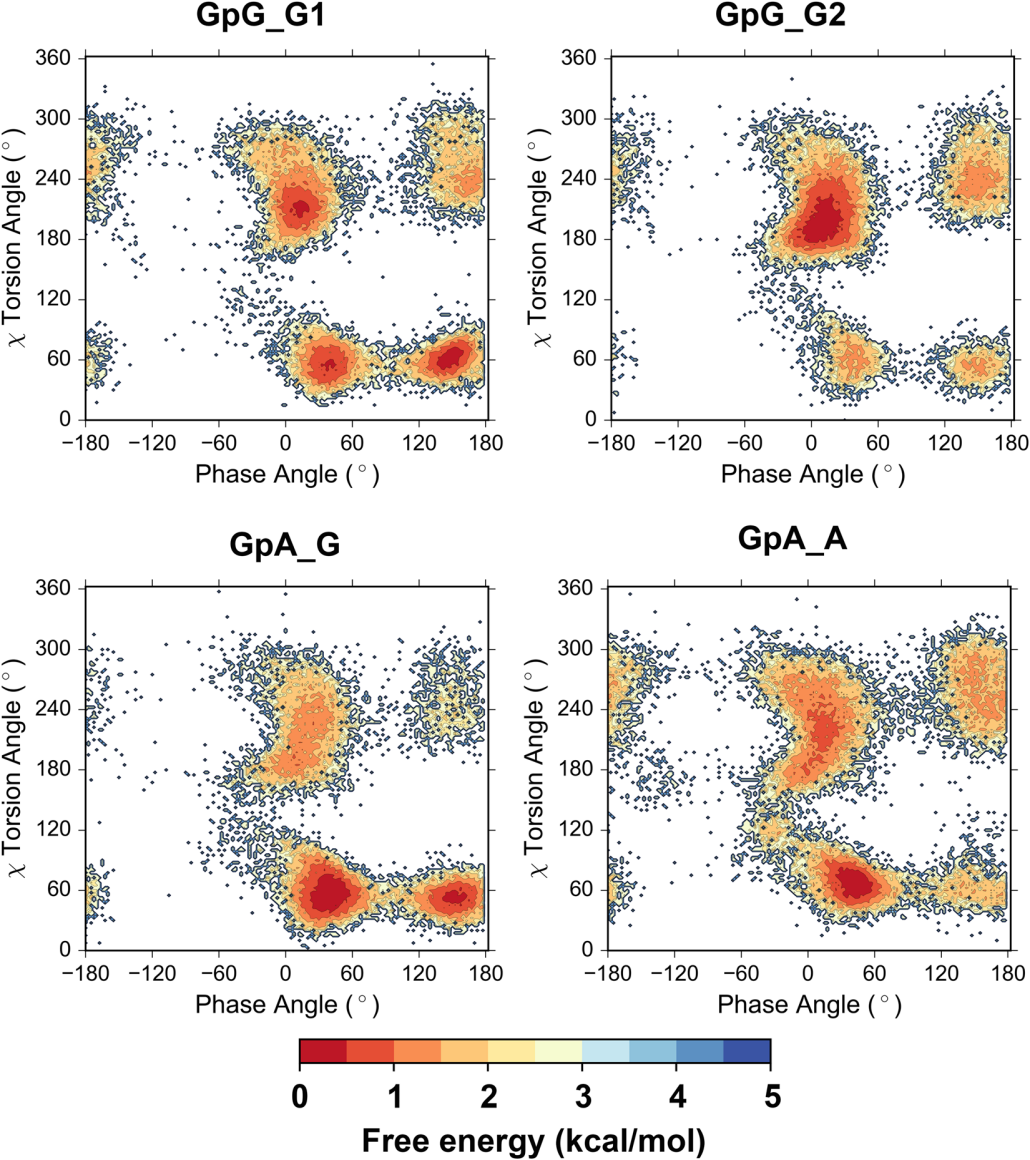
Population based free energy plot on the χ-phase angles plane for di-nucleotides from REMD simulation at 300 K.

Earlier NMR studies have demonstrated that di-nucleotides, ApA, ApC and CpG adopt both S- and N-type ribose conformations^33–35^. REMD simulations showed that GpG and GpA adopt both S- and N-type ribose as well as *syn* and *anti* conformations of χ torsion angle (Supplementary Fig. S15). As a consequence, four conformation states were observed for each moiety of di-nucleotides (Fig. 6). The backbone torsion angles of di-nucleotides were found to have much wider distributions compared to CDNs (Supplementary Fig. S16).

For the di-nucleoside polyphosphates, the ribose moieties showed a small preference for S-type conformation, but when attached to cytosine the ribose ring preferred N-type conformation^36^. The conformation around the glycosidic bond in di-nucleoside polyphosphates was found to be *anti*/*high anti* ^36^. The conformations of di-nucleoside polyphosphates were found to be highly dynamic, hence no single predominant conformation was observed.

In comparison, CDNs display mainly N-type ribose conformation and *anti* glycosidic bond conformation. Shifting the ribose conformation from N- to S-type results in changing the O3′-C3′ bond orientation from pseudo-equatorial (*e*) to pseudo-axial (*a*) position (Supplementary Fig. S17) which might be accompanied by rotation of the phosphate group. The phosphate group attached at the O3′ is relatively free to rotate in di-nucleotides, while rotation is restricted in the CDNs due to the macrocycle. This effect may be the origin of the different conformational preferences for CDNs and linear di-nucleotides.

### Structural comparison of CDNs in solution and bound state

**Figure 7:**
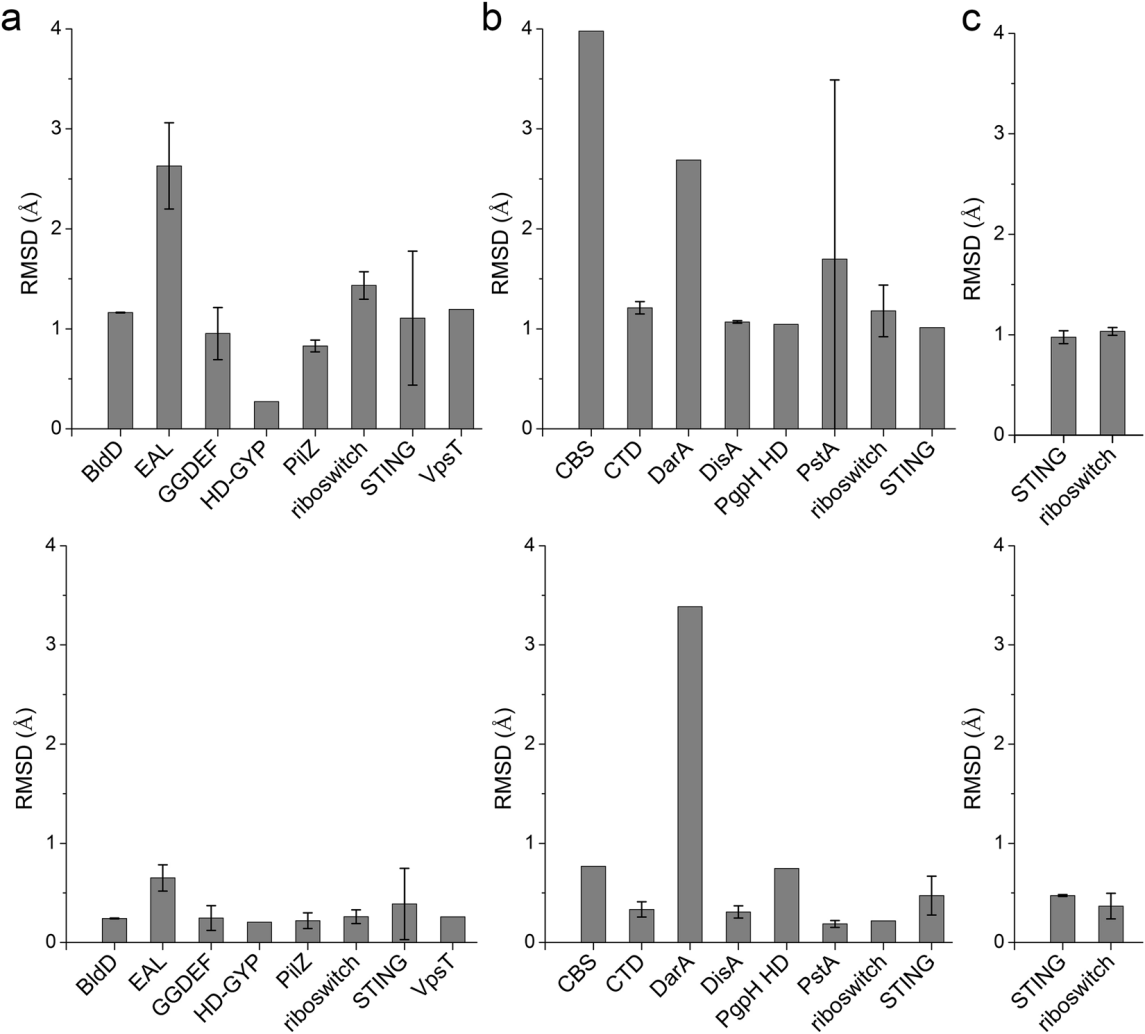
Comparison of the overall (upper panel) and backbone (macrocyclic moieties, lower panel) structural similarity expressed in terms of RMSD between various receptor-bound and DFT optimized CDN conformations. (**a**): c-di-GMP; (**b**): c-di-AMP, (**c**): c-GAMP.

The coordinates of CDNs bound to receptors were extracted and compared to the solution conformations. Except for the c-di-GMP bound to EAL domain based proteins, the superimpositions of the DFT optimized conformation and the receptor-bound conformations show overall and backbone (macrocyclic moieties) RMSD values less than 2.0 and 1.0 Å, respectively (Fig. 7a and Supplementary Fig. S18). Our DFT-optimized c-di-GMP conformation showed striking similarity in the conformation of macrocyclic moieties to the bound states for most type of receptors. RMSD values for the nucleobases are higher, however the glycosidic bond conformations of the bound states are still mostly *anti*. c-di-GMP mainly displays an extended conformation (Supplementary Fig. S18) when bound to the EAL domain based protein. The EAL domain originated from c-di-GMP-specific phosphodiesterases (PDEs) whose function is to hydrolyze c-di-GMP. The fully extended monomeric conformation of c-di-GMP is apparently more susceptible to cleavage of the ribose-phosphate ring^37–39^. The same binding mode of the extended monomeric c-di-GMP is retained in the enzymatically inactive EAL domains that function exclusively as c-di-GMP receptors^40–42^, which makes the bound states display large differences to the free state.

It has been observed that c-di-GMP can bind to protein in monomeric, dimeric and tetrameric forms^14^ (Supplementary Fig. S19). In the dimeric form, c-di-GMP molecules adopt a “U” shape like structure and intercalate with each other (Supplementary Fig. S19a). The c-di-GMP tetramer is composed of two dimers (Supplementary Fig. S19b). The driving force of the intercalation mostly comes from the stacking between guanine bases as well as hydrogen bonds between amino and imino proton of guanine with the phosphate group. Interestingly, the DFT optimized conformation of c-di-GMP shows high similarity with conformations in the dimeric and tetrameric forms, which suggests that the backbone conformation of c-di-GMP is pre-organized for intercalation. The comparison of the DFT optimized monomer and dimer conformations showed that the monomer conformation also resembles the conformations in the dimeric form (Supplementary Fig. S19c).

As for c-di-AMP, the comparison of the DFT optimized conformations to those bound to receptors such as tricarboxylic acid cycle enzyme pyruvate carboxylase, CTD domain, BsDisA diadenylate cyclase, phosphodiesterase PgpH HD domain and PstA (Fig. 7b and Supplementary Fig. S20) reveals backbone RMSD values below 1.0 Å, indicating similar backbone conformation. Molecules of c-di-AMP bound to CBS and PstA display overall RMSD values larger than 2.0 Å relative to DFT optimized structures, which can be attributed to differences in nucleobase orientations. For c-di-AMP bound to DarA, both overall and backbone RMSD values are around 3.0 Å indicating larger conformational change upon binding of free c-di-AMP to DarA.

For c-GAMP, the comparison of the DFT optimized structures with STING and riboswitch-bound conformations (see Fig. 7c and Supplementary Fig. S21) shows high similarity in the conformation of the macrocyclic moieties and differences in the orientation of the nucleobases.

### Conformational study of 2′-OH modification of c-di-GMP

Several 2′-OH modified analogues of c-di-GMP (Supplementary Fig. S22), which serve as potential inhibitors of c-di-GMP receptors^43^, have been subjected to REMD simulations. It has been shown that, in agreement with NMR measurement^43^, the substitution of 2′-OH by fluorine or methoxyl group does not affect the backbone conformation and phase angle of pseudorotation of c-di-GMP, while the substitution of 2′-OH by hydrogen results in unbiased sugar conformation (Supplementary Fig. S23). The glycosidic bonds of these analogues were shifted to *syn* conformations (Supplementary Fig. S23) resulting in a single major conformational state (Supplementary Fig. S24). The c-di-GMP_2′F, in which the 2′-OH groups were replaced by fluorine, displayed 4 times higher binding affinity to GGDEF I-site based diguanylate cyclase than c-di-GMP^43^. Since the backbone and sugar puckering are unaffected by the modification (see Supplementary Fig. S25–S26), the enhancement of the binding affinity may result from the fluorine-hydrogen bonding interactions between 2′-F and arginine in the I-site. The native CDN displayed 10 times higher binding affinity to EAL-based phosphodiesterases than c-di-GMP_2′F, which may be due to *syn* glycosidic bond conformations.

## Conclusions

In summary, we have systematically explored the conformational space of CDNs by computational and NMR studies. The ribose moieties of CDNs adopt predominantly N-type conformation, while the glycosidic bonds prefer *anti* conformations. The 12-membered rings formed by the backbone of CDNs are shown to be highly rigid even upon the 2′-OH modification of ribose. The conformations of macrocyclic moieties of free CDNs are very similar to conformations observed for CDNs bound to most of receptors. These findings shed light on molecular mechanisms underlying the activities of CDNs in a structural way and provide incentives for the design of small molecules to modulate CDN signalling pathways in bacteria or as vaccine adjuvants, that is, further study can focus on the modification of nucleobase or substitutions of the ribose with the aim to improve the binding constant or cell permeability of the CDN analogues. The rigidity of the backbone of CDNs also enables the design of high order structures such as molecular cages based on CDNs analogues. This study also shows that combination of REMD simulation and DFT calculations could efficiently probe the conformational space of CDNs and its analogues for further studies.

## Materials and Methods

### Replica exchange molecular dynamics simulations

Replica exchange molecular dynamics (REMD) simulation is an advanced sampling technique that promotes efficient conformational sampling by enhancing the probability of sampling high-energy configurations at elevated temperatures. Several identical copies (replicas) of the system are run in parallel, each differing in temperature. The neighboring replicas may exchange their temperature states based on a Boltzmann-weighted probability. Replicas are allowed to communicate at regular intervals during which exchange attempts are made based on a Monte Carlo criterion. When this condition is satisfied an exchange attempt is considered successful, and the conformations in neighboring replica temperatures are swapped. The velocity of the corresponding replica is then rescaled to the new replica temperature. The process is repeated iteratively during the simulation such that each replica evolves with a wide range of temperatures, enhancing conformational sampling. At present study, a 75% exchange rate is observed for all of the CDNs.

The partial charges for CDNs were generated by geometry optimization and electrostatistic potential (ESP) calculations with Gaussian 09^44^ at the level of HF/6–31 G* by the RED server^45–47^. Other force field parameters were taken from Amber ff14SB force field basic version parm99^48^ with the bsc0^49^ and χOL3 refinements^50,51^.

Simulations were performed with AMBER 14 molecular modeling package^52,53^. The structures were subjected to energy minimization for 2000 cycles, where first 500 cycles were performed by steepest descent energy minimization, and the remaining 1500 cycles were minimized by conjugate gradient minimization. The minimized structure was used to generate chirality constraints to prevent unwanted rotation around the backbone bonds, which might occur at higher temperature during the REMD simulation. The generalized Born implicit solvation model^54,55^ and NVT ensembles were used in the REMD. The SHAKE algorithm^56^ was used to constrain the bond stretching freedom of all bonds involving hydrogens, and the nonbonded van der Waals and electrostatic cutoffs were taken as 999 Å. For REMD, 24 replica temperatures were used (273.0, 286.5, 300.0, 313.5, 327, 340.5, 354, 367.5, 381, 394.5, 408.0, 421.5, 435, 448.5, 462.0, 475.5, 489.0, 502.5, 516.0, 529.5, 543, 556.5, 570.0 and 583.5 K). Replica temperatures were maintained by weak coupling to the Langevin thermostat with a collision frequency of 1 ps^−1^. Prior to REMD simulations, the system was equilibrated for 200 ps, during which the temperature of each replica was gradually increased from 0 to the target temperature of that replica. After equilibration, REMD simulation was performed using the multisander module. The integration step for the production run was 0.002 ps. Replica temperature exchange attempts were performed every 1.5 ps. The output and coordinate files were saved every 1 ps, and the total length for each of the simulations was 60 ns. All trajectories were processed with cpptraj to filter the trajectory corresponding to 300 K.

We also performed REMD simulations in explicit solvent. The CDNs were solvated with TIP3P water molecules in a truncated octahedron periodic box and the total system charge was neutralized with magnesium ions. Energy minimization was performed for 1000 steps with the steepest descent algorithm followed by 4000 steps with the conjugate gradient algorithm. After minimization, the systems were heated from 0 K to the desired temperature in 50 ps of each replica at constant volume with 10 kcal/mol Å^2^ atomic positional restraints on CDNs. The temperature was controlled using a Langevin thermostat with a collision frequency of 2.0 ps^−1^. Prior to production simulations, a 200 ps equilibrium period was employed to equilibrate each replica. The parameters for the production runs were identical to the implicit solvent simulations.

### DFT calculation

The DFT calculations were executed using Gaussian 09^43^. The geometries of CDNs were optimized at the B3LYP/6–31 G(d,p) level and incorporated Tomasi′s Polarized Continuum Model (PCM) corrections for water as the bulk solvent. Frequency calculations for all stationary points were carried out to confirm them as minima (i = 0).

### NMR experiment

Spectra were recorded on Agilent Technologies DD2 600 MHz NMR spectrometer equipped with ^1^H{^13^C,^15^N} cold probe. Phosphorus decoupled ^1^H NMR spectra and spectra at 80 °C were recorded on the same spectrometer using OneNMR probe. NOESY and ROESY spectra were recorded at 20 °C on Agilent Technologies DD2 300 MHz NMR spectrometer equipped with ID/PFG probe with various mixing times between 60 and 400 ms for samples of each of the dinucleotides. For NMR experiments samples were dissolved in a TRIS/HCl buffer with 100 mM NaCl and 5 mM MgCl_2_ in D_2_O at pH 7.4 to a concentration between 2 and 6 mM. ^1^H, ^13^C, TOCSY, COSY, ^1^H-^13^C HSQC and ^1^H-^13^C HMBC spectra were recorded for assignment. J_H-H_ and J_H-P_ coupling constants were determined from analysis of multiplet structure in ^1^H spectra. ^31^P decoupled ^1^H NMR spectra were recorded to facilitate interpretation of multiplets involving J_HP_ couplings. ^1^H spectra were recorded between 0 and 80 °C in 20 °C steps. J_C-P_ values constants were evaluated from ^1^H decoupled ^13^C NMR spectra recorded at 20 °C.

Orientations of the χ dihedral angles were estimated from cross-peaks in NOESY spectra as well as a series of transient 1D NOESY spectra. 1D NOESY experiments were recorded at 20 °C using the NOESY1D pulse sequence. NOE build-up curves were measured to determine the optimal mixing times in the linear regime. The spectra were recorded with mixing times of 0.6 s for all CDNs except c-di-GMP, for which a mixing time of 0.35 s was employed.

^3^J_H-H_ coupling constants values were fitted to a mixture of two dominant conformers and optimized using a Pseurot^57^ based software for pseudorotation analysis of saturated five-membered ring systems implemented in Matlab^58^. The minor (S-type) conformation was kept fixed at P_S_ 163° and ψ_S_ 38° for the optimization, while P_N_, ψ_N_ and fractions of both populations were optimized. γ torsion angles were estimated from ^3^J_H4′-H5′_ and ^3^J_H4′-H5″_ coupling constants. The presence of three conformations of γ torsion angle at 53°, 180° and −70° was assumed. Values of scalar coupling constants were calculated for these angles and fractions were optimized for the best match to experimental values.

β and ε angles were estimated from the relevant J_C-P_ and J_H-P_ coupling constants. J_C-P_ coupling constants were analyzed according to parametrization of the Karplus equation offered by Plavec and Chattopadhyaya^29^.

## Electronic supplementary material

Supplementary Information
