# Supplementary material for: Computational and NMR spectroscopy insights into the conformation of cyclic di-nucleotides
Source: Sci Rep. 2017 Nov 29;7:16550. doi: 10.1038/s41598-017-16794-4 (PMC5707406; doi:10.1038/s41598-017-16794-4)
Supplement: Supplementary file 1 — Supplementary Information [file 41598_2017_16794_MOESM1_ESM.pdf]

Computational and NMR spectroscopy insights into the conformation of cyclic di-nucleotides

Baifan Wang,[1] Zhenghua Wang,[2] Uroš Javornik,[1] Zhen Xi[2] and Janez Plavec[1,3,4]

[1]Slovenian NMR Center, National Institute of Chemistry, Hajdrihova 19, Ljubljana, Slovenia. [2]State Key Laboratory of Elemento-Organic Chemistry and Department of Chemical Biology, Nankai University. Collaborative Innovation Center of Chemical Science and Engineering, Tianjin 300071, P. R. China. [3]EN-FIST Center of Excellence, Trg OF 13, 1000 Ljubljana, Slovenia. [4]Faculty of Chemistry and Chemical Technology, University of Ljubljana, Večna pot 113, Ljubljana, Slovenia. Correspondence and requests for materials should be addressed to Z.X. (email: zhenxi@nankai.edu.cn) or J.P. (email: janez.plavec@ki.si)

Electronic Supplementary Information

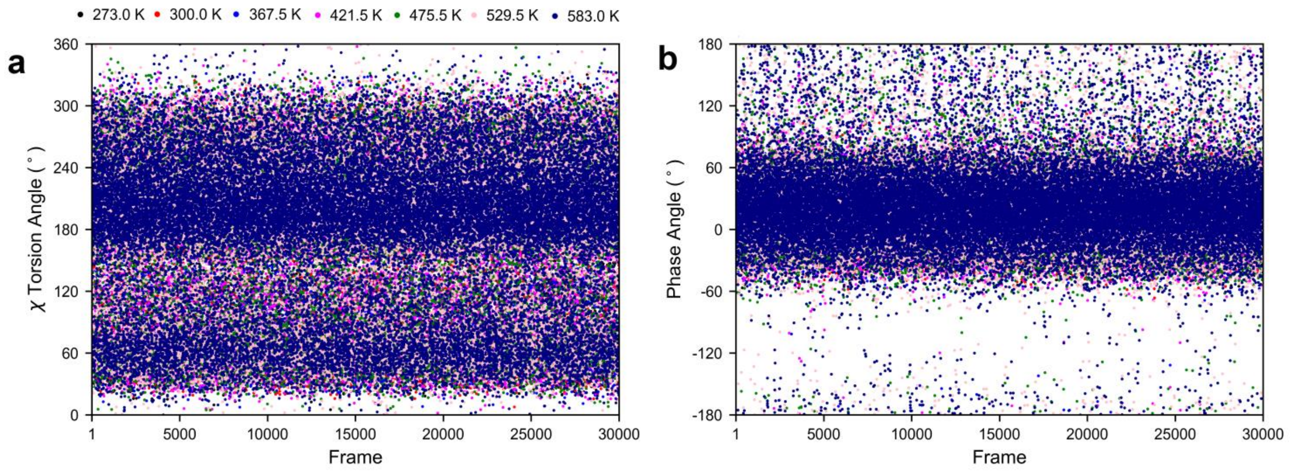

Figure S1. Distributions of χ torsion angle (**a**) and phase angles of pseudorotation (**b**) for c-di-GMP under various temperatures from REMD simulations in implicit solvent.

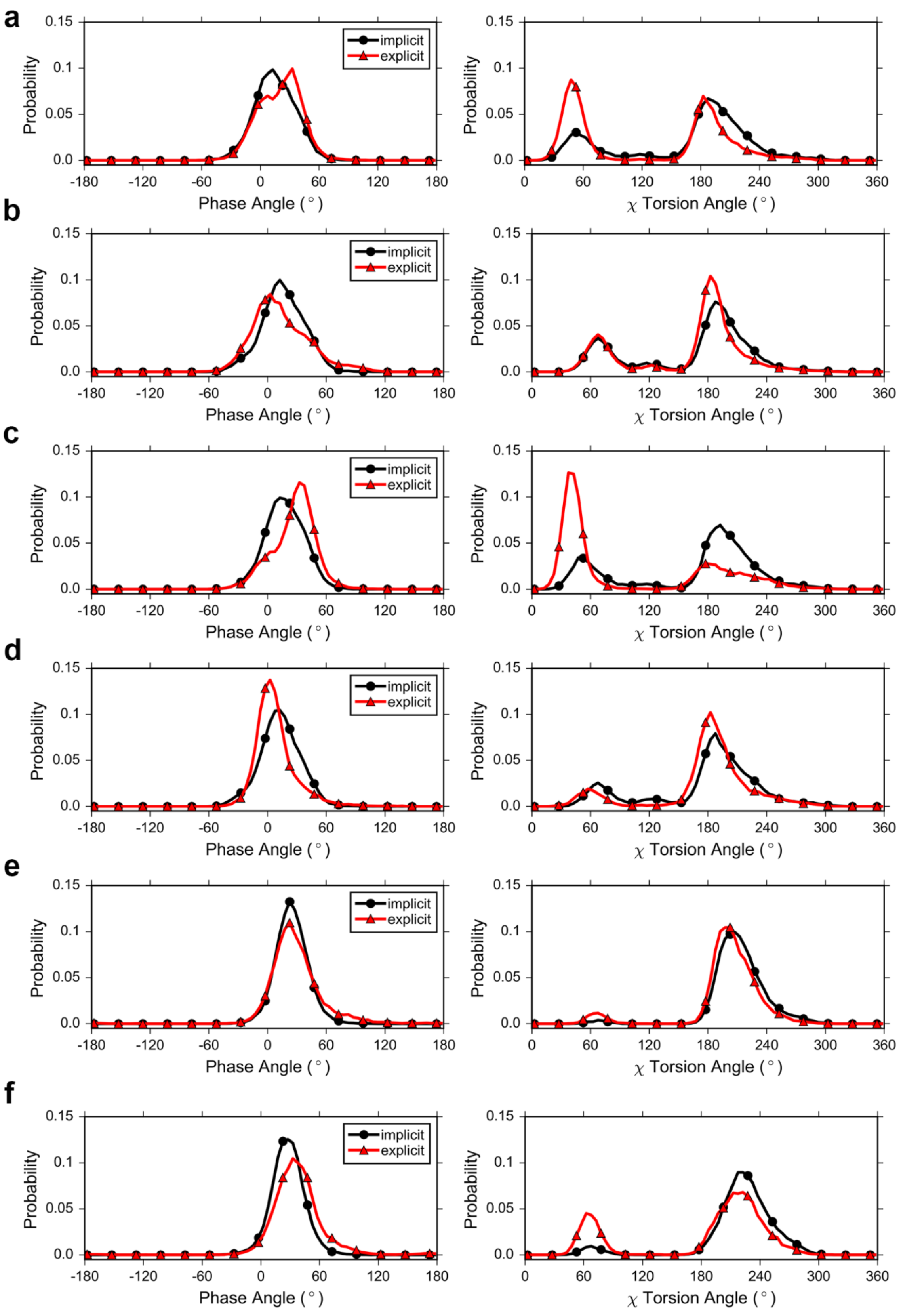

Figure S2. The probability distribution of phase angle of pseudorotation (left panel) and χ torsion angle (right panel) for CDNs from REMD simulations in implicit and explicit solvents. Data were obtained at 300 K. **a**: c-di-GMP, **b**: c-di-AMP, **c**: c-GAMP_G, **d**: c-GAMP_A, **e**: c-di-CMP, **f**: c-di-UMP.

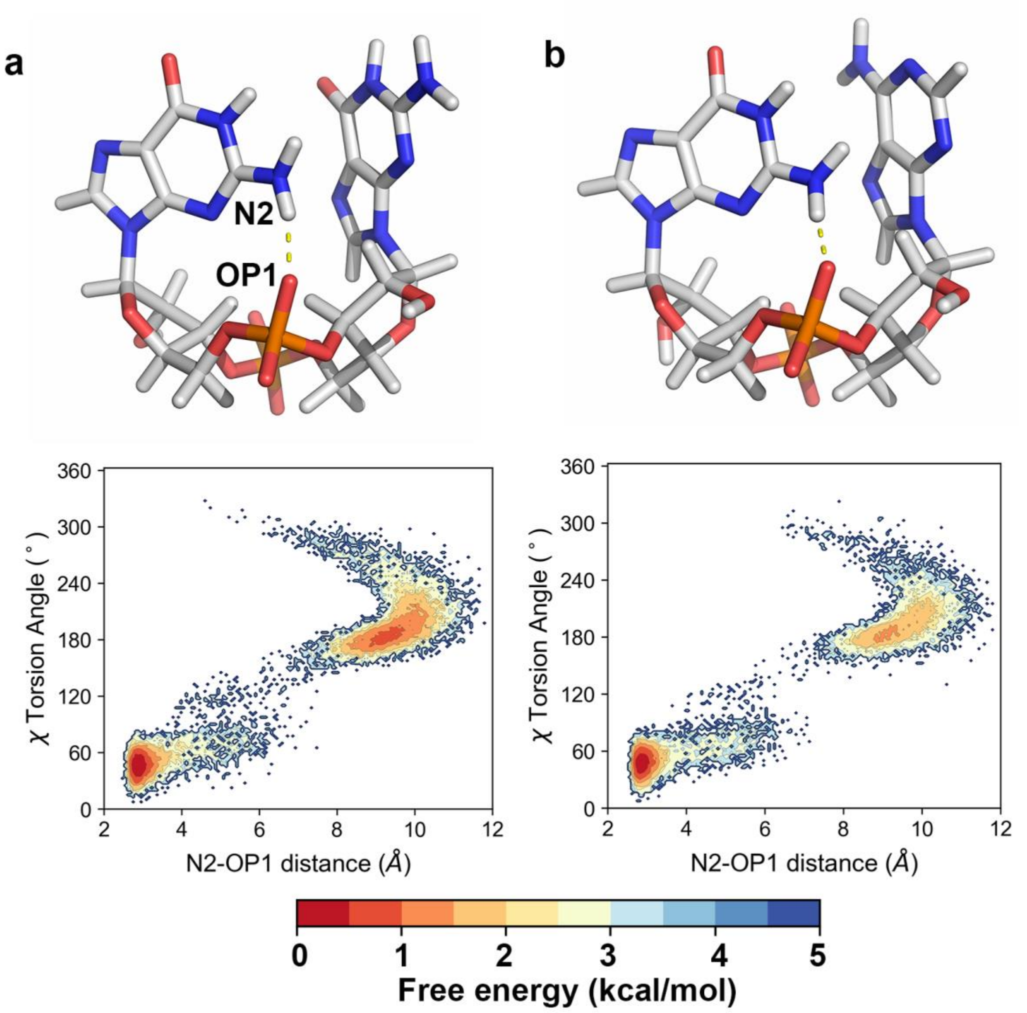

Figure S3. Formation of hydrogen bonds between amino and phosphate groups of guanine moiety in (**a**) c-di-GMP and (**b**) c-GAMP. The lower panel displays the population based free energy plot on χ-N2_to_OP1 distance plane from REMD simulations in explicit solvent at 300 K. Free energy estimates were calculated using the following equation: $G_i = -k_BT*\ln(N_i/N_{max})$, where k_B is Boltzmann's constant, T is temperature, N_i is the population of bin i and N_{max} is the population of the most populated bin.

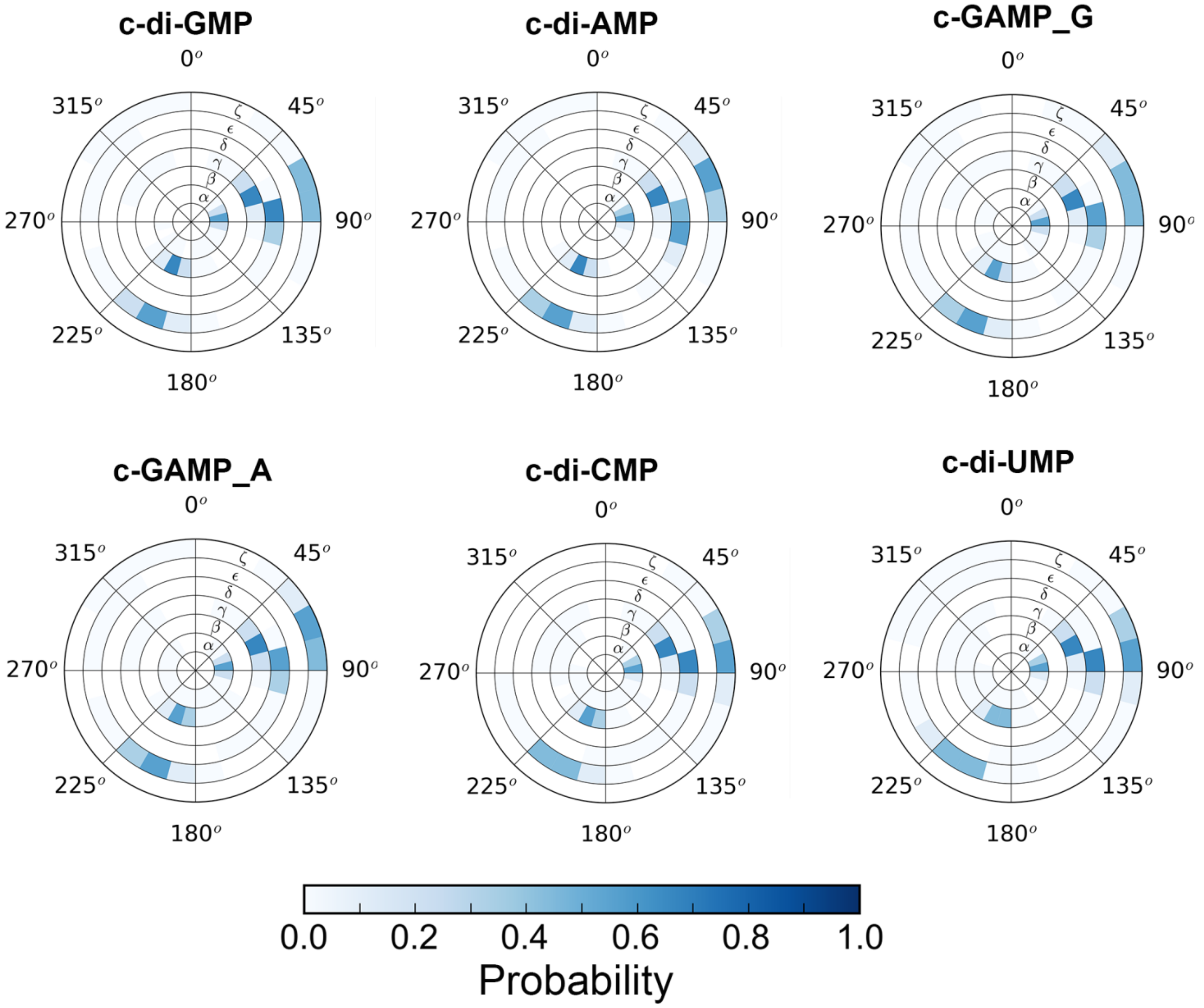

Figure S4. Conformational wheels for CDNs form REMD simulations in explicit solvent at 300 K.

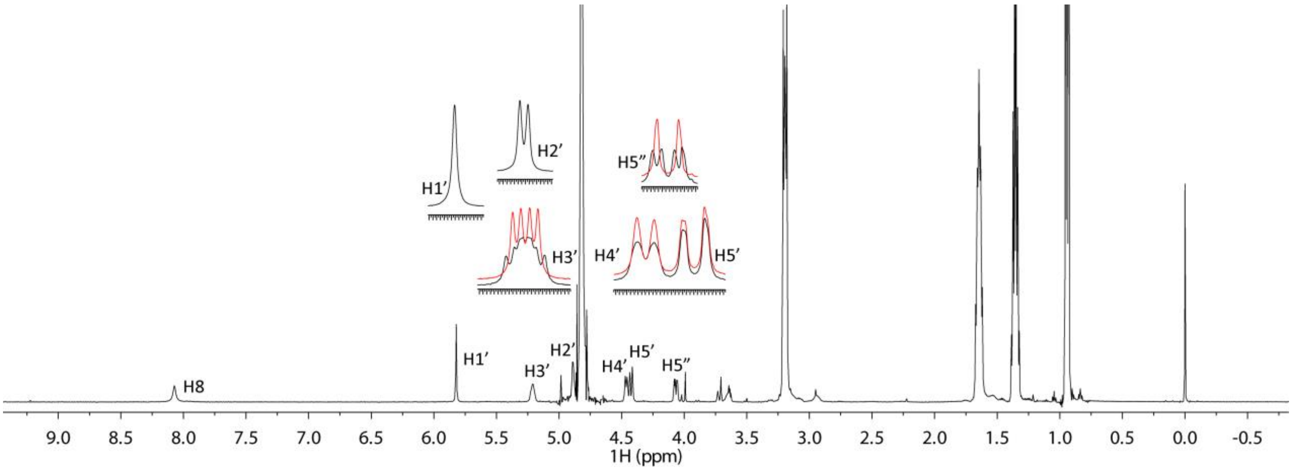

Figure S5. ^1H NMR spectrum of c-di-GMP in TRIS/HCl buffer with 100 mM NaCl and 5 mM $MgCl_2$ in ^2H_2O at pH 7.4 and 20 °C. Expansions of ribose ring proton signals are displayed above the spectrum with traces of ^{31}P decoupled ^1H NMR spectra (in red) overlaid for signals affected by ^1H-^{31}P scalar coupling. Black traces correspond to ^{31}P coupled spectra.

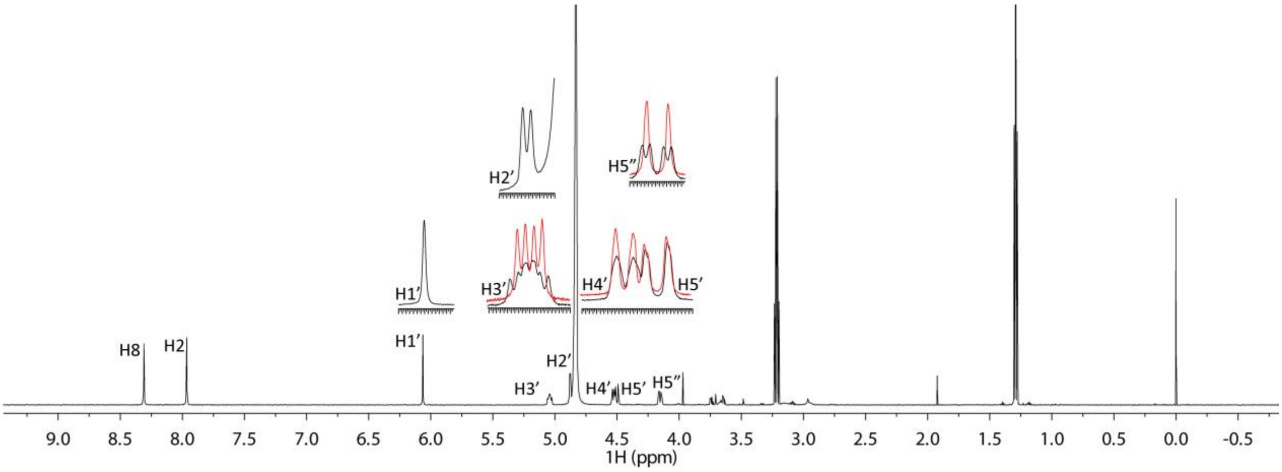

Figure S6. ^{1}H NMR spectrum of c-di-AMP in TRIS/HCl buffer with 100 mM NaCl and 5 mM $MgCl_2$ in D_2O at pH 7.4 and 20 °C. Expansions of ribose ring proton signals are displayed above the spectrum with traces of ^{31}P decoupled ^{1}H NMR spectra (in red) overlaid for signals affected by ^{1}H-^{31}P scalar coupling. Black traces correspond to ^{31}P coupled spectra.

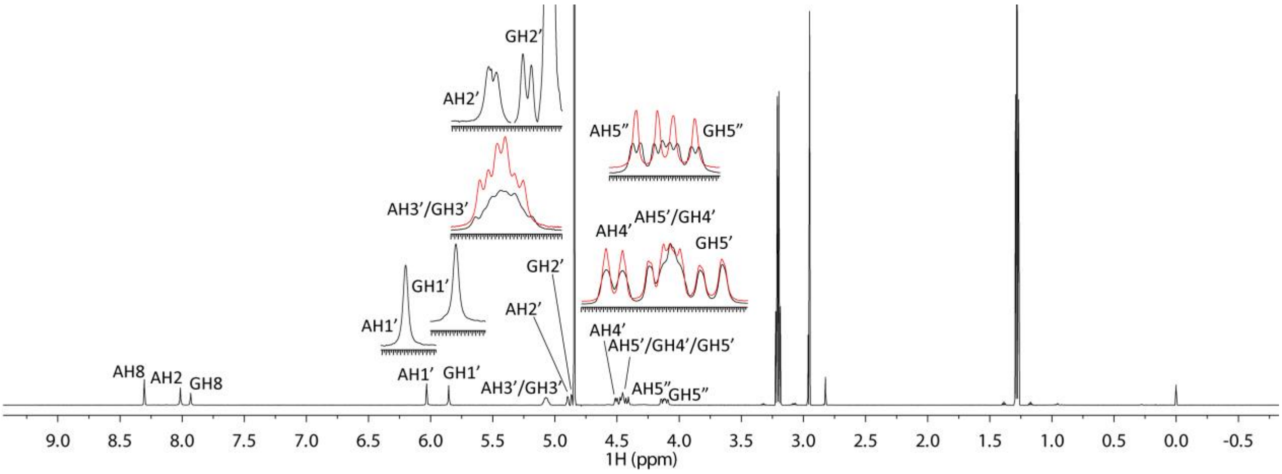

Figure S7. ^{1}H NMR spectrum of c-GAMP in TRIS/HCl buffer with 100 mM NaCl and 5 mM $MgCl_2$ in D_2O at pH 7.4 and 20 °C. Expansions of ribose ring proton signals are displayed above the spectrum with traces of ^{31}P decoupled ^{1}H NMR spectra (in red) overlaid for signals affected by ^{1}H-^{31}P scalar coupling. Black traces correspond to ^{31}P coupled spectra.

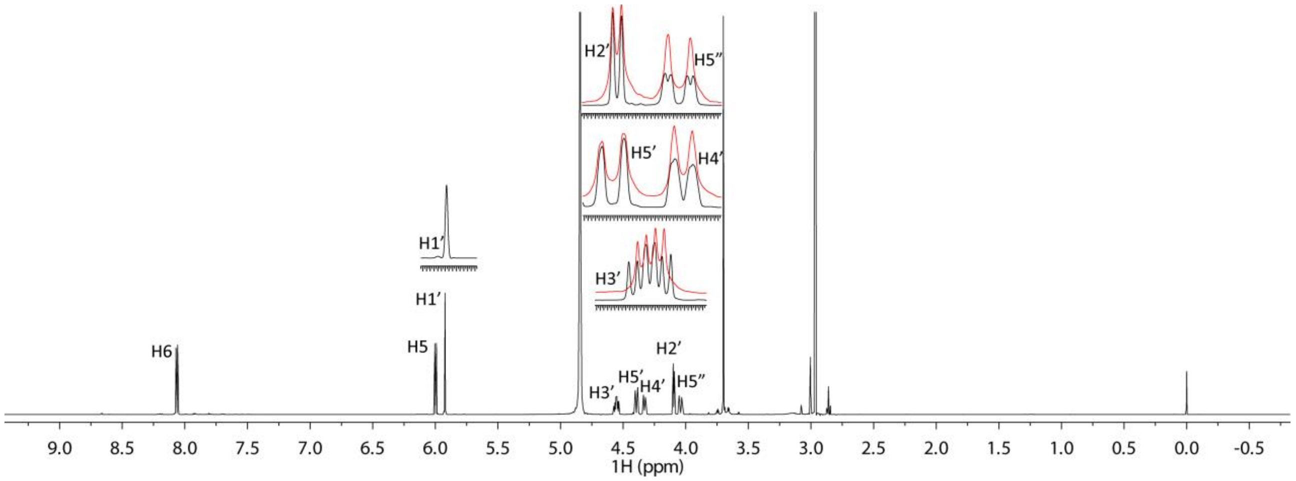

Figure S8. ^{1}H NMR spectrum of c-di-CMP in TRIS/HCl buffer with 100 mM NaCl and 5 mM $MgCl_2$ in D_2O at pH 7.4 and 20 °C. Expansions of ribose ring proton signals are displayed above the spectrum with traces of ^{31}P decoupled ^{1}H NMR spectra (in red) overlaid for signals affected by ^{1}H-^{31}P scalar coupling. Black traces correspond to ^{31}P coupled spectra.

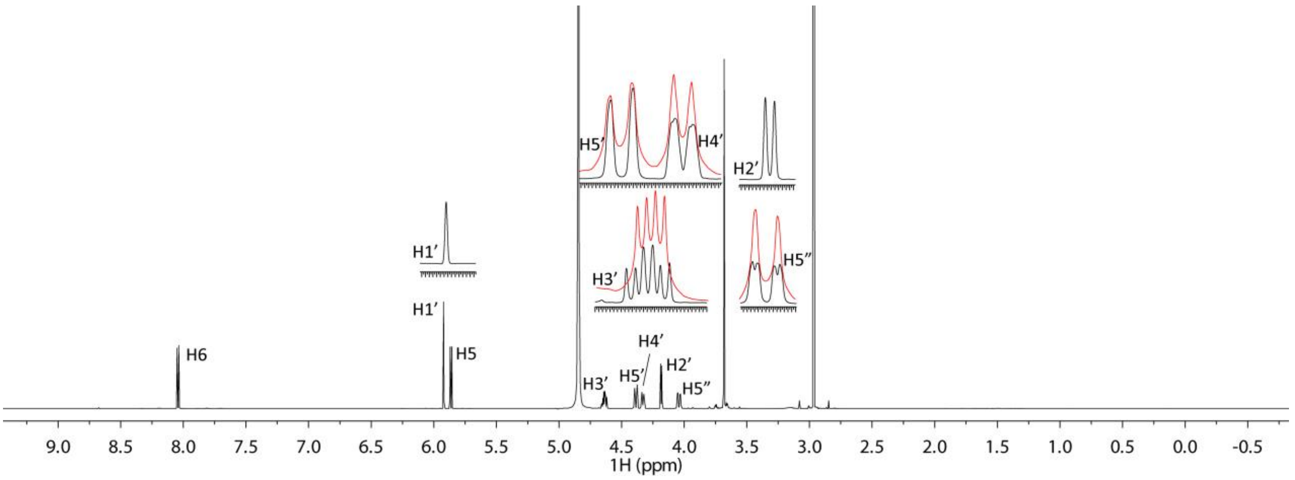

Figure S9. ^{1}H NMR spectrum of c-di-UMP in TRIS/HCl buffer with 100 mM NaCl and 5 mM $MgCl_2$ in D_2O at pH 7.4 at 20 °C. Expansions of ribose ring proton signals are displayed above the spectrum with traces of ^{31}P decoupled ^{1}H NMR spectra (in red) overlaid for signals affected by ^{1}H-^{31}P scalar coupling. Black traces correspond to ^{31}P coupled spectra.

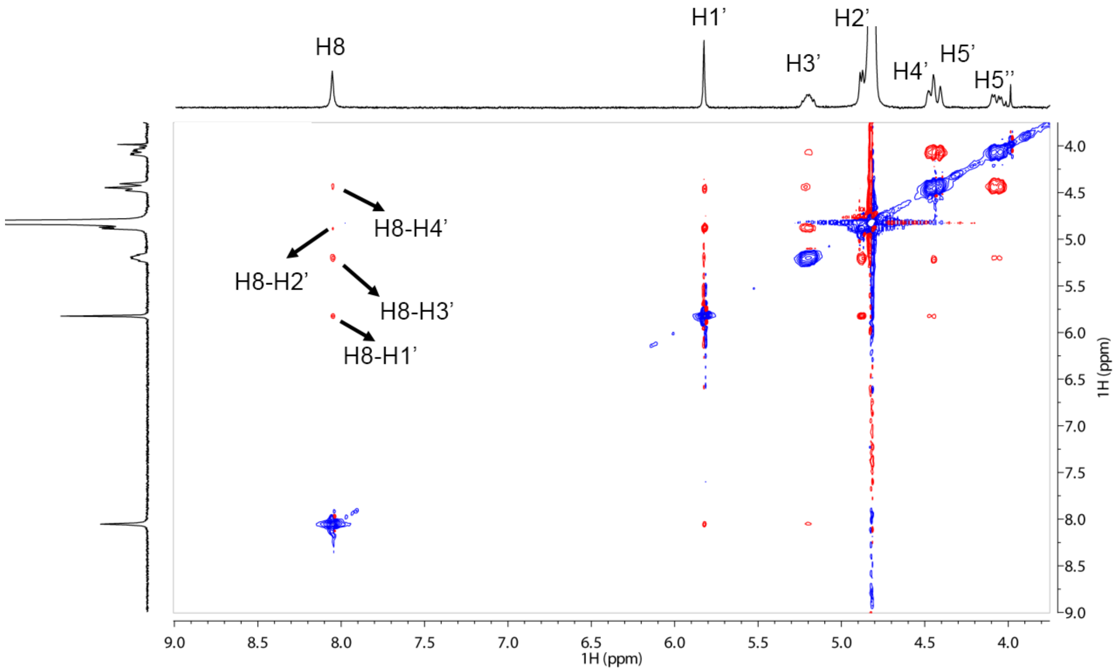

Figure S10. NOESY spectrum (200 ms mixing time) of c-di-GMP in TRIS/HCl buffer with 100 mM NaCl and 5 mM $MgCl_2$ in D_2O at pH 7.4 and 20 °C.

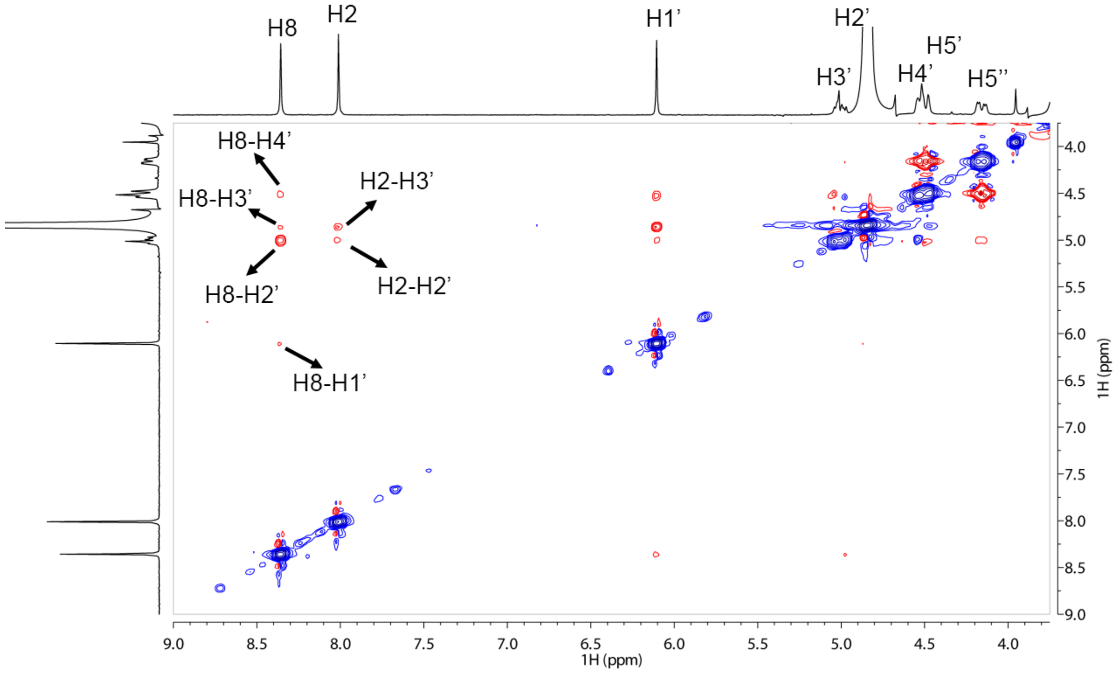

Figure S11. ROESY spectrum (120 ms mixing time) of c-di-AMP in TRIS/HCl buffer with 100 mM NaCl and 5 mM $MgCl_2$ in D_2O at pH 7.4 and 20 °C.

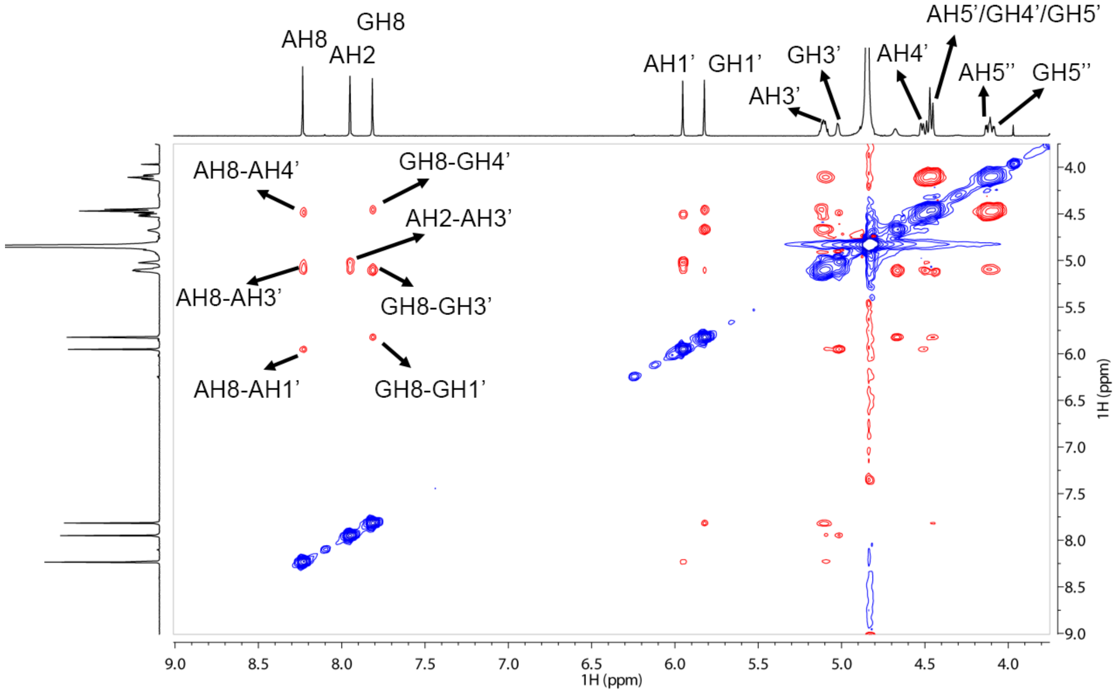

Figure S12. NOESY spectrum (200 ms mixing time) of c-GAMP in TRIS/HCl buffer with 100 mM NaCl and 5 mM $MgCl_2$ in D_2O at pH 7.4 and 20 °C.

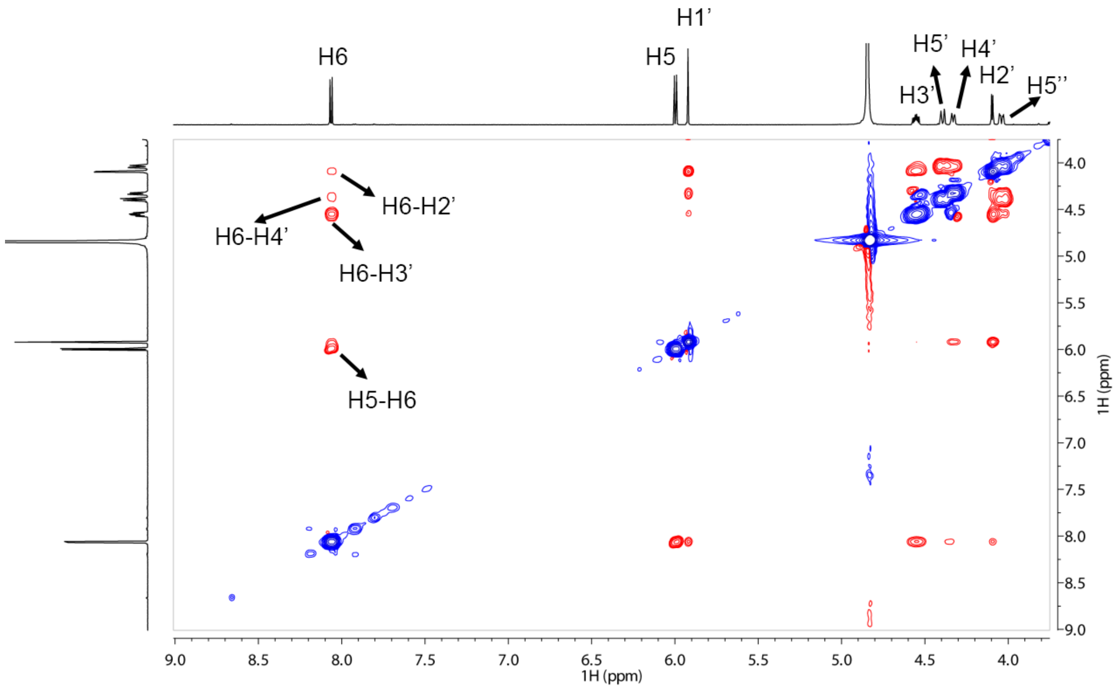

Figure S13. NOESY spectrum (400 ms mixing time) of c-di-CMP in TRIS/HCl buffer with 100 mM NaCl and 5 mM MgCl2 in D_2O at pH 7.4 and 20 °C.

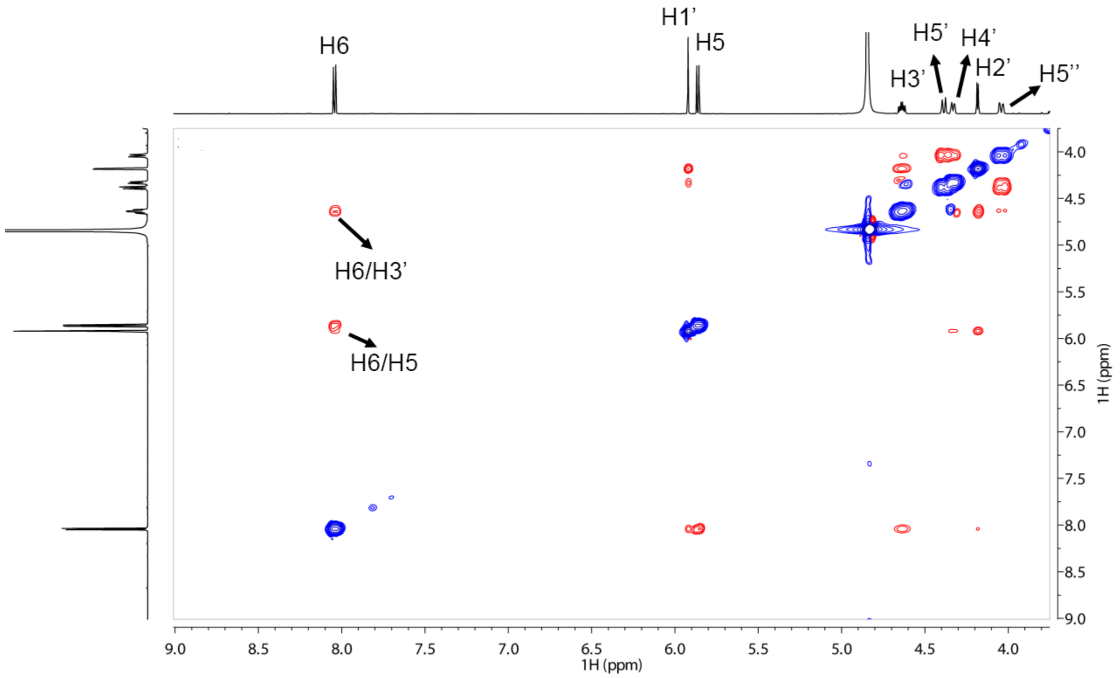

Figure S14. NOESY spectrum (400 ms mixing time) of c-di-UMP in TRIS/HCl buffer with 100 mM NaCl and 5 mM $MgCl_2$ in D_2O at pH 7.4 and 20 °C.

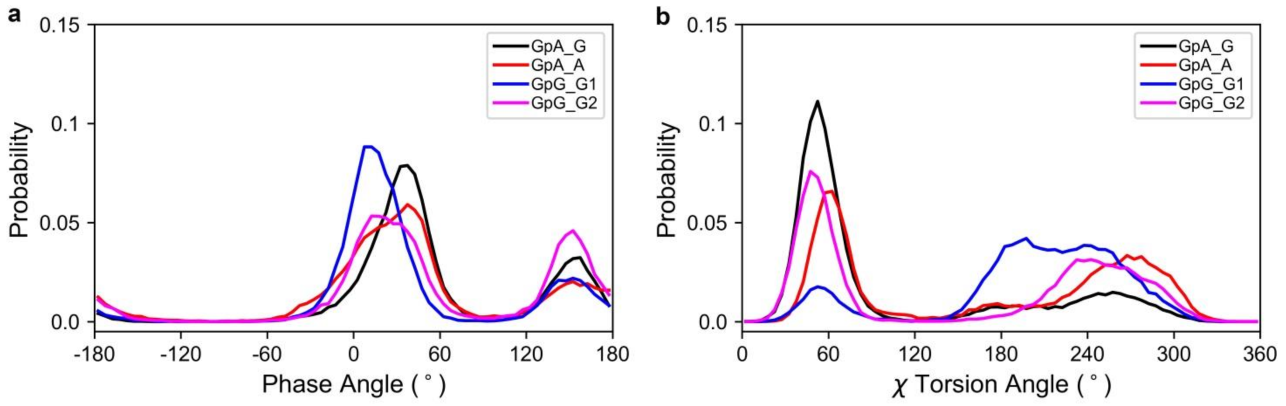

Figure S15. Probability distribution of ribose pseudorotation phase angle (**a**) and χ torsion angle (**b**) for di-nucleotides from REMD simulation in implicit solvent at 300 K.

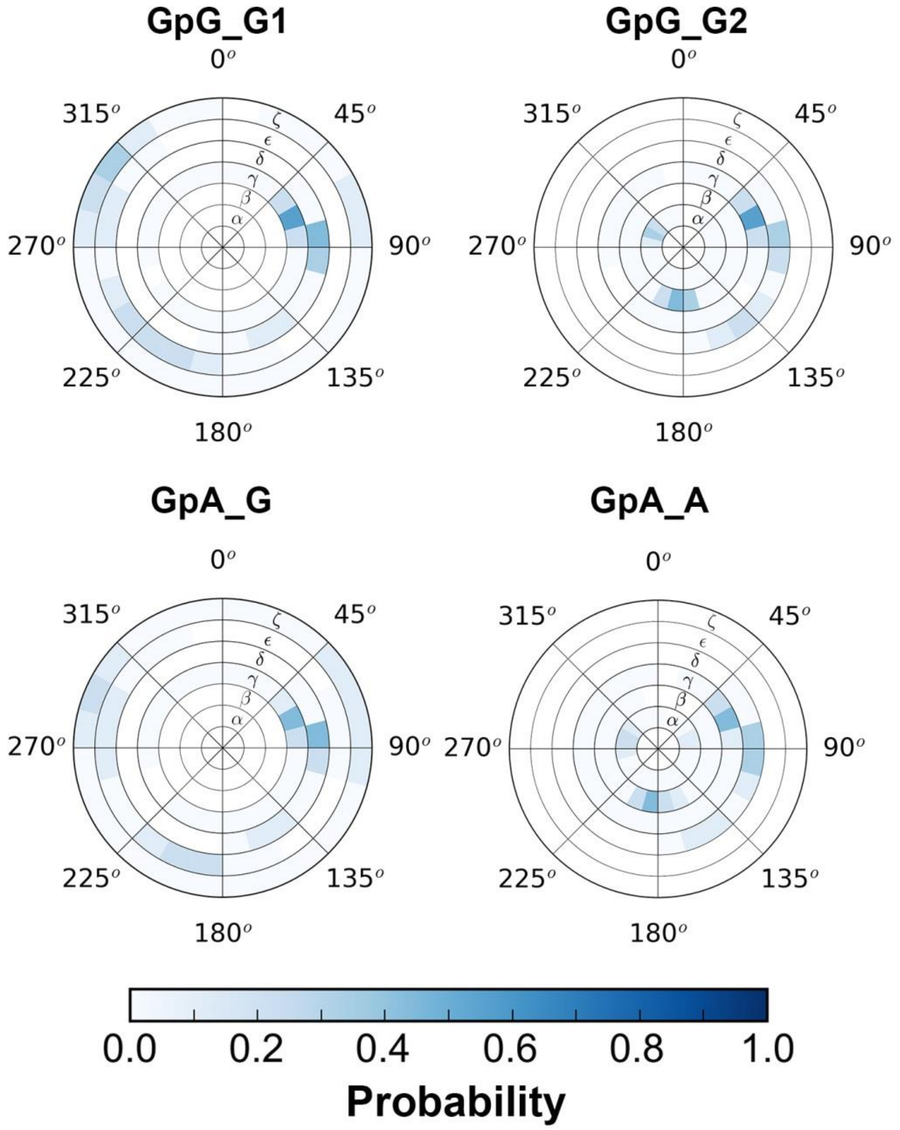

Figure S16. Conformational wheels for di-nucleotides form REMD simulation at 300 K.

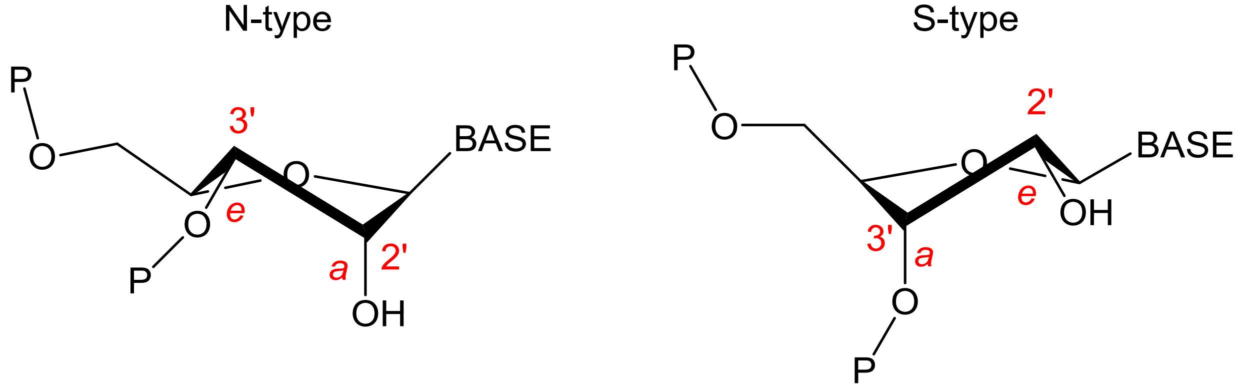

Figure S17. Representation of the Northern (N-type) and Southern (S-type) conformations of ribose moiety with pseudo-axial (a) and pseudo-equatorial (e) positions of the substitutions attached to C2' and C3' atoms.

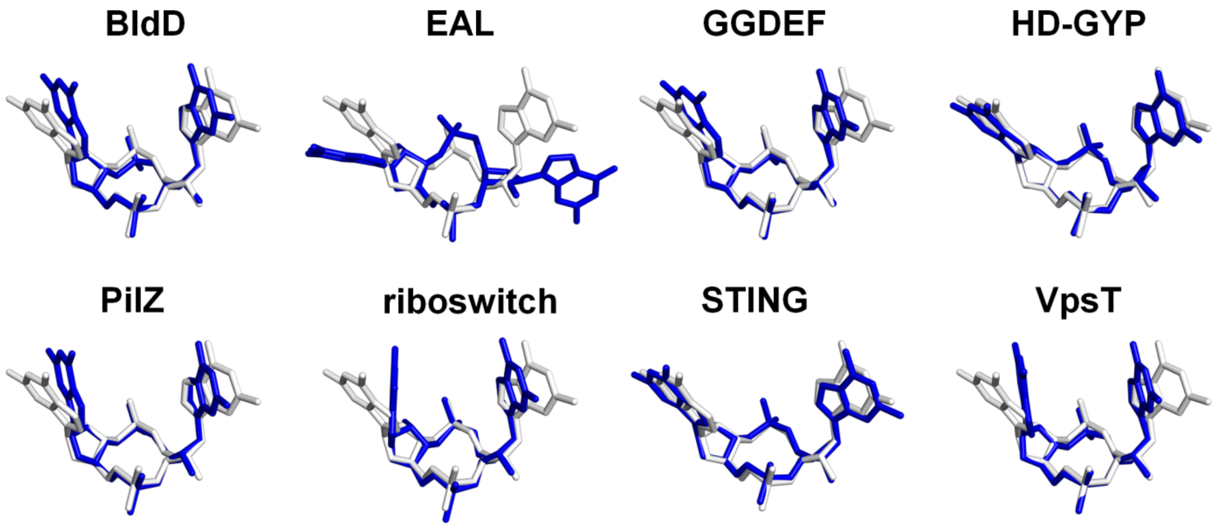

Figure S18. Comparison of the structural similarity between DFT optimized (colored in grey) and various receptor-bound conformations (colored in blue, the type of receptors are labeled) of c-di-GMP.

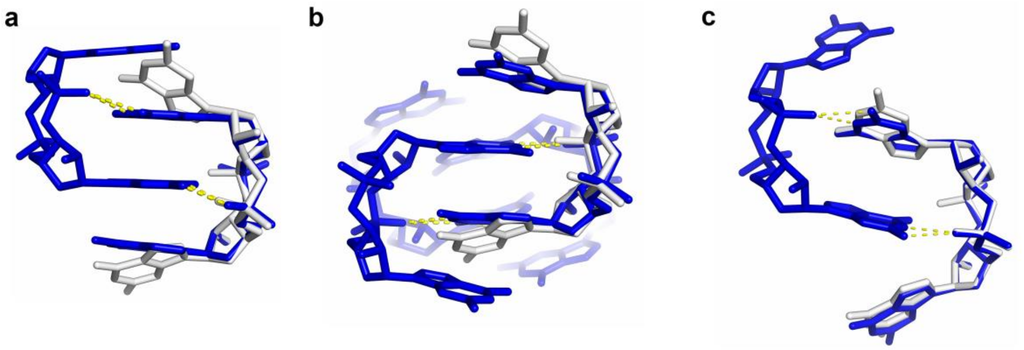

Figure S19. Comparison of the lowest energy structure of c-di-GMP (grey) with (**a**) its dimer conformation (blue) bound to GGDEF I site domain (PDB ID: 4DN0), (**b**) tetrameric form conformation bound to BldD domain (PDB ID: 5KHD) and (**c**) DFT optimized dimer conformation. The hydrogen bonds are shown in yellow dotted lines.

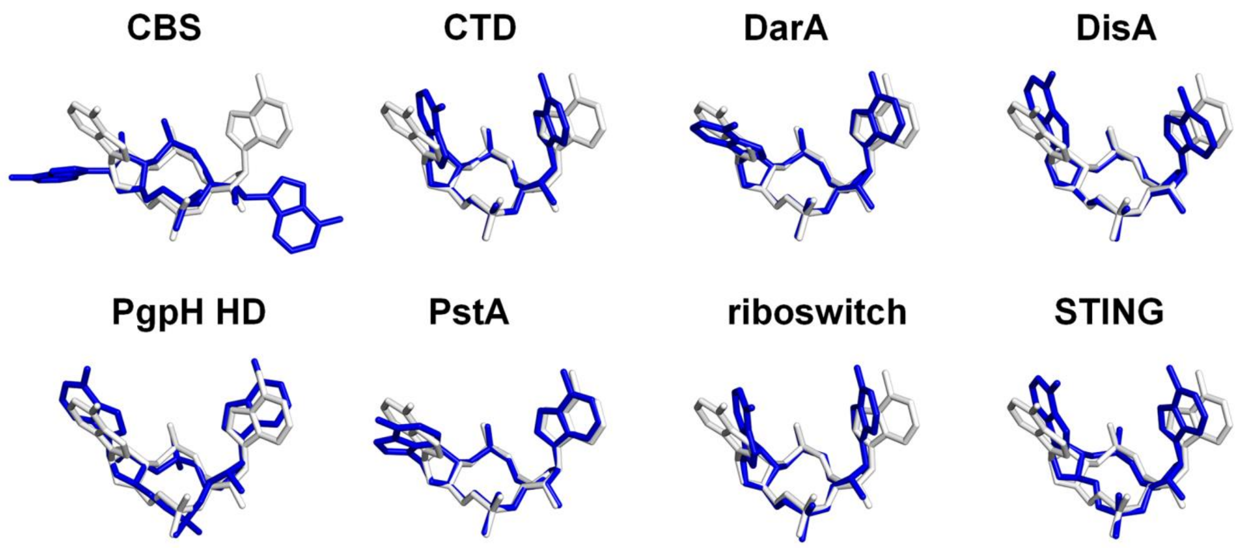

Figure S20. Comparison of the structural similarity between DFT optimized (colored in grey) and various receptor-bound conformations (colored in blue, the type of receptors are labeled) of c-di-AMP

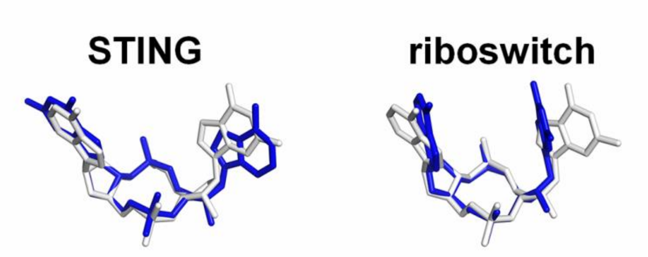

Figure S21. Comparison of the structural similarity between DFT optimized (colored in grey) and various receptor-bound conformations (colored in blue, the type of receptors are labeled) of c-GAMP.

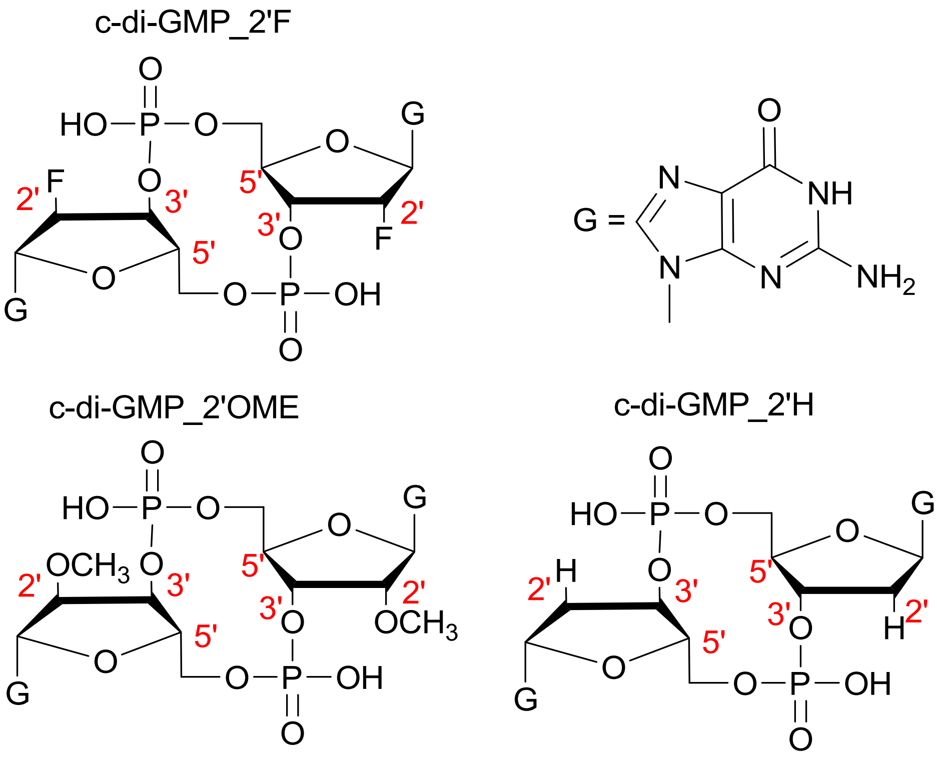

Figure S22. Chemical structures of c-di-GMP analogues with 2'-OH modification

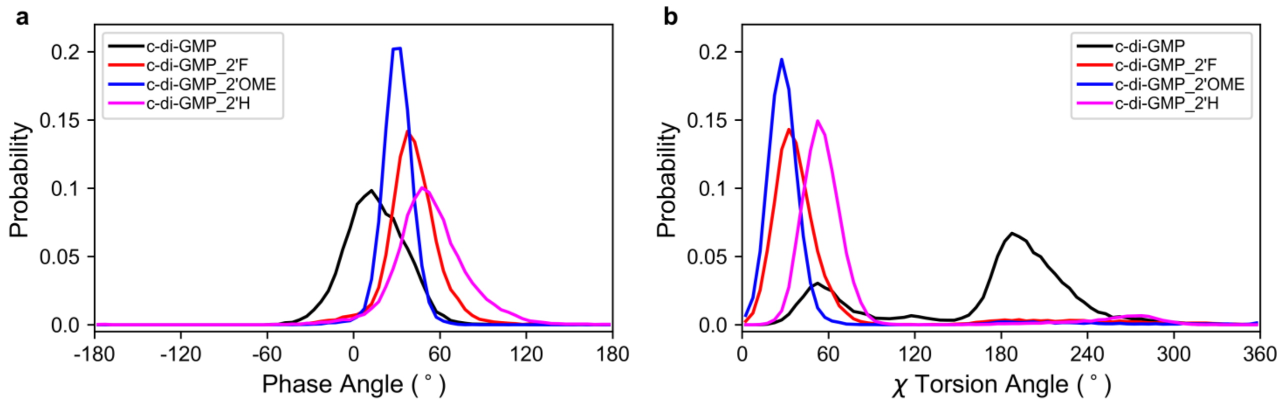

Figure S23. Probability distribution of phase angles of pseudorotation (**a**) and χ torsion angle (**b**) for c-di-GMP analogues with 2'-OH modification from REMD simulation at 300 K.

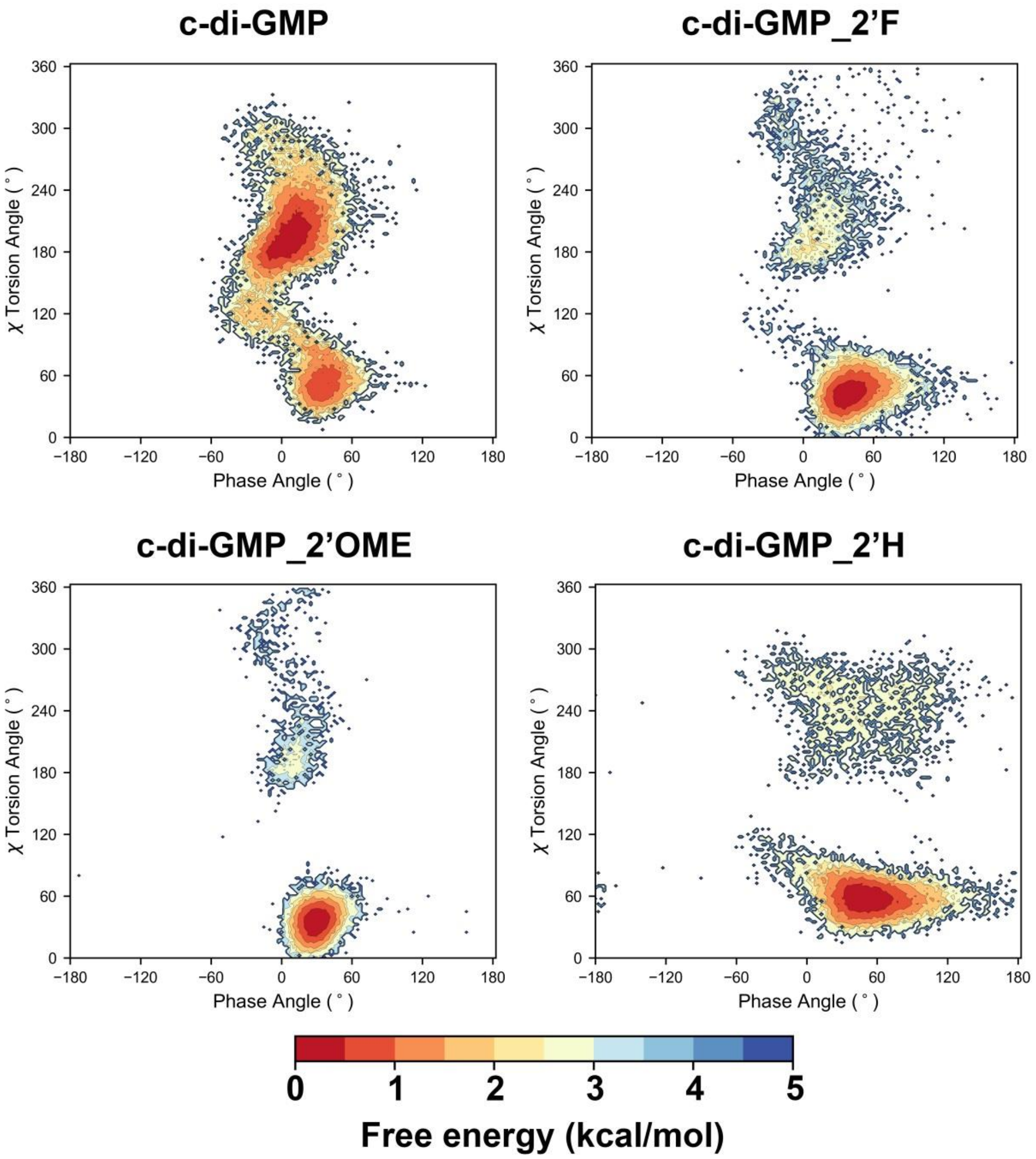

Figure S24. Population based free energy plot based on the χ-phase angles plane for c-di-GMP analogues with 2'-OH modification REMD simulation at 300 K.

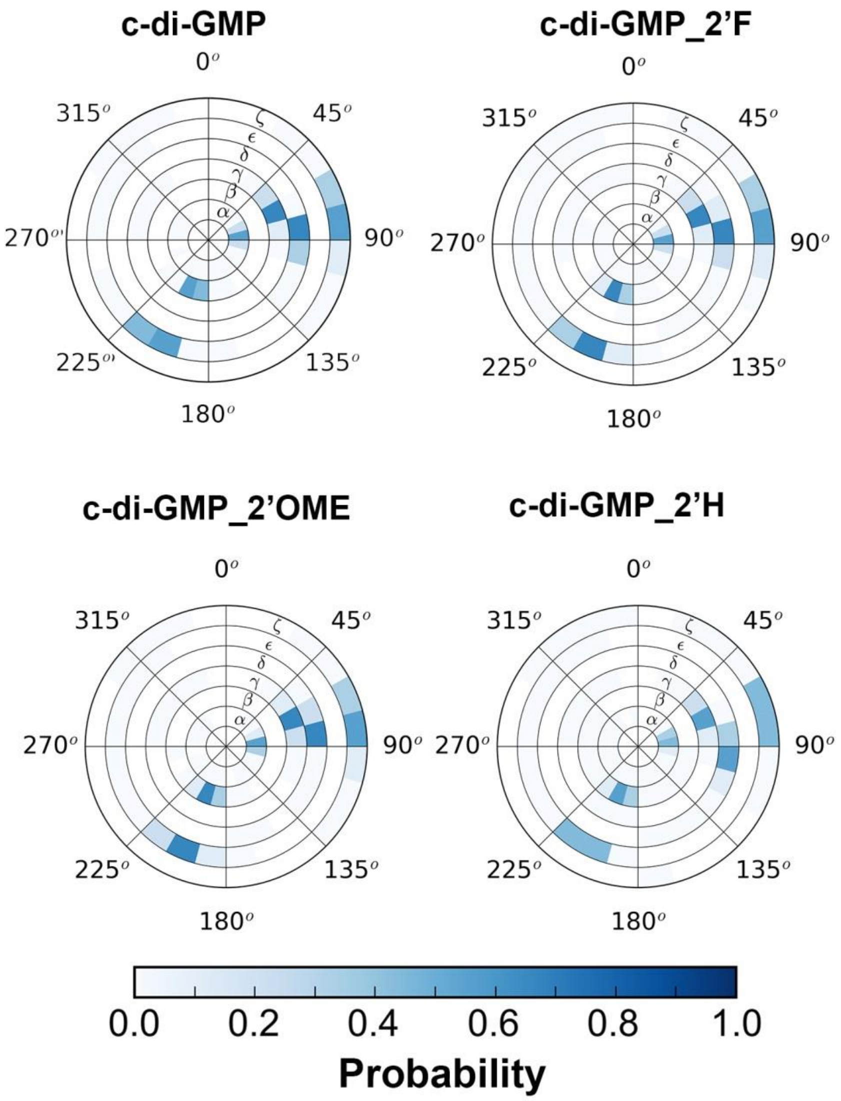

Figure S25. Conformational wheels for c-di-GMP analogues with 2'-OH modification form REMD simulation at 300 K.

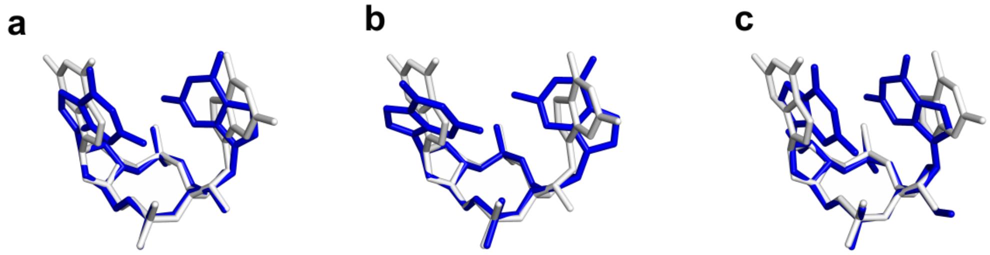

Figure S26. Comparison of the lowest energy structures of c-di-GMP analogues with 2'-OH modification to native CDN. c-di-GMP is shown in grey, while its analogues are colored in blue. (**a**): c-di-GMP_2'F; (**b**): c-di-GMP_2'OME; (**c**): c-di-GMP_2'H.

Table S1. $^3J_{H\text{-}H}$ coupling constants (Hz) defining the sugar pucker and γ torsion angle populations of N-type conformers and γ^+ at different temperatures for CDNs.

CDN	T [K]	$J_{H1'\text{-}H2'}$	$J_{H2'\text{-}H3'}$	$J_{H3'\text{-}H4'}$	% N-type[b]	$J_{H4'\text{-}H5''}$	$J_{H4'\text{-}H5'}$	% γ^+
c-di-GMP	293	n.r.	4.4	9.3	100	2.0	1.1	>98%
	313	n.r.	4.5	9.1	100	2.1	1.2	>98%
	333	n.r.	4.7	8.6	97	2.6	1.7	94%
	353	n.r.	4.8	8.2	95	2.9	1.9	89%
c-di-AMP	273	n.r.	4.3	9.3	100	n.r.	n.r.	/
	293	n.r.	4.4	9.2	100	2.1	n.r.	>98%[b]
	313	n.r.	4.5	9.2	100	2.3	1.4	>98%
	333	n.r.	4.6	8.8	97	/[a]	1.8	/
c-GAMP (Guanine moiety)	273	n.r.	/[a]	/[a]	/	/[a]	n.r.	/
	293	n.r.	3.4	/[a]	/	/[a]	n.r.	/
	313	n.r.	/[a]	/[a]	/	/[a]	1.4	/
	333	n.r.	4.8	8.2	95	/[a]	/[a]	/
	353	1.9	5.0	7.9	88	/[a]	1.9	/
c-GAMP (Adenine moiety)	273	n.r.	/[a]	8.9	100	1.9	n.r.	>98%
	293	n.r.	4.6	8.9	100	1.5	n.r.	>98%
	313	n.r.	4.7	8.9	/	1.6	1.4	>98%
	333	n.r.	4.8	8.8	100	n.r.	1.7	/
	353	n.r.	4.8	8.5	93	n.r.	2.1	/
c-di-CMP	273	n.r.	4.7	9.8	100	1.5	n.r.	/
	293	n.r.	4.8	9.8	100	1.7	1.2	>98%
	313	n.r.	4.8	9.8	100	1.7	1.4	>98%
	333	n.r.	4.8	9.7	100	2.1	1.4	>98%
	353	n.r.	4.9	9.6	100	2.2	1.4	>98%
c-di-UMP	273	n.r.	4.9	9.7	100	1.7	1.2	>98%
	293	n.r.	4.9	9.7	100	1.9	1.4	>98%
	313	n.r.	5.0	9.6	100	2.0	1.4	>98%
	333	n.r.	5.0	9.5	100	2.1	1.6	>98%
	353	n.r.	5.0	9.4	100	2.3	1.6	>98%

n.r. Coupling constants could not be resolved due to limited resolution of spectra.
[a] Coupling constants could not be determined due to spectral overlap.
[b] Coupling constants that could not be resolved due to resolution of spectra were considered to be 0.7 Hz in the calculation.

Table S2. Transient NOEs for CDNs.

CDN	signal inverted	NOE [a]
c-di-GMP	H8<br>H1’	H1’ (0.3), H2’ (0.1), H3’ (0.6)<br>H8 (0.3)
c-di-AMP	H8<br>H1’	H1’ (0.6), H2’ (0.2), H3’ (2.1)<br>H8 (0.2)
c-GAMP	GH8<br>GH1’<br>AH8<br>AH1’	GH1’ (0.7), GH2’ (0.3), GH3’ (1.8)<br>GH8 (0.8)<br>AH1’ (0.7), AH2’ (0.3), AH3’ (1.0)<br>AH8 (0.8)
c-di-CMP	H6<br>H1’	H1’ (0.0), H2’ (0.1), H3’ (0.6)<br>H6 (0.0)
c-di-UMP	H6<br>H1’	H1’ (0.1), H2’ (0.1), H3’ (0.7)<br>H6 (0.1)

[a] Integral values were normalized to the irradiated peak, which was given a value of 100.

Table S3. $^{3}J_{H\text{-}P}$, $^{4}J_{H\text{-}P}$ and $^{3}J_{C\text{-}P}$ coupling constants (Hz) defining β and ε backbone torsion angles at 293K.

CDN	$J_{H3'\text{-}P3'}$	$J_{C2'\text{-}P3'}$	$J_{C4'\text{-}P3'}$	$J_{H5'\text{-}P5'}$	$J_{H5''\text{-}P5'}$	$J_{H4'\text{-}P5'}$	$J_{C4'\text{-}P5'}$
c-di-GMP	7.3	<1	9.0	<0.7	4.7	2.8	13.1
c-di-AMP	7.6	n.r.	10.0	n.r.	4.1	1.4	10.0
c-GAMP (Guanine moiety)	7.4	<1	10.5	<0.7	4.6	3.4	10.5
c-GAMP (Adenine moiety)	8.2	<1	11.9	<0.7	5.0	3.4	11.9
c-di-CMP	8.4	<1	10.7	0.5	3.3	3.2	10.7
c-di-UMP	8.6	<1	10.6	0.7	3.4	3.5	10.6

n.r. Coupling constants could not be resolved.

Synthesis of cyclic di-nucleotides

All solvents and reagents were purchased from commercial sources and used without further purification. Reaction was monitored by TLC on silica gel GF_{254} with detection under UV light. NMR spectra were recorded on Bruker AVANCE 400M instrument at 298K. Chemical shifts are relative to TMS (0.00). The following abbreviations were used to explain the multiplicities: s (singlet), d (doublet), t (triplet), q (quartet), m (multiplet). The number of protons (n) for a given resonance was indicated as nH. HRMS (MALTI-TOF) were obtained from Varian 7.0T FTMS. MS spectra were obtained Bruker Autoflex III TOF/TOF200 (MALTI-TOF) and LCQ-Advantage (ESI). UPLC-HRMS spectra were recorded on Waters Xevo G2-XS Q-TOF. HPLC was achieved using Agilent 1100 and semi-preparative column using an ASB C18 column (10 (diameter) x 250 (height) mm). 1M TEAB buffer was prepared by bubbling CO_2 through a 1M Et_3N solution in H_2O until pH 8.5. 1M TEAA buffer was prepared by adding CH_3COOH to Et_3N in water until pH 7.0 and then filtering through 0.45 um membrane filter. Both TEAA and TEAB buffer were kept in 4℃ fridge.

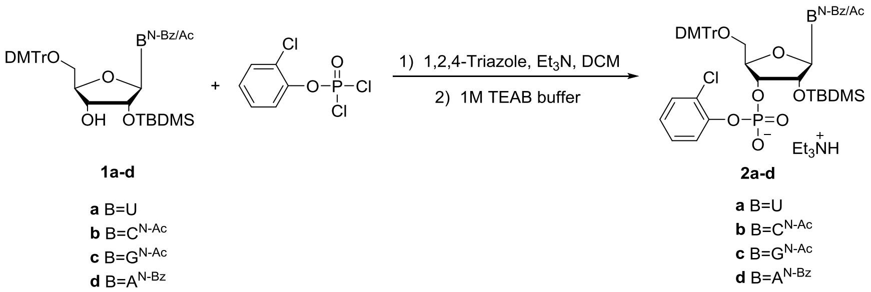

In a 250 ml two-necked flask, *1H*-1,2,4-triazole (50.6 mmol) and Et_3N (50.6 mmol) was dissolved in 20 ml dichloromethane under Ar atmosphere. 2-Chlorophenylphosphoryl Dichloride (20.3 mmol), dissolved in 10 ml dichloromethane, was added into the mixture slowly under 0 ℃ in 30 min. keeping stirring for another hour. A solution of **1a~d** (12.7 mmol) in 20 ml dichloromethane added dropwise into the mixture in 30 min under -10 ℃ and then the temperature was increased to 0 ℃. Two hours later 40 ml 1M TEAB was added into the flak and stirred for another half of an hour. The organic phase was separated and washed with 1M TEAB buffer and water. Dried over anhydrous sodium sulfate and then filtered, concentrated to obtain compound **2a~h** as white solid.

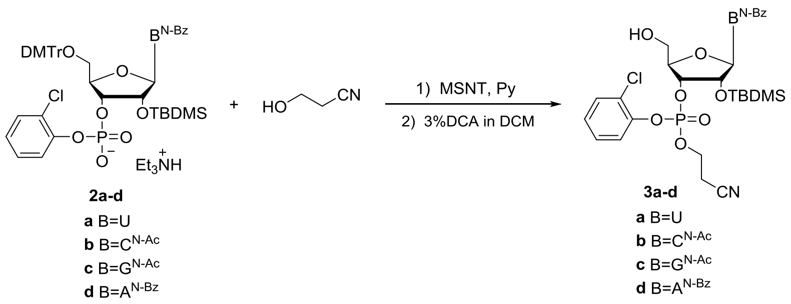

A mixture of **2a~d** (9.67 mmol), 1-(mesitylene-2-sulfonyl)-3-nitro-1,2,4-triazole (MSNT, 29.02 mmol) and 3-hydroxypropionitrile (14.5 mmol) in 40 ml anhydrous pyridine was stirred under room temperature in a 250 ml flask. Several drop of water was added into the flask to stop the reaction two

hours later. Afterwards, solvent was removed under reduced pressure and redissolved with 50 ml dichloromethane again. Several oxalic acid aqueous solutions were added into the mixture. The organic phase was separated and the water phase was extracted with dichloromethane. Combined the organic phase and washed with water twice (2 x 50 ml). Dried over anhydrous sodium sulfate and filtered. Solvent was removed under reduced pressure and the residue was redissolved in 30 ml dichloromethane and stirred under ice-water bath. A solution of 6% dichloroaceticacid in dichloromethane was added into the mixture and continuously stirred for 5 min. afterwards several drop of methanol was added. Saturated solution of sodium bicarbonate was added to regulate the pH to neutral. Organic phase was separated and the water phase was extracted with dichloromethane. The organic phase was combined and washed with sodium bicarbonate saturated solution and water. And then it was dried over anhydrous sodium sulfate, filtered and concentrated. The residual compound was purified by chromatography on silica gel (DCM: Methanol = 100:1~100:30) to give compounds **3a~d** as white solid. (Yields 75~85 % within two steps)

3a B=U: 1H NMR (400 MHz, $CDCl_3$) δ 9.44 (d, J = 52.9 Hz, 1H), 7.69 (dd, J = 68.3, 8.1 Hz, 1H), 7.42 (d, J = 7.8 Hz, 2H), 7.28 – 7.21 (m, 1H), 7.16 (t, J = 7.7 Hz, 1H), 6.02 (dd, J = 55.0, 5.7 Hz, 1H), 5.71 (ddd, J = 20.7, 8.1, 1.9 Hz, 1H), 5.36 – 5.17 (m, 1H), 4.61 – 4.50 (m, 1H), 4.49 – 4.35 (m, 2H), 4.13 (d, J = 2.9 Hz, 1H), 3.94 (t, J = 15.4 Hz, 1H), 3.78 (d, J = 11.4 Hz, 1H), 3.07 (s, 1H), 2.81 (t, J = 6.0 Hz, 2H), 0.92 (d, J = 7.0 Hz, 9H); 13C NMR (101 MHz, $CDCl_3$) δ 162.31, 149.75, 149.46, 145.05, 144.99, 140.86, 140.29, 129.80, 127.30, 127.19, 125.73, 125.57, 124.25, 124.18, 120.51, 120.31, 120.29, 115.53, 101.91, 87.70, 85.41, 84.99, 69.88, 69.82, 62.39, 62.33, 60.51, 60.31, 24.66, 24.64, 18.59, 18.51, 17.07, -0.00, -5.77, -6.05, -6.13. MALDI-TOF-HRMS calcd for C24H33ClN3O9PSi: 601.1412. found $[M+Na]^+$ 624.1310.

3b B=$C^{N\text{-}Ac}$: 1H NMR (400 MHz, $CDCl_3$) δ 10.02 (d, J = 10.1 Hz, 1H), 8.30 (dd, J = 14.8, 7.5 Hz, 1H), 7.32 (dt, J = 14.5, 8.1 Hz, 3H), 7.17 (t, J = 7.0 Hz, 1H), 7.06 (dd, J = 14.9, 7.4 Hz, 1H), 5.76 – 5.59 (m, 1H), 5.15 – 4.99 (m, 1H), 4.62 (d, J = 28.6 Hz, 1H), 4.50 – 4.28 (m, 3H), 4.22 (d, J = 5.2 Hz, 1H), 3.78 (dt, J = 16.0, 13.4 Hz, 2H), 2.72 (d, J = 5.2 Hz, 2H), 2.12 (s, 3H), 0.76 (d, J = 8.6 Hz, 9H), -0.01 (dd, J = 22.1, 19.3 Hz, 6H); 13C NMR (101 MHz, $CDCl_3$) δ 171.53, 163.16, 163.07, 155.76, 155.54, 145.99, 145.93, 145.72, 145.57, 130.93, 130.86, 128.35, 128.20, 126.85, 126.79, 125.63, 125.56, 125.26, 125.19, 121.61, 121.54, 116.42, 116.22, 97.25, 97.01, 91.89, 91.53, 83.04, 82.68, 74.38, 63.67, 63.62, 63.42, 63.37, 60.20, 59.83, 53.50, 25.67, 25.62, 24.85, 19.70, 19.60, 19.52, 18.01, -4.70, -4.81, -5.13, -5.24. MALDI-TOF-HRMS calcd for C26H36ClN4O9PSi: 642.1678. found $[M+Na]^+$ 665.1575.

3c B=$G^{N\text{-}Ac}$: 1H NMR (400 MHz, $CDCl_3$) δ 12.32 (s, 1H), 9.91 (s, 1H), 7.98 (s, 1H), 7.47 (dd, J = 7.3, 2.4 Hz, 2H), 7.31 (dd, J = 11.0, 4.5 Hz, 1H), 7.20 (t, J = 7.7 Hz, 1H), 5.80 (d, J = 6.9 Hz, 1H), 5.30 (d, J = 8.1 Hz, 2H), 5.09 (t, J = 5.0 Hz, 1H), 4.51 (ddd, J = 18.8, 10.4, 3.8 Hz, 2H), 4.30 (s, 1H), 3.98 (dd, J = 12.6, 3.1 Hz, 1H), 3.73 (d, J = 10.9 Hz, 1H), 3.00 – 2.80 (m, 2H), 2.32 (s, 3H), 0.74 (s, 9H), -0.10 (s, 3H), -0.23 (s, 3H); 13C NMR (101 MHz, $CDCl_3$) δ 173.41, 155.59, 148.08, 148.01, 146.02, 145.96, 139.53, 130.89, 128.24, 126.77, 125.42, 125.35, 121.90, 121.63, 116.46, 89.33, 84.11, 78.57, 73.41, 63.43, 63.38, 61.26, 53.53, 25.67, 25.38, 24.26, 19.71, 19.64, 17.80, -5.20, -5.42. MALDI-TOF-HRMS calcd for C27H36ClN6O9PSi: 682.1739. found $[M+Na]^+$ 705.1638.

3d B=A^{N-Bz}: **3d** 1H NMR (400 MHz, $CDCl_3$) δ 9.13 (s, 1H), 8.82 (d, J = 6.4 Hz, 1H), 8.09 – 8.01 (m, 3H), 7.62 (t, J = 7.4 Hz, 1H), 7.57 – 7.46 (m, 3H), 7.33 – 7.28 (m, 1H), 7.19 (t, J = 7.8 Hz, 1H), 6.07 (d, J = 11.5 Hz, 1H), 5.92 (d, J = 7.8 Hz, 1H), 5.83 (d, J = 7.4 Hz, 1H), 5.28 – 5.22 (m, 1H), 5.19 – 5.11 (m, 1H), 4.47 (dt, J = 14.5, 6.1 Hz, 1H), 4.37 (d, J = 5.1 Hz, 1H), 3.95 (dd, J = 24.8, 13.1 Hz, 1H), 3.75 (dt, J = 25.6, 9.8 Hz, 1H), 2.83 (t, J = 6.3 Hz, 2H), 1.78 (s, 2H), 0.76 (d, J = 28.4 Hz, 9H), -0.14 (d, J = 18.0 Hz, 3H), -0.39 (d, J = 6.0 Hz, 3H); 13C NMR (101 MHz, $CDCl_3$) δ 164.46, 152.46, 150.60, 150.35, 146.01, 143.09, 142.94, 133.45, 133.02, 130.95, 128.95, 128.28, 127.91, 126.76, 125.52, 124.25, 121.58, 116.05, 91.33, 90.53, 87.66, 86.34, 80.47, 80.41, 76.72, 74.41, 73.18, 73.13, 72.78, 63.29, 63.23, 63.17, 62.57, 25.76, 25.50, 25.44, 19.69, 19.62, 17.88, 17.80, -5.14, -5.28, -5.43, -5.71. MALDI-TOF-HRMS calcd for C32H38ClN6O8PSi: 728.1947. found $[M+Na]^+$ 751.1842.

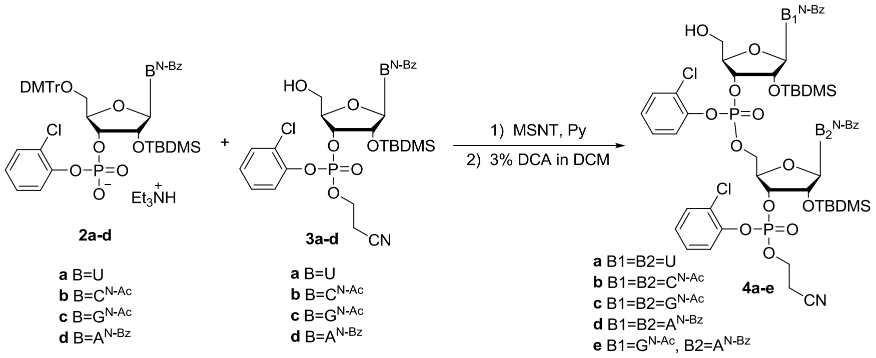

A mixture of **2a~d** (2.22 mmol), **3a~d** (1.85 mmol.), and MSNT (3.70 mmol.) in 20 ml anhydrous pyridine was stirred under room temperature in a 100 ml flask. Several drop of water was added into the flask to stop the reaction when the TLC indicated the reaction was completed. Afterwards, solvent was removed under reduced pressure and resolved with 20 ml dichloromethane again. Several oxalic acid aqueous solutions were added into the mixture. The organic phase was separated and the water phase was extracted with dichloromethane. Combined the organic phase and washed with water twice (2 x 50 ml). Dried over anhydrous sodium sulfate and filtered. Solvent was removed under reduced pressure and the residue was redissolved with 15 ml dichloromethane and stirred under ice-water bath. A solution of 6% dichloroaceticacid in dichloromethane (15 ml) was added into the mixture and continuously stirred for 5 min. afterwards several drop of methanol was added. Saturated solution of sodium bicarbonate was added to regulate the pH to neutral. Organic phase was separated and the water phase was extracted with dichloromethane. The organic phase was combined and washed with sodium bicarbonate saturated solution and water, and further dried over anhydrous sodium sulfate, filtered and concentrated. The residual compound was purified by chromatography on silica gel (DCM: Methanol = 100:1~100:30) to give compounds **4a~e** as white solid. (Yields 75~85 % within two steps)

4a B1=B2=U: 1H NMR (400 MHz, DMSO) δ 11.77 (s, 1H), 11.18 (s, 1H), 7.92 (d, J = 8.2 Hz, 1H), 7.61 – 7.46 (m, 3H), 7.39 – 7.30 (m, 2H), 7.30 – 7.19 (m, 2H), 7.18 – 7.07 (m, 1H), 6.91 (td, J = 7.8, 1.4 Hz, 1H), 6.12 (d, J = 6.0 Hz, 1H), 5.93 (d, J = 7.5 Hz, 1H), 5.65 (d, J = 8.2 Hz, 1H), 5.57 (s, 1H), 5.44 (d, J = 8.1 Hz, 1H), 5.09 (dt, J = 8.5, 5.7 Hz, 1H), 4.96 (dd, J = 12.6, 9.6 Hz, 1H), 4.57 (dt,

J = 13.6, 6.8 Hz, 2H), 4.47 – 4.31 (m, 3H), 4.15 (dd, J = 11.2, 5.7 Hz, 1H), 4.00 (dd, J = 13.7, 4.6 Hz, 2H), 3.75 – 3.56 (m, 2H), 2.55 – 2.45 (m, 2H), 0.84 (d, J = 1.8 Hz, 18H), 0.06 (dd, J = 13.7, 7.6 Hz, 12H); 13C NMR (101 MHz, $CDCl_3$) δ 164.02, 151.05, 150.83, 146.38, 146.19, 141.65, 140.86, 140.41, 130.97, 128.41, 126.74, 125.43, 121.65, 116.93, 103.21, 102.97, 88.67, 87.71, 86.46, 86.02, 82.79, 79.55, 71.27, 71.04, 70.09, 67.03, 63.86, 61.37, 61.08, 25.82, 19.62, 18.25, 18.15, -4.60, -4.89, -5.04. MALDI-TOF-HRMS calcd for C45H61Cl2N5O17P2Si2: 1131.2453. found $[M+Na]^+$ 1154.2348.

4b B1=B2=$C^{N\text{-}Ac}$: 1H NMR (400 MHz, $CDCl_3$) δ 9.85 (d, J = 44.8 Hz, 2H), 8.32 (d, J = 7.0 Hz, 1H), 8.19 (d, J = 6.7 Hz, 1H), 7.49 (d, J = 6.5 Hz, 1H), 7.40 (dd, J = 12.0, 6.4 Hz, 3H), 7.22 (t, J = 7.6 Hz, 1H), 7.12 (t, J = 7.8 Hz, 1H), 6.05 (d, J = 3.6 Hz, 1H), 5.71 (d, J = 2.8 Hz, 1H), 5.46 – 5.23 (m, 1H), 4.65 – 4.23 (m, 5H), 4.12 (d, J = 17.8 Hz, 2H), 4.06 – 3.83 (m, 4H), 3.77 (d, J = 16.6 Hz, 2H), 3.58 (d, J = 3.9 Hz, 1H), 2.84 (dd, J = 11.4, 5.7 Hz, 2H), 2.21 (d, J = 14.4 Hz, 6H), 0.96 – 0.83 (m, 18H), 0.16 – 0.06 (m, 12H); 13C NMR (101 MHz, $CDCl_3$) δ 170.26, 161.90, 154.80, 154.53, 145.64, 145.27, 129.71, 127.03, 125.49, 124.54, 120.67, 115.68, 96.36, 96.20, 93.25, 89.35, 84.88, 84.69, 78.47, 76.35, 76.03, 75.71, 73.55, 69.80, 68.80, 62.50, 60.03, 59.51, 24.70, 24.64, 23.86, 18.56, 18.49, 17.06, 17.03, -0.00, -5.79, -5.83, -6.20. MALDI-TOF-HRMS calcd for C49H67Cl2N7O17P2Si2: 1213.2984. found $[M+Na]^+$ 1236.2879.

4c B1=B2=$G^{N\text{-}Ac}$: 1H NMR (400 MHz, $CDCl_3$) δ 12.22 (d, J = 29.2 Hz, 1H), 11.90 (s, 1H), 10.20 (d, J = 22.0 Hz, 1H), 9.75 (d, J = 59.8 Hz, 1H), 8.16 (d, J = 46.3 Hz, 1H), 7.75 (s, 1H), 7.53 – 7.41 (m, 2H), 7.39 – 7.29 (m, 2H), 7.26 – 7.16 (m, 1H), 7.05 (dd, J = 15.6, 7.6 Hz, 2H), 6.73 (t, J = 7.7 Hz, 1H), 6.06 – 5.96 (m, 1H), 5.79 (dd, J = 17.3, 8.1 Hz, 1H), 5.64 (d, J = 31.3 Hz, 1H), 5.46 – 5.34 (m, 1H), 5.26 – 5.17 (m, 1H), 5.12 (d, J = 3.4 Hz, 1H), 4.84 – 4.44 (m, 6H), 4.09 (dd, J = 20.4, 11.8 Hz, 1H), 3.92 (dd, J = 26.7, 12.2 Hz, 1H), 2.95 – 2.78 (m, 2H), 2.25 (d, J = 11.1 Hz, 3H), 1.96 (s, 3H), 0.69 (dd, J = 10.8, 8.1 Hz, 18H), -0.08 – -0.33 (m, 12H); 13C NMR (101 MHz, $CDCl_3$) δ 173.09, 172.99, 172.86, 155.65, 155.57, 148.50, 148.35, 148.16, 148.08, 147.85, 147.28, 145.87, 145.81, 145.19, 145.12, 140.60, 138.96, 130.98, 130.47, 128.47, 128.27, 127.46, 127.23, 127.13, 126.94, 126.06, 126.00, 125.43, 125.36, 122.83, 122.65, 121.84, 121.59, 121.31, 116.64, 116.44, 89.17, 88.94, 87.92, 84.77, 81.60, 73.81, 70.63, 68.39, 64.09, 63.82, 63.76, 61.84, 25.37, 25.33, 25.30, 24.24, 23.62, 19.82, 19.74, 17.84, 17.78, -4.96, -4.99, -5.08, -5.18, -5.42, -5.46, -5.51, -5.62. MALDI-TOF-HRMS calcd for C51H67Cl2N11O17P2Si2: 1293.3107. found $[M+Na]^+$ 1316.3006.

4d B1=B2=$A^{N\text{-}Bz}$: 1H NMR (400 MHz, $CDCl_3$) δ 9.22 (s, 1H), 8.94 – 8.75 (m, 2H), 8.29 (d, J = 9.4 Hz, 2H), 8.03 (t, J = 8.5 Hz, 4H), 7.69 – 7.38 (m, 9H), 7.29 (s, 1H), 7.24 – 7.10 (m, 2H), 6.25 – 5.82 (m, 3H), 5.29 (dd, J = 51.0, 17.8 Hz, 4H), 5.01 – 4.24 (m, 6H), 3.88 (dd, J = 38.2, 12.1 Hz, 2H), 2.83 (s, 2H), 1.01 – 0.57 (m, 18H), -0.11 (ddd, J = 99.2, 53.9, 47.4 Hz, 12H). 13C NMR (101 MHz, $CDCl_3$) δ 164.80, 163.57, 152.80, 152.18, 151.49, 150.34, 149.82, 146.13, 145.44, 143.75, 143.54, 141.99, 133.36, 133.04, 132.90, 130.94, 130.82, 128.89, 128.81, 128.31, 128.15, 127.94, 126.84, 126.54, 125.28, 124.13, 123.45, 121.59, 121.45, 116.29, 90.41, 90.26, 89.59, 88.89, 86.02, 82.52, 81.11, 79.87, 79.64, 75.04, 73.77, 73.20, 70.39, 67.43, 63.45, 62.46, 53.54, 25.60, 25.42, 25.40, 19.67, 17.95, 17.87, 17.79, -4.81, -5.01, -5.19, -5.70. MALDI-TOF-HRMS calcd for C61H71Cl2N11O15P2Si2: 1385.3522. found $[M+Na]^+$ 1408.3420.

4e B1=G$^{N\text{-}Ac}$, B2=A$^{N\text{-}Bz}$: 1H NMR (400 MHz, $CDCl_3$) δ 12.36 (m, 1H), 11.16 – 10.48 (m, 1H), 9.02 – 8.83 (m, 1H), 8.22 (dd, J = 28.9, 7.2 Hz, 2H), 8.02 (d, J = 6.6 Hz, 2H), 7.60 (d, J = 6.3 Hz, 1H), 7.50 (dd, J = 15.9, 8.2 Hz, 2H), 7.46 – 7.35 (m, 2H), 7.30 (d, J = 7.8 Hz, 1H), 7.19 (ddd, J = 27.5, 17.2, 9.6 Hz, 3H), 6.07 (dd, J = 31.5, 4.7 Hz, 1H), 5.84 (dd, J = 11.4, 7.5 Hz, 1H), 5.44 (d, J = 23.8 Hz, 1H), 5.28 (d, J = 27.8 Hz, 2H), 5.01 (s, 1H), 4.85 – 4.61 (m, 2H), 4.57 – 4.38 (m, 2H), 4.24 (d, J = 28.2 Hz, 1H), 3.95 (d, J = 10.9 Hz, 1H), 3.65 (d, J = 11.9 Hz, 1H), 2.84 (s, 1H), 2.26 (d, J = 3.9 Hz, 3H), 0.93 – 0.67 (m, 18H), 0.07 – -0.33 (m, 12H).; 13C NMR (101 MHz, $CDCl_3$) δ 173.48, 173.41, 165.14, 155.69, 152.68, 151.37, 149.94, 149.81, 148.74, 148.55, 148.31, 148.14, 145.99, 142.94, 138.98, 133.17, 133.00, 130.97, 130.78, 130.70, 128.85, 128.42, 128.21, 127.94, 127.02, 126.91, 126.59, 126.44, 125.33, 123.95, 121.40, 121.18, 116.29, 116.19, 99.99, 90.29, 89.23, 88.05, 84.87, 82.88, 81.25, 74.74, 74.42, 70.88, 67.41, 63.70, 61.63, 25.60, 25.43, 24.24, 24.16, 19.66, 17.96, 17.89, 17.86, -4.86, -4.99, -5.14, -5.21, -5.56. MALDI-TOF-HRMS calcd for C56H69Cl2N11O16P2Si2: 1339.3315. found $[M+Na]^+$ 1362.3212.

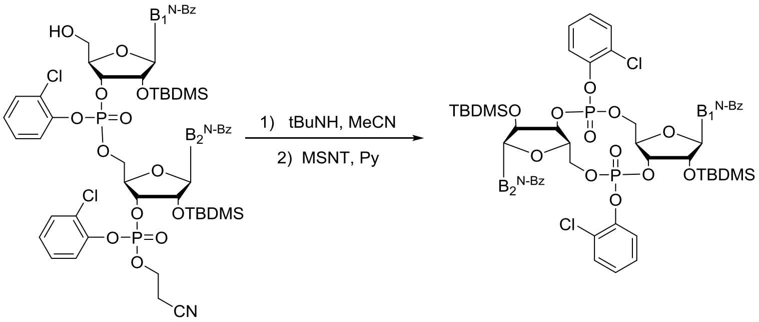

4a-e

a B1=B2=U
b B1=B2=C$^{N\text{-}Ac}$
c B1=B2=G$^{N\text{-}Ac}$
d B1=B2=A$^{N\text{-}Bz}$
e B1=G$^{N\text{-}Ac}$, B2=A$^{N\text{-}Bz}$

5a-e

a B1=B2=U
b B1=B2=C$^{N\text{-}Ac}$
c B1=B2=G$^{N\text{-}Ac}$
d B1=B2=A$^{N\text{-}Bz}$
e B1=G$^{N\text{-}Ac}$, B2=A$^{N\text{-}Bz}$

In a 100 ml flask, **4a-e** (0.6 mmol) was dissolved in 8 ml acetonitrile and 8 ml *tert*-butylamine and then stirred under room temperature for 20 min. Solvent was evaporated under reduced pressure. Acetonitrile was added to the residual compounds again and evaporated. 20 ml anhydrous pyridine was added after the residual was fully dried. MSNT (3.0 mmol) was added in batches. The mixture was stirred under room temperature for 6 hours. The reaction was stopped with addition of drops of water. The solvent was removed under reduced pressure and the residual was dissolved in 30 ml dichloromethane. Wash with acidic water, dried over anhydrous sodium sulfate, filtered and concentrated. Further purification with chromatography on silica gel (dichloromethane: methanol = 80:1 ~ 15:1) give the fully protected cyclic dinucleotides **5a-e** as white or yellow solid. (Yield 50~60 within two steps)

5a B1=B2=U: 1H NMR (400 MHz, $CDCl_3$) δ 10.62 (s, 2H), 7.44 (s, 2H), 7.31 (d, J = 6.5 Hz, 3H), 7.18 (dt, J = 14.3, 7.6 Hz, 4H), 7.06 (t, J = 7.5 Hz, 2H), 6.16 (s, 2H), 5.97 (s, 2H), 5.57 (s, 2H), 4.61 (dd, J = 11.3, 3.7 Hz, 4H), 4.49 (dd, J = 11.7, 2.9 Hz, 2H), 4.33 (s, 2H), 1.02 (s, 18H), 0.28 (d, J = 19.2 Hz, 12H); 13C NMR (101 MHz, $CDCl_3$) δ 165.04, 150.09, 145.98, 145.92, 131.03, 128.01, 126.51, 125.15, 125.07, 120.30, 103.87, 83.60, 83.51, 75.23, 70.96, 68.54, 53.57, 25.79, 25.56, 18.23, -4.48, -4.72. MALDI-TOF-HRMS calcd for C42H56Cl2N4O16P2Si2: 1060.2082. found $[M+Na]^+$

1083.1978.

5b B1=B2=C^{N-Ac}: 1H NMR (400 MHz, $CDCl_3$) δ 10.15 (d, J = 20.2 Hz, 2H), 8.07 (d, J = 7.6 Hz, 1H), 7.65 (t, J = 6.7 Hz, 1H), 7.58 (d, J = 8.1 Hz, 1H), 7.54 – 7.39 (m, 4H), 7.38 – 7.27 (m, 3H), 7.27 – 7.17 (m, 2H), 7.09 (d, J = 7.5 Hz, 1H), 6.96 (d, J = 7.5 Hz, 1H), 5.72 – 5.60 (m, 2H), 5.05 – 4.86 (m, 2H), 4.80 (d, J = 10.0 Hz, 1H), 4.72 – 4.43 (m, 7H), 2.33 – 2.23 (m, 6H), 0.95 (s, 11H), 0.87 (s, 7H), 0.20 (d, J = 6.6 Hz, 6H), 0.09 (d, J = 10.3 Hz, 6H); 13C NMR (101 MHz, $CDCl_3$) δ 171.87, 163.98, 155.31, 146.75, 146.31, 144.73, 144.22, 132.41, 132.04, 131.76, 129.58, 129.32, 129.09, 127.98, 127.86, 127.47, 125.67, 121.76, 121.43, 120.92, 97.58, 97.36, 96.95, 93.38, 78.76, 75.44, 74.87, 74.53, 66.35, 65.34, 26.42, 25.66, 18.80, 18.73, -3.98, -4.56, -4.64. MALDI-TOF-HRMS calcd for C46H62Cl2N6O16P2Si2: 1142.2613. found $[M+Na]^+$ 1165.2512.

5c B1=B2=G^{N-Ac}: 1H NMR (400 MHz, $CDCl_3$) δ 12.02 (s, 2H), 10.26 (s, 2H), 7.68 (s, 2H), 7.43 (d, J = 7.7 Hz, 2H), 7.31 (d, J = 7.1 Hz, 2H), 7.23 – 7.13 (m, 4H), 5.84 (dd, J = 9.5, 5.1 Hz, 2H), 5.78 (d, J = 2.6 Hz, 2H), 5.28 (s, 2H), 5.17 (d, J = 9.8 Hz, 2H), 4.64 – 4.52 (m, 2H), 4.42 – 4.28 (m, 2H), 1.93 (s, 6H), 0.80 (s, 18H), -0.01 (s, 6H), -0.08 (s, 6H); 13C NMR (101 MHz, $CDCl_3$) δ 172.59, 155.36, 147.64, 147.01, 146.03, 145.96, 139.05, 131.01, 128.23, 127.23, 125.50, 125.43, 122.83, 121.34, 92.19 , 73.71, 65.72, 25.63, 25.51, 23.57, 17.92, -4.98, -5.00. MALDI-TOF-HRMS calcd for C48H62Cl2N10O16P2Si2: 1222.2736. found $[M+Na]^+$ 1245.2632.

5d B1=B2=A^{N-Bz}: 1H NMR (400 MHz, $CDCl_3$) δ 9.40 (s, 2H), 8.74 (s, 2H), 8.06 (s, 2H), 8.00 (d, J = 7.4 Hz, 5H), 7.55 (d, J = 7.3 Hz, 2H), 7.46 (t, J = 7.6 Hz, 9H), 7.27 (t, J = 7.7 Hz, 2H), 7.18 (t, J = 7.7 Hz, 2H), 5.92 (d, J = 7.5 Hz, 2H), 5.70 (s, 2H), 5.46 – 5.35 (m, 2H), 5.04 (q, J = 10.7 Hz, 2H), 4.59 (dd, J = 10.7, 3.3 Hz, 2H), 4.21 (t, J = 7.8 Hz, 2H), 0.67 (s, 18H), -0.00 (s, 6H), -0.27 (s, 6H); 13C NMR (101 MHz, $CDCl_3$) δ 165.00, 152.63, 151.34, 150.09, 146.22, 146.15, 143.18, 133.56, 132.75, 130.86, 128.72, 128.09, 127.95, 126.80, 125.70, 125.64, 124.19, 121.94, 89.52, 81.81, 70.55, 65.98, 53.55, 25.39, 17.71, -5.09, -5.71. MALDI-TOF-HRMS calcd for C58H66Cl2N10O14P2Si2: 1314.3151. found $[M+Na]^+$ 1337.3048.

5e B1=G^{N-Ac}, B2=A^{N-Bz}: 1H NMR (400 MHz, $CDCl_3$) δ 12.04 (s, 1H), 10.29 (s, 1H), 8.76 (s, 1H), 8.13 – 7.99 (m, 3H), 7.71 – 7.59 (m, 2H), 7.53 (dd, J = 13.8, 7.4 Hz, 3H), 7.46 (d, J = 7.8 Hz, 2H), 7.31 (t, J = 7.2 Hz, 2H), 7.25 – 7.15 (m, 3H), 5.94 (d, J = 4.3 Hz, 1H), 5.78 (d, J = 3.5 Hz, 1H), 5.75 – 5.66 (m, 1H), 5.53 – 5.45 (m, 2H), 5.45 – 5.37 (m, 1H), 5.16 (q, J = 10.2 Hz, 1H), 4.83 (dd, J = 19.7, 9.6 Hz, 1H), 4.62 (dd, J = 9.0, 4.8 Hz, 2H), 4.34 (td, J = 10.5, 6.9 Hz, 2H), 1.90 (s, 3H), 0.79 (d, J = 16.0 Hz, 18H), 0.01 (s, 6H), -0.11 (d, J = 26.5 Hz, 6H); 13C NMR (101 MHz, $CDCl_3$) δ 172.85, 164.82, 155.51, 152.71, 151.23, 150.08, 147.80, 147.29, 146.11, 146.05, 146.01, 142.65, 139.23, 133.51, 132.86, 130.96, 130.89, 128.84, 128.22, 127.98, 127.22, 126.80, 125.68, 125.61, 125.38, 125.30, 124.04, 122.79, 121.65, 121.56, 91.91, 89.79, 80.98, 80.70, 80.63, 73.12, 71.76, 71.70, 66.00, 53.53, 25.50, 25.45, 23.69, 17.89, 17.82, -5.06, -5.13, -5.34. MALDI-TOF-HRMS calcd for C53H64Cl2N10O15P2Si2: 1268.2943. found $[M+Na]^+$ 1291.2842.

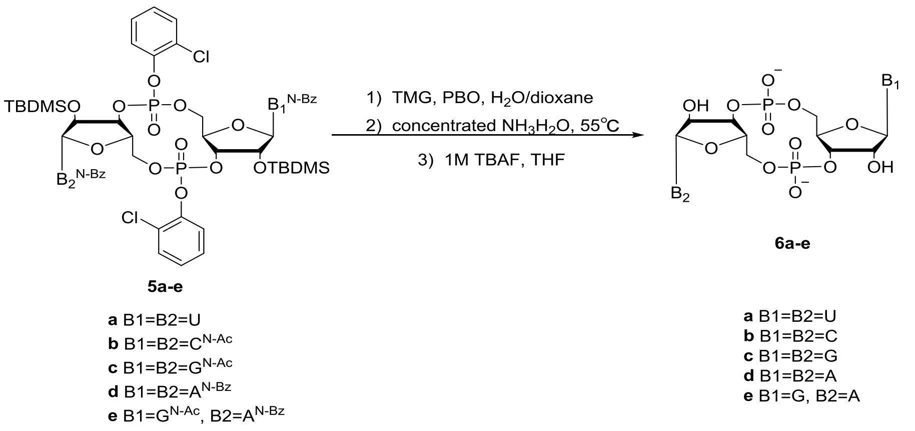

Fully protected cyclic dinucleotide **5a-e** (100 mg) was dissolve in 5 ml 1,4-dioxane/water (1:1, v/v) and then pyridine-2-carboxaldoxime and *N1,N1,N3,N3*-tetramethylguanidine was added. The mixture was stirred under room temperature for 24 hours. Afterward, the solvent was removed under reduced pressure. 5 ml concentrated ammonia was added. The system was stirred under 55 ℃ overnight and then removed the solvent. Fully dried 3 ml 1M tetrabutylammonium fluoride (TBAF, in THF) was added and stirred for 24 hours under room temperature. Then the solvent was evaporated and dissolved with deionized water. The water phase was wash with chloroform then recrystallized in acetone. The final deprotected cyclic di-nucleotides **6a-e** as triethylammonium salts were purified with reverse phase HPLC (Yield 15-25%).

6a: c-di-UMP, B1=B2=U. MALDI-TOF-HRMS calcd for C18H22N4O16P2: 612.0506, found $[M+Na]^+$ 635.0402.

6b: c-di-CMP, B1=B2=C. ESI-TOF-HRMS calcd for C18H24N6O14P2: 610.0826, found $[M-H]^-$ 609.0791.

6c: c-di-GMP, B1=B2=G. ESI-TOF-HRMS calcd for C20H24N10O14P2: 690.0949, found $[M-H]^-$ 689.0759, $[M-2H]^{2-}$ 344.0319.

6d: c-di-AMP, B1=B2=A. MALDI-TOF-HRMS calcd for C20H24N10O12P2: 658.1050, found $[M-H]^-$ 657.0905.

6e: c-GAMP, B1=G, B2=A. ESI-TOF-HRMS calcd for C20H24N10O13P2: 674.1000, found $[M-H]^-$ 673.0930.

RP-HPLC methodology

Time (min)	%A water	% B MeCN	% C 1M TEAA buffer	Flow (ml/min)
2	88	2	10	3
30	70	20	10	3
32	70	20	10	3
35	0	100	0	3
40	0	100	0	4
42	88	2	10	3

NMR spectroscopy for the characterization of the compound during the synthesis

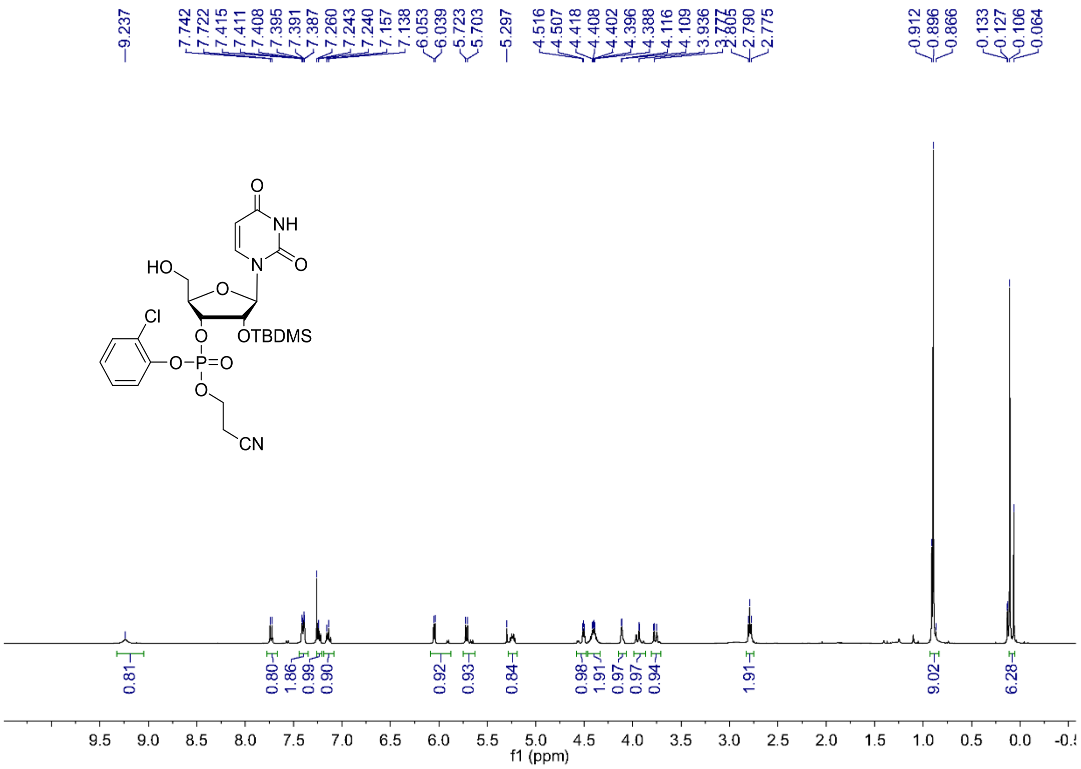

Figure S27. ^{1}H-NMR in $CDCl_3$ of **3a**

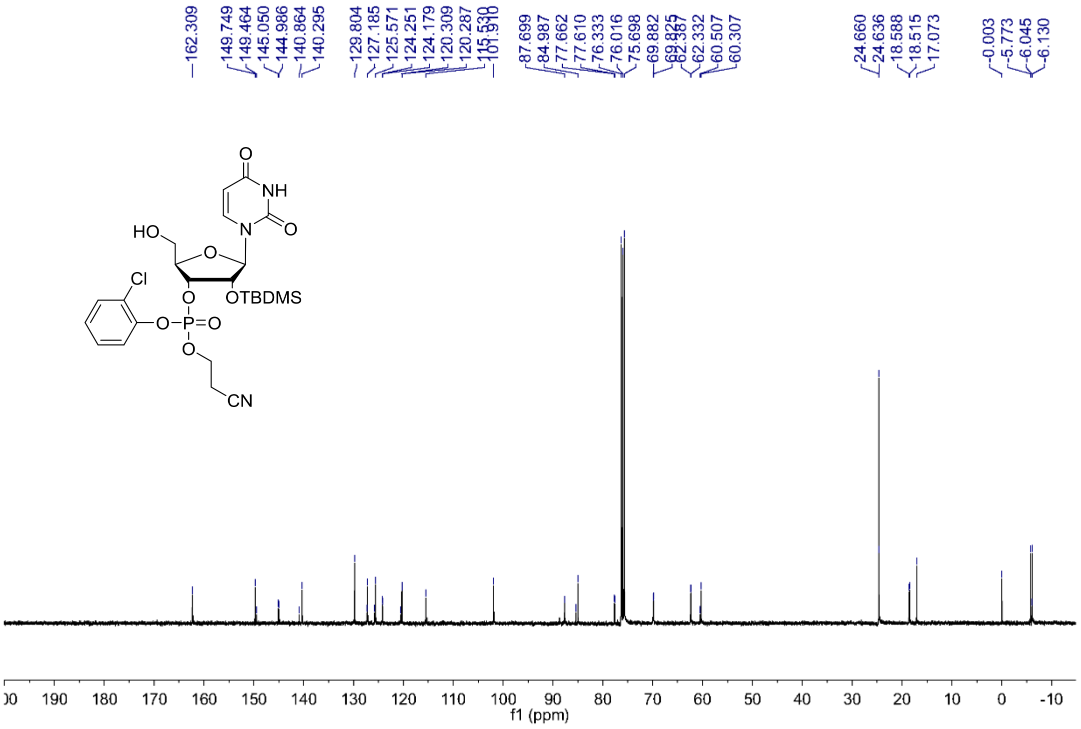

Figure S28. ^{13}C NMR in $CDCl_3$ of **3a**

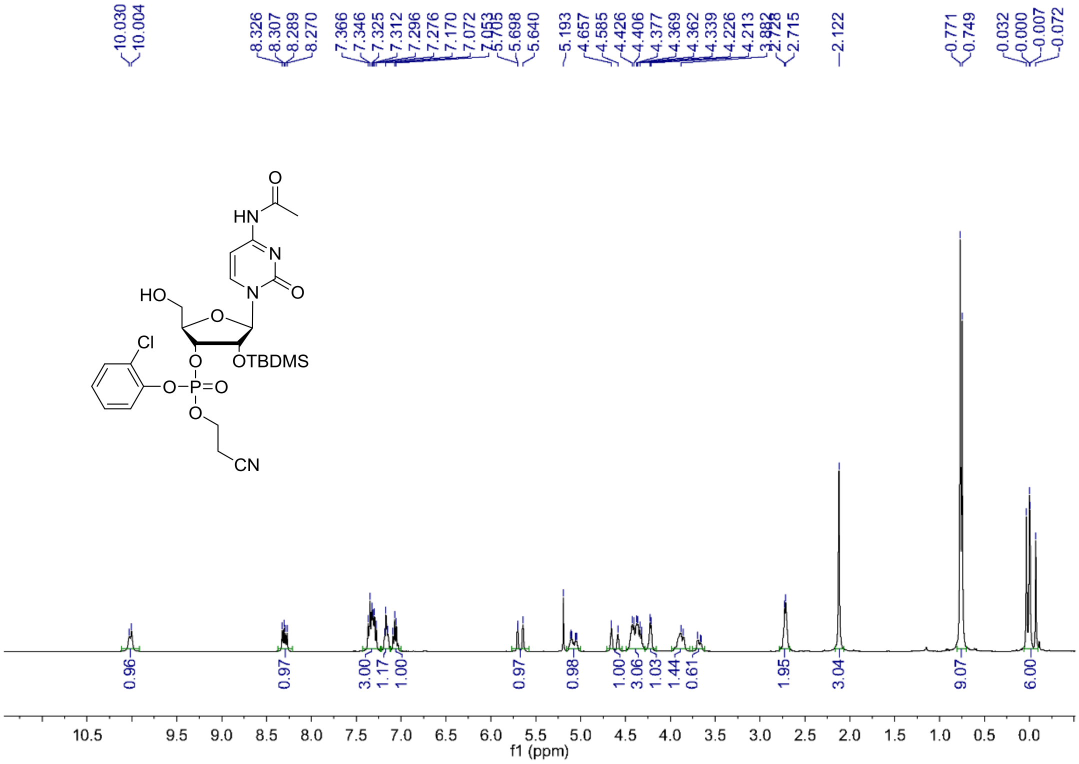

Figure S29. ^{1}H-NMR in $CDCl_3$ of **3b**

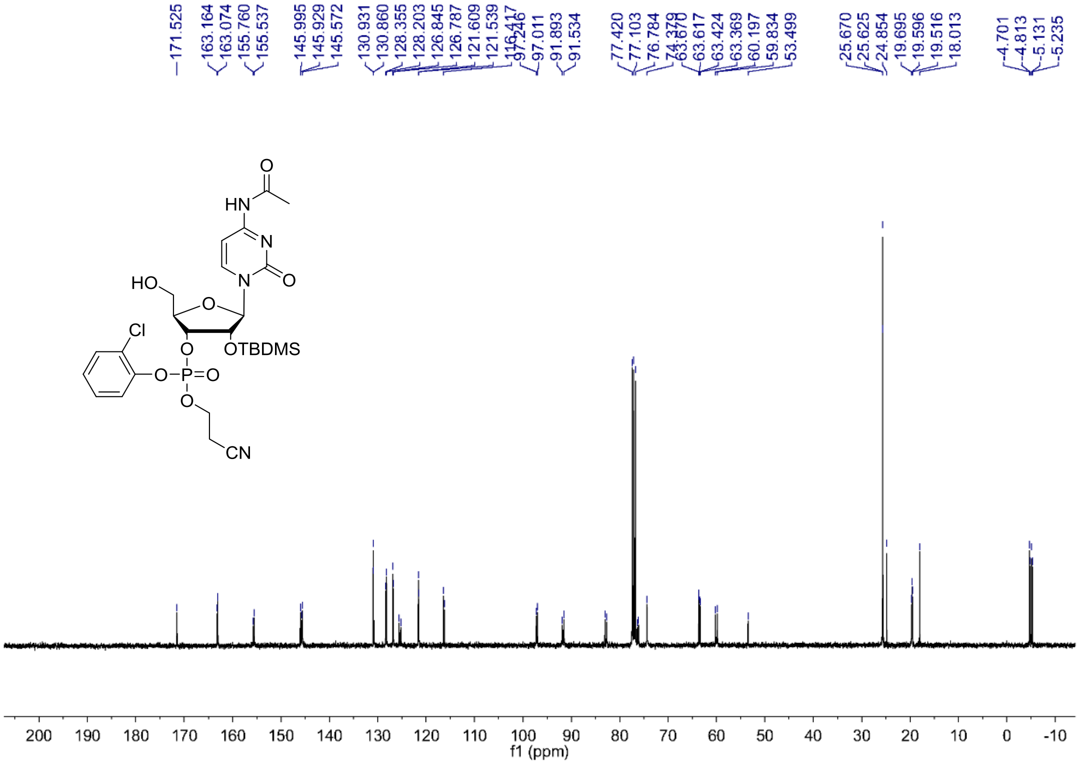

Figure S30. ^{13}C NMR in $CDCl_3$ of **3b**

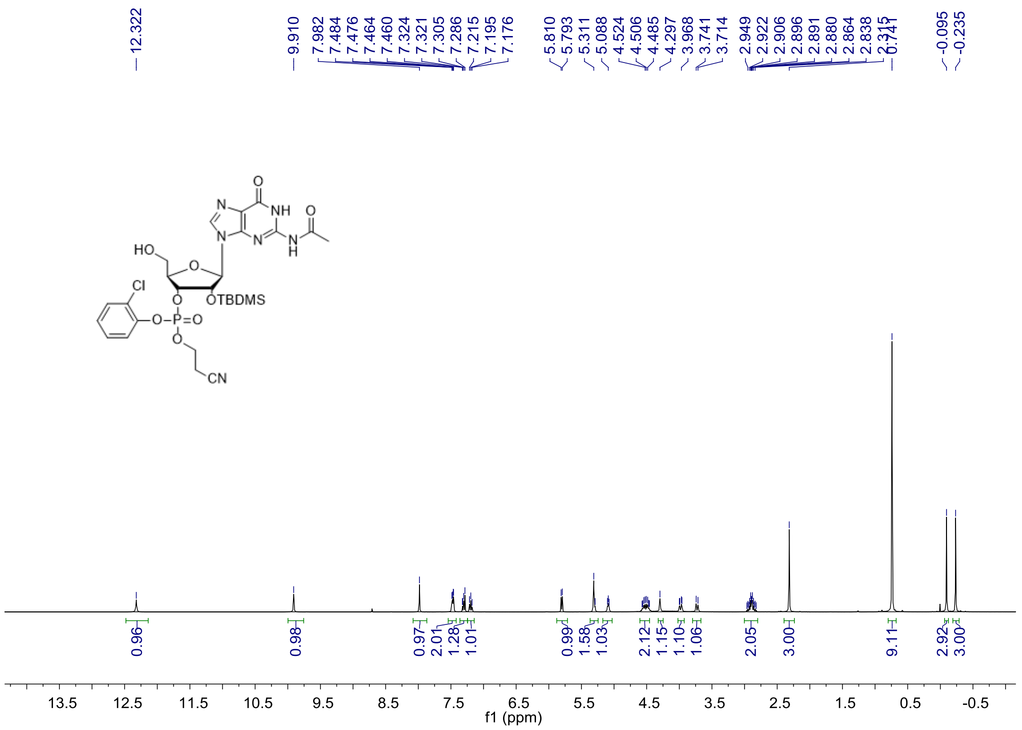

Figure S31. ^{1}H NMR in $CDCl_3$ of **3c**

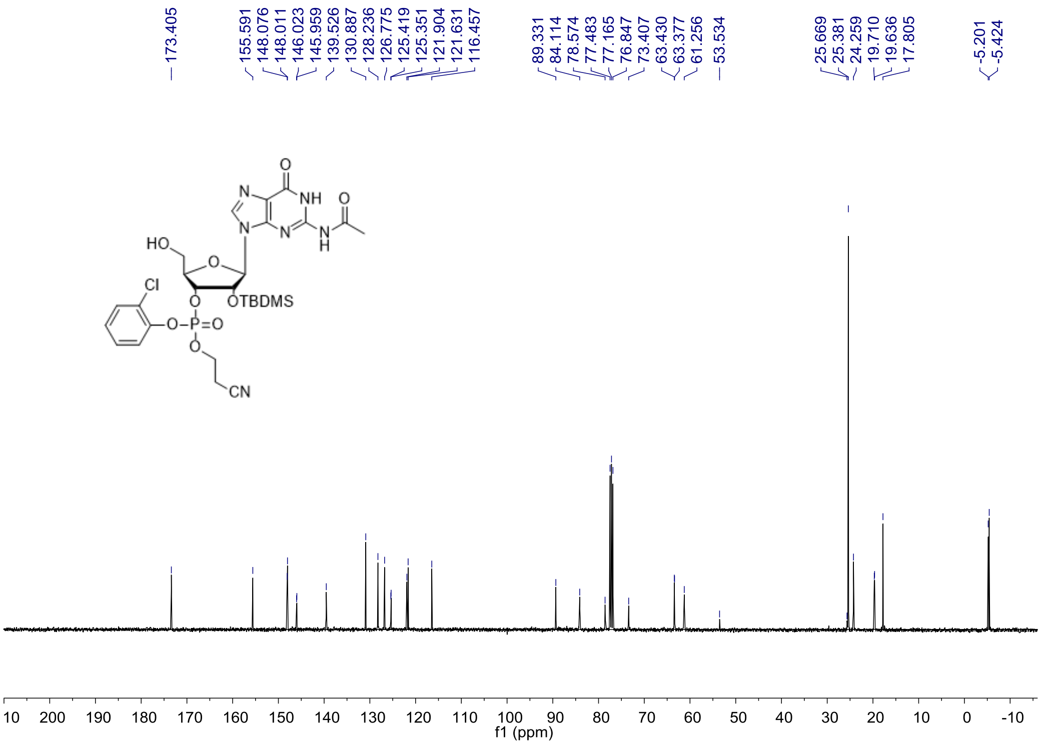

Figure S32. ^{13}C NMR in $CDCl_3$ of **3c**

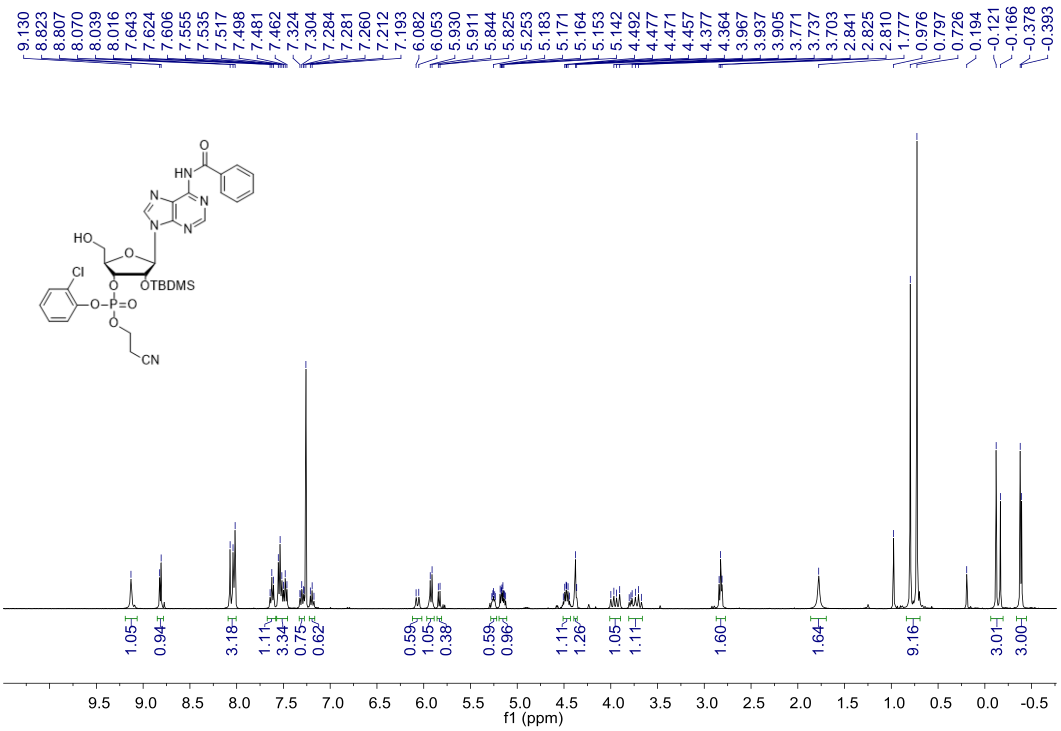

Figure S33. ^{1}H NMR in $CDCl_3$ of **3d**

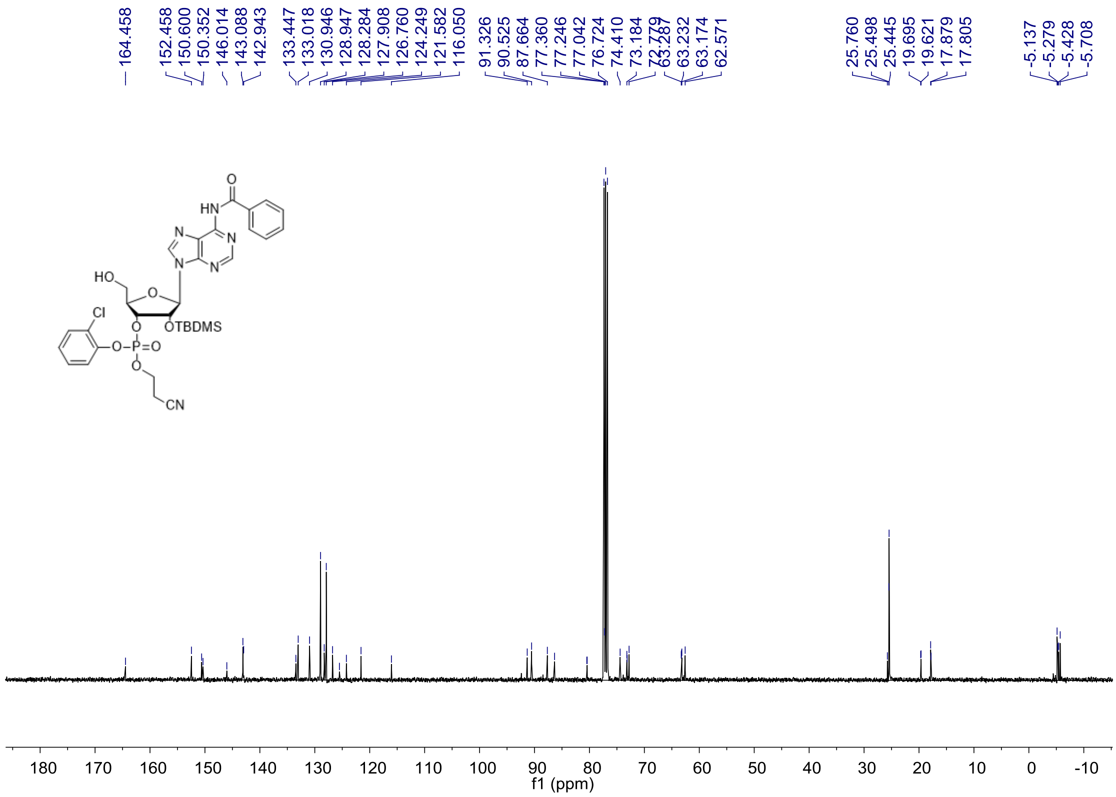

Figure S34. ^{13}C NMR spectrum in $CDCl_3$ of **3d**

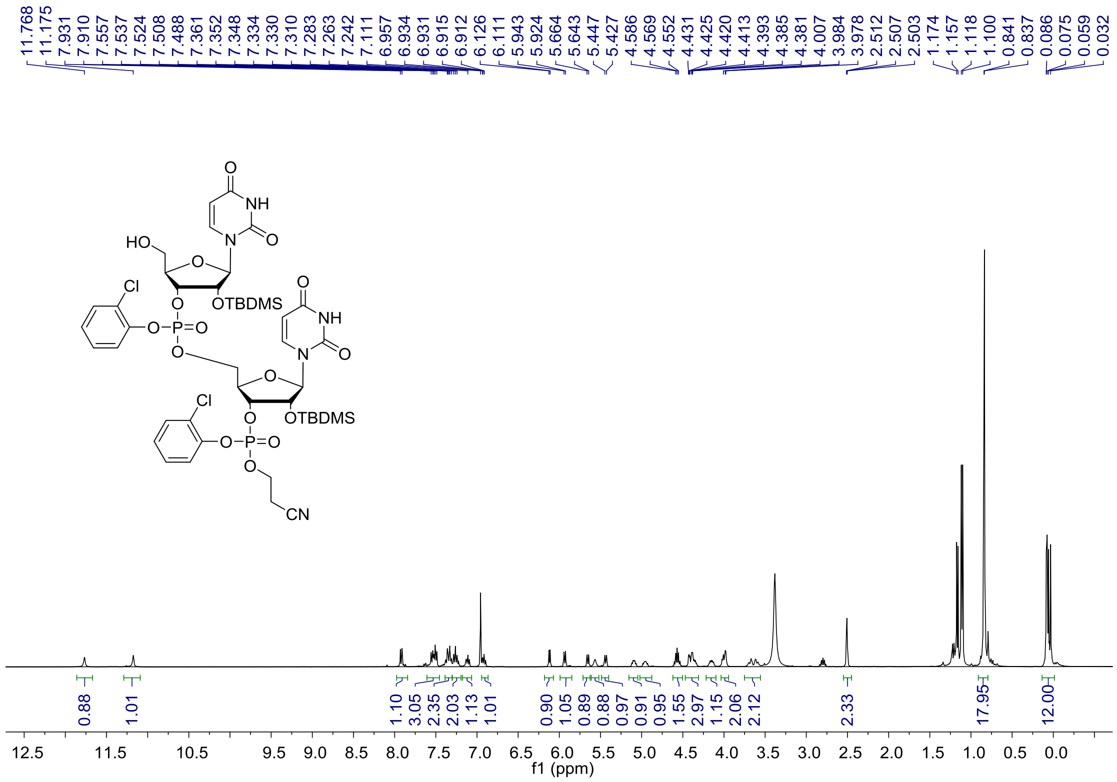

Figure S35. ^{1}H NMR in $CDCl_3$ of **4a**

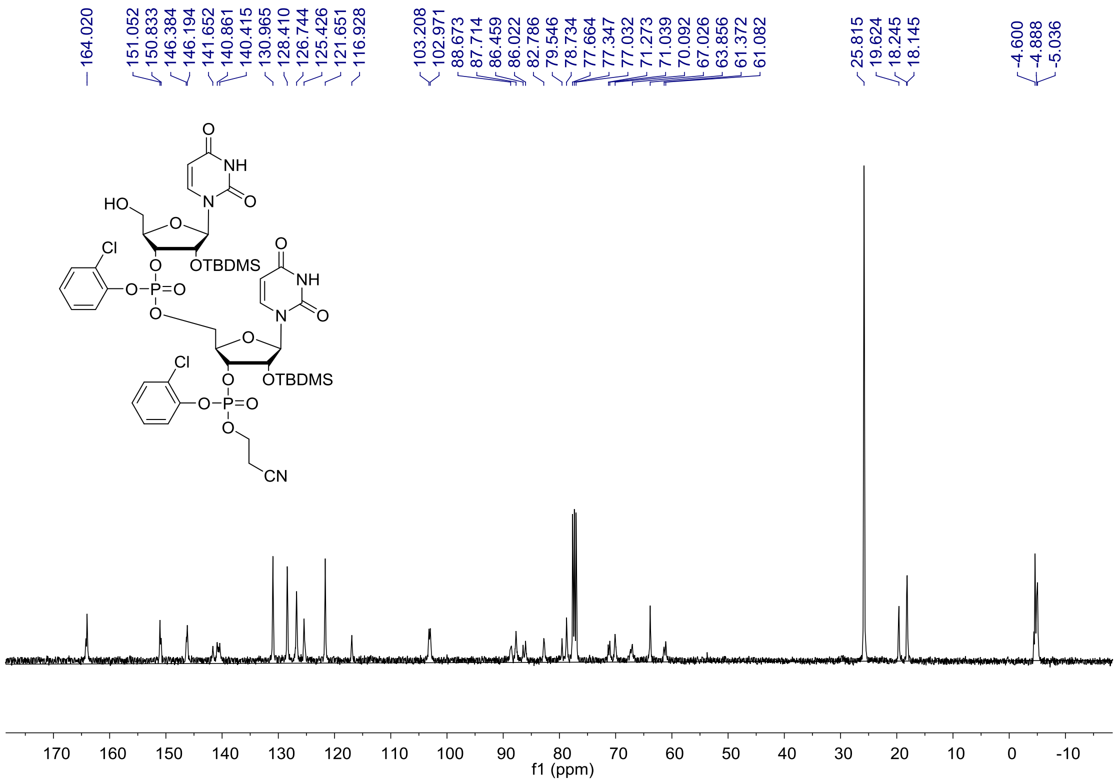

Figure S36. ^{13}C NMR in $CDCl_3$ of **4a**

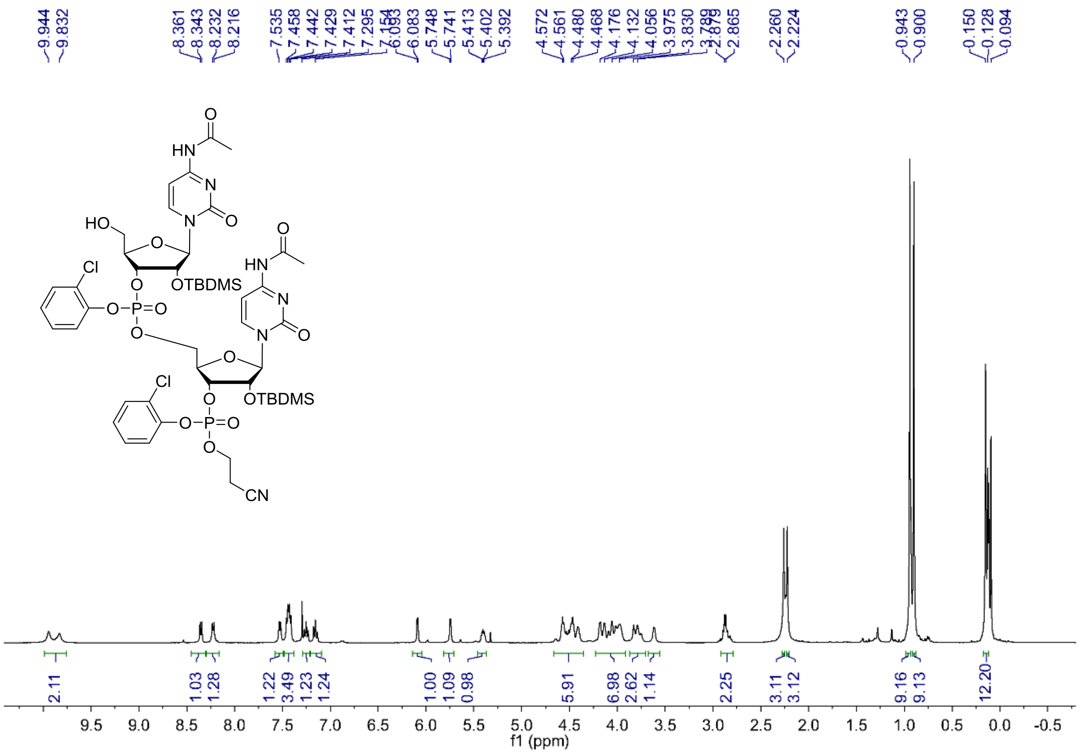

Figure S37. ^{1}H NMR in $CDCl_3$ of **4a**

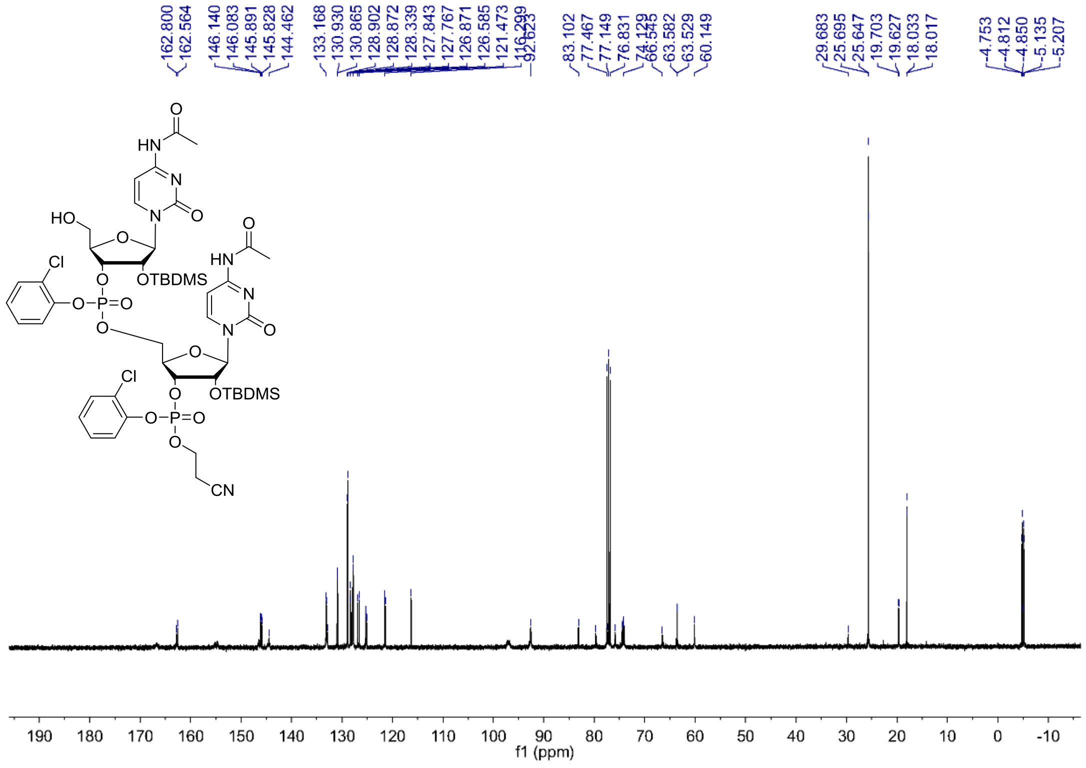

Figure S38. ^{13}C NMR in $CDCl_3$ of **4b**

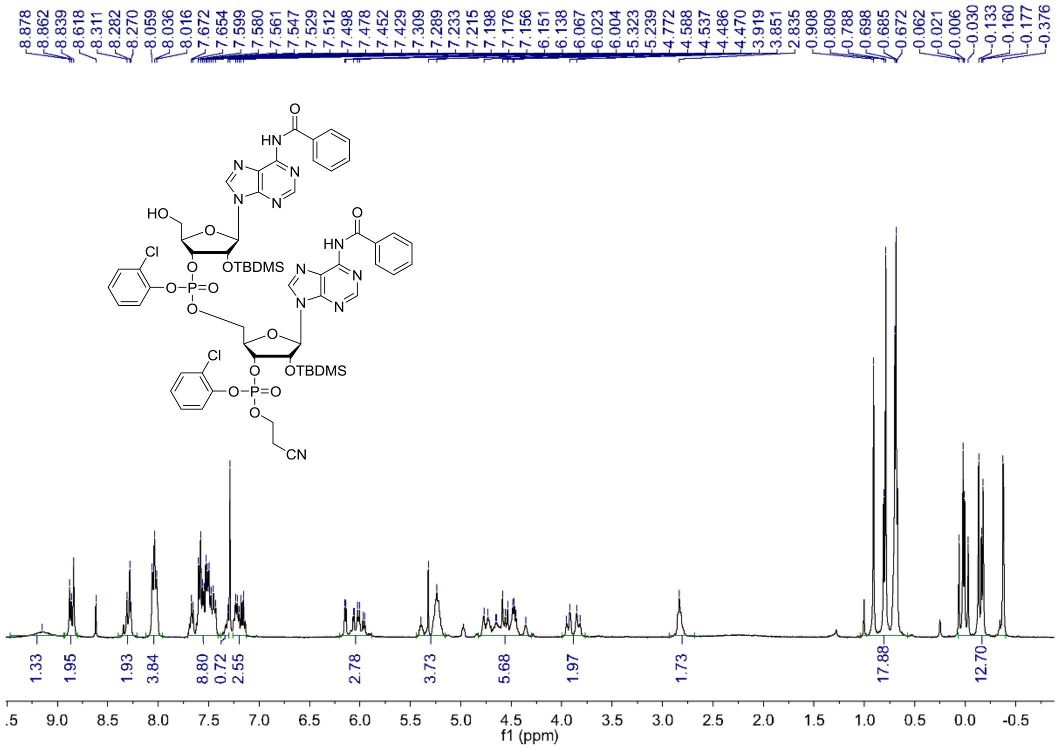

Figure S39. ^{1}H NMR in $CDCl_3$ of **4d**

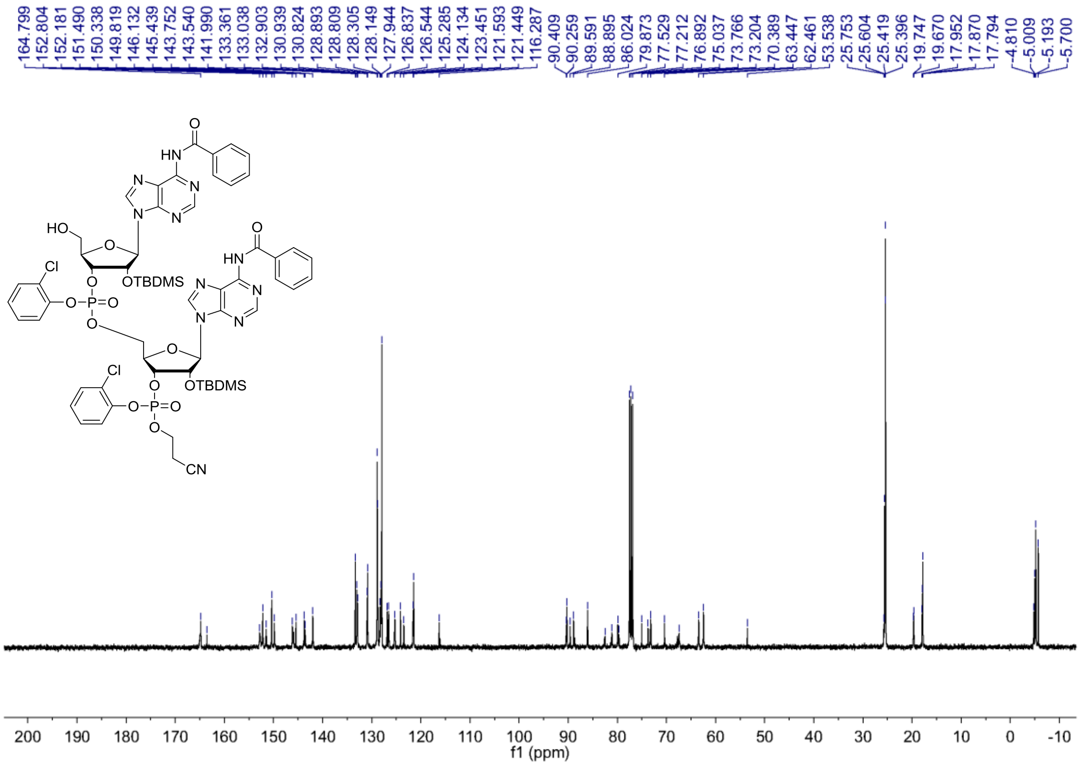

Figure S40. ^{13}C NMR in $CDCl_3$ of **4d**

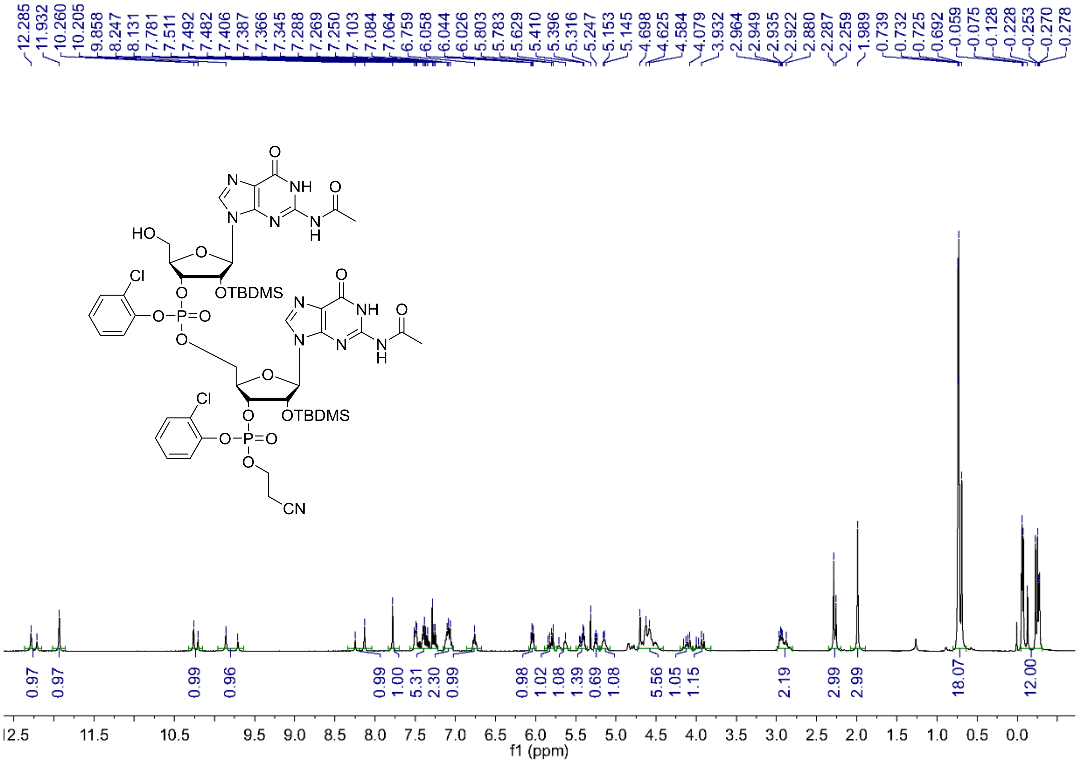

Figure S41. ^{1}H NMR in $CDCl_3$ of **4c**

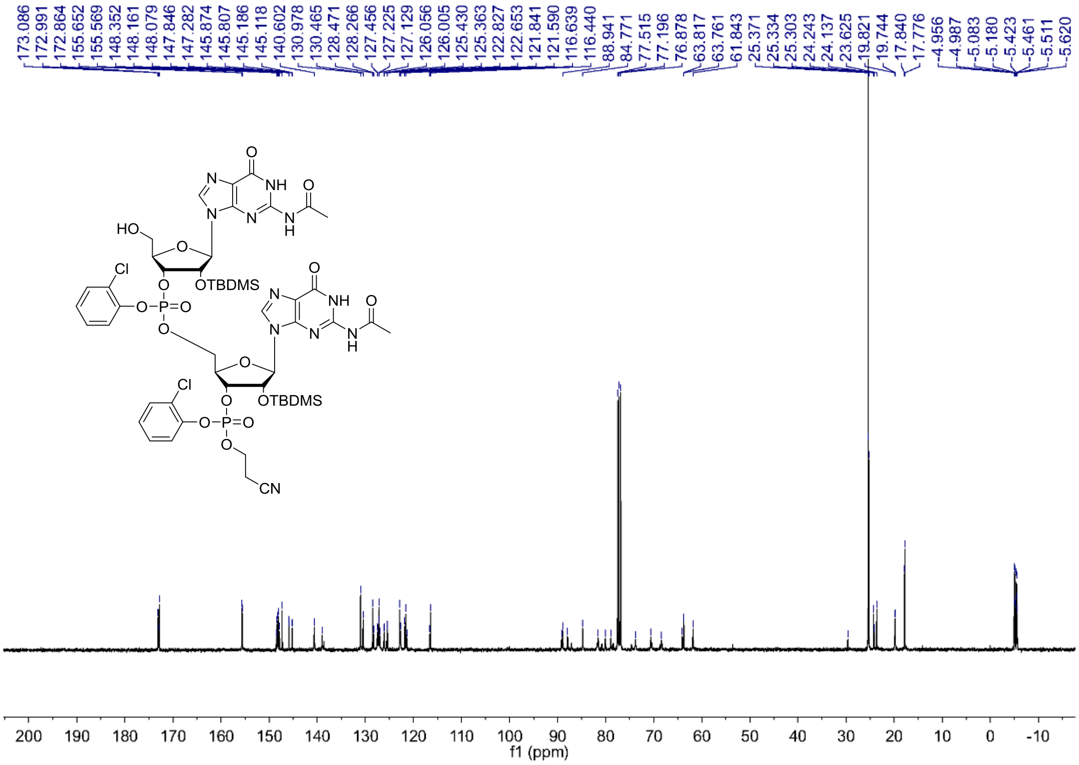

Figure S42. ^{13}C NMR in $CDCl_3$ of **4c**

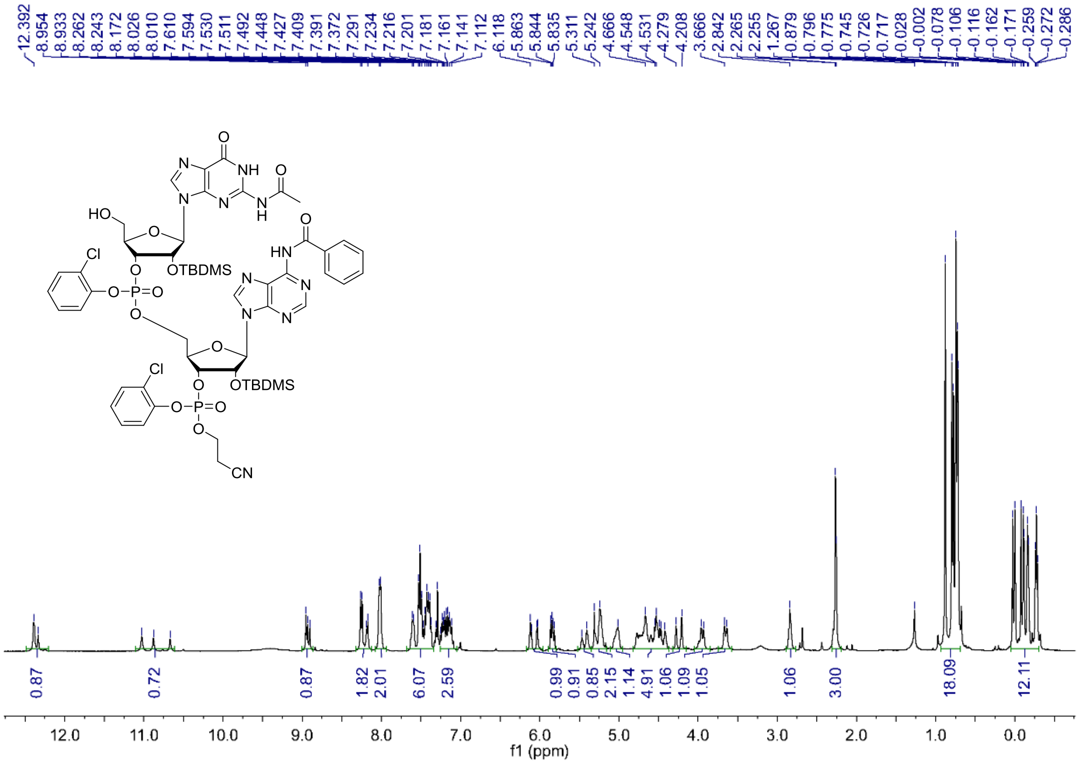

Figure S43. ^{1}H NMR in $CDCl_3$ of **4e**

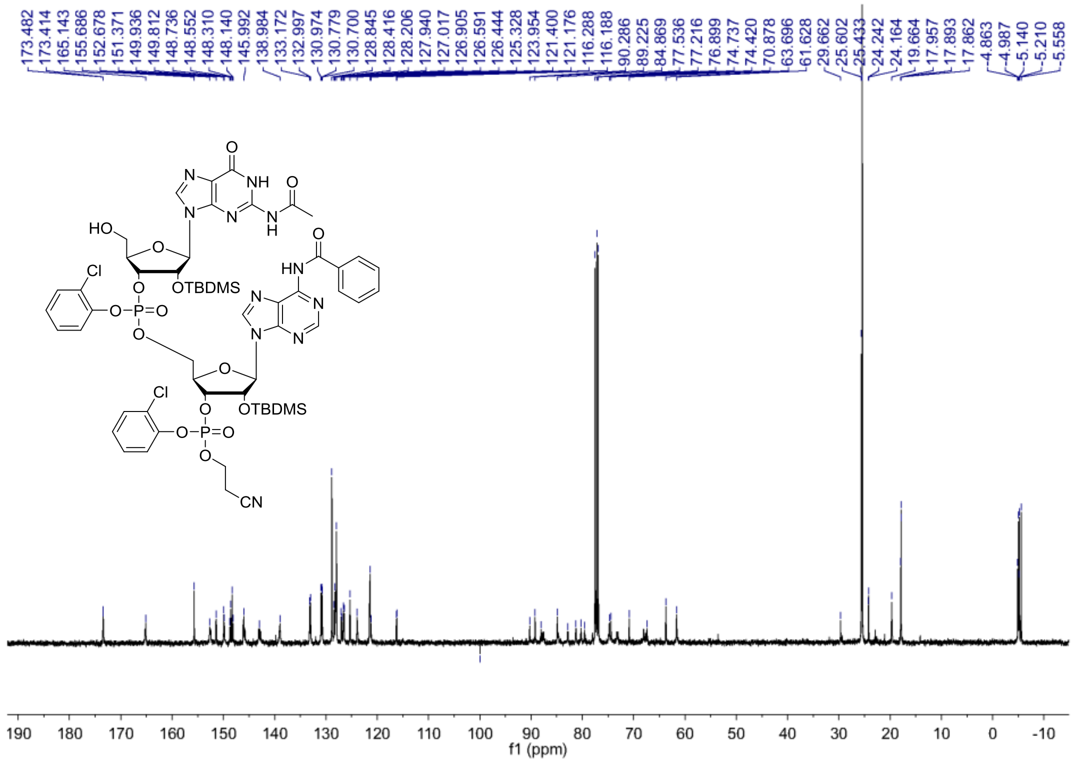

Figure S44. ^{13}C NMR in $CDCl_3$ of **4e**

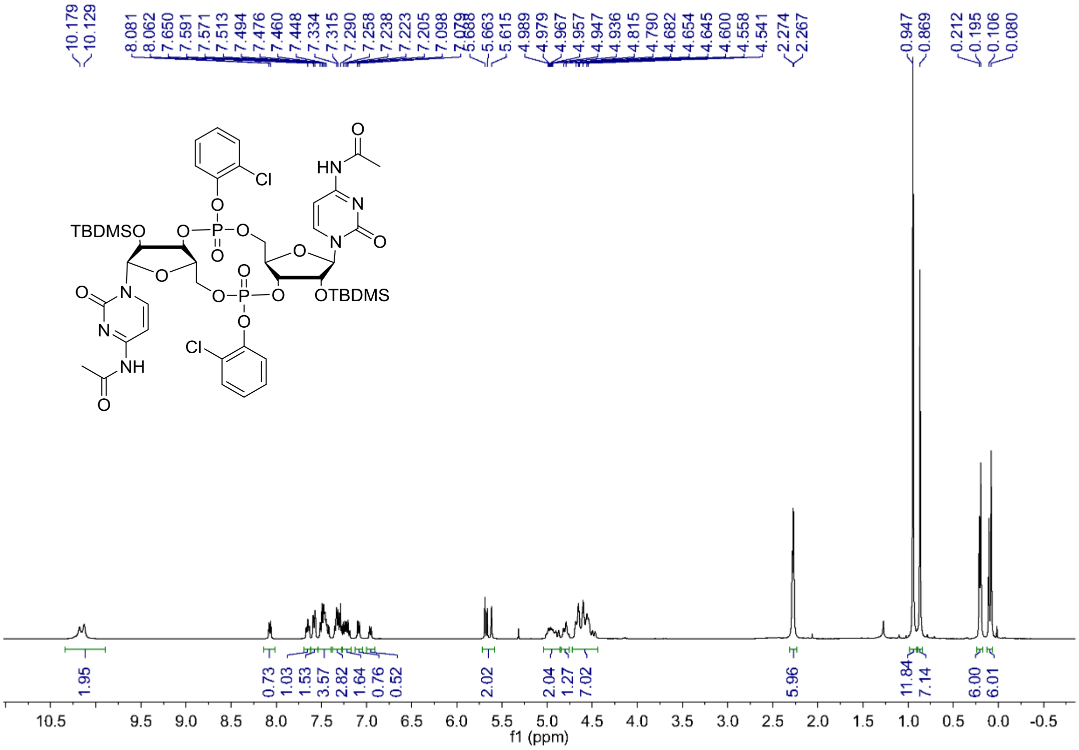

Figure S45. ^{1}H NMR in $CDCl_3$ of **5b**

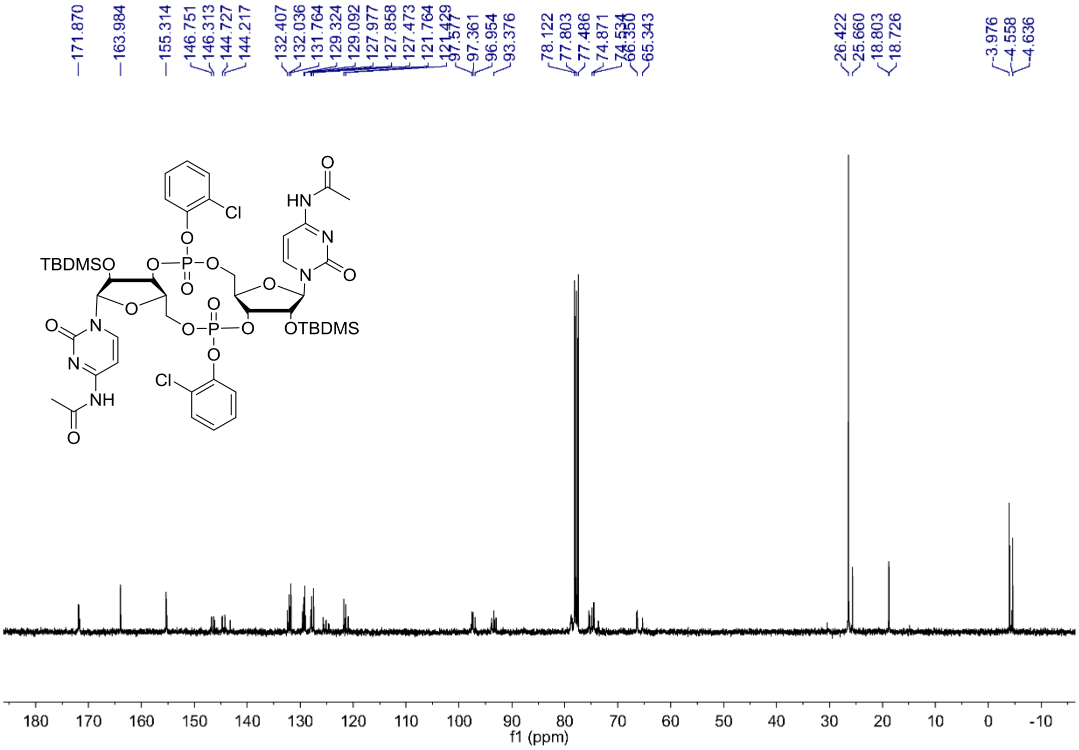

Figure S46. ^{13}C NMR in $CDCl_3$ of **5b**

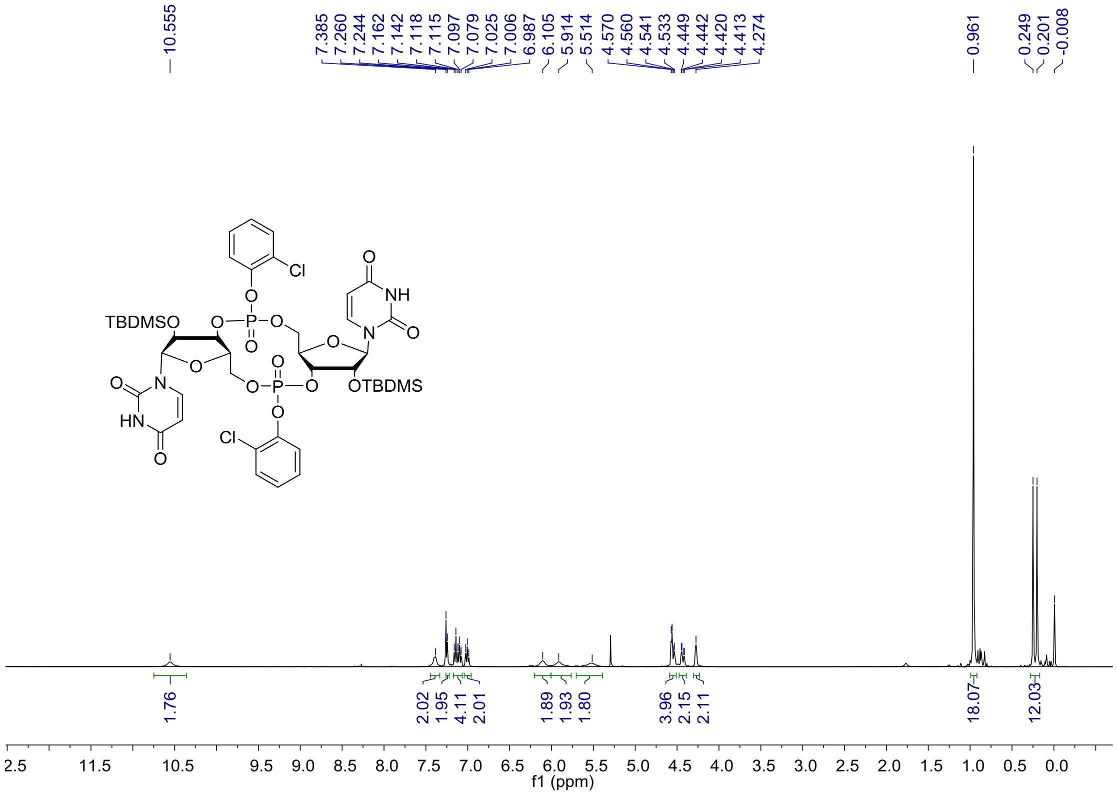

Figure S47. ^{1}H NMR in $CDCl_3$ of **5a**

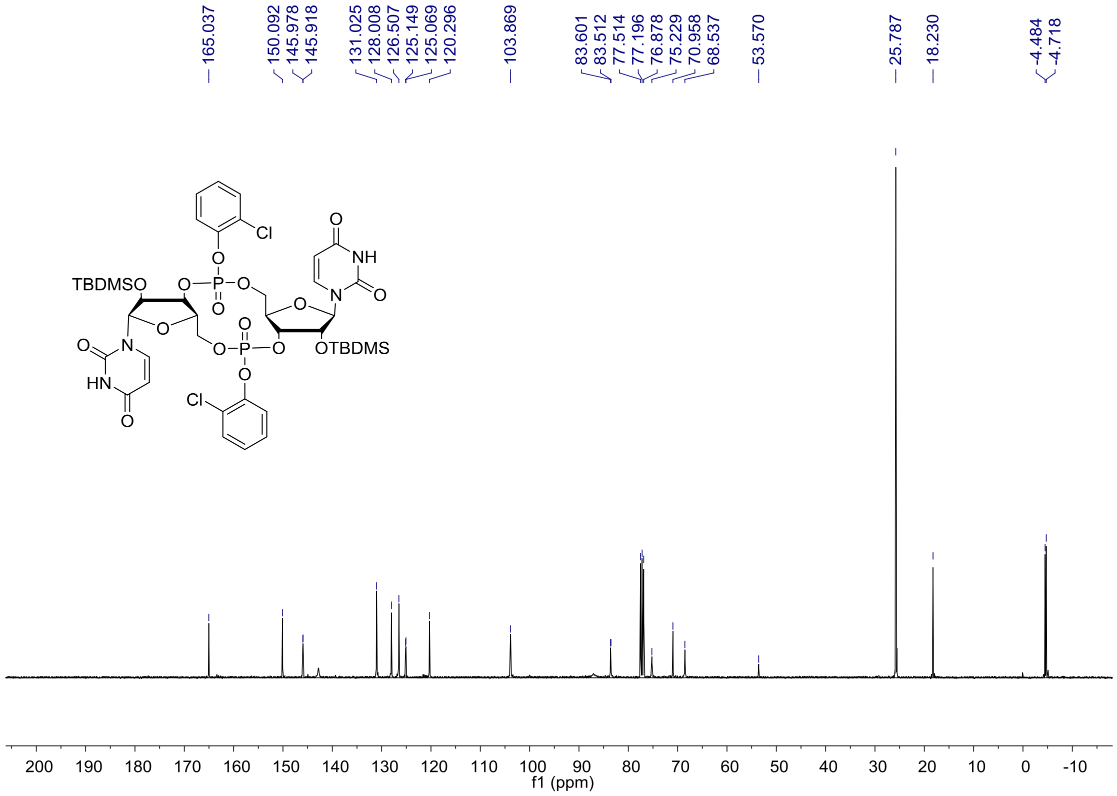

Figure S48. ^{13}C NMR in $CDCl_3$ of **5a**

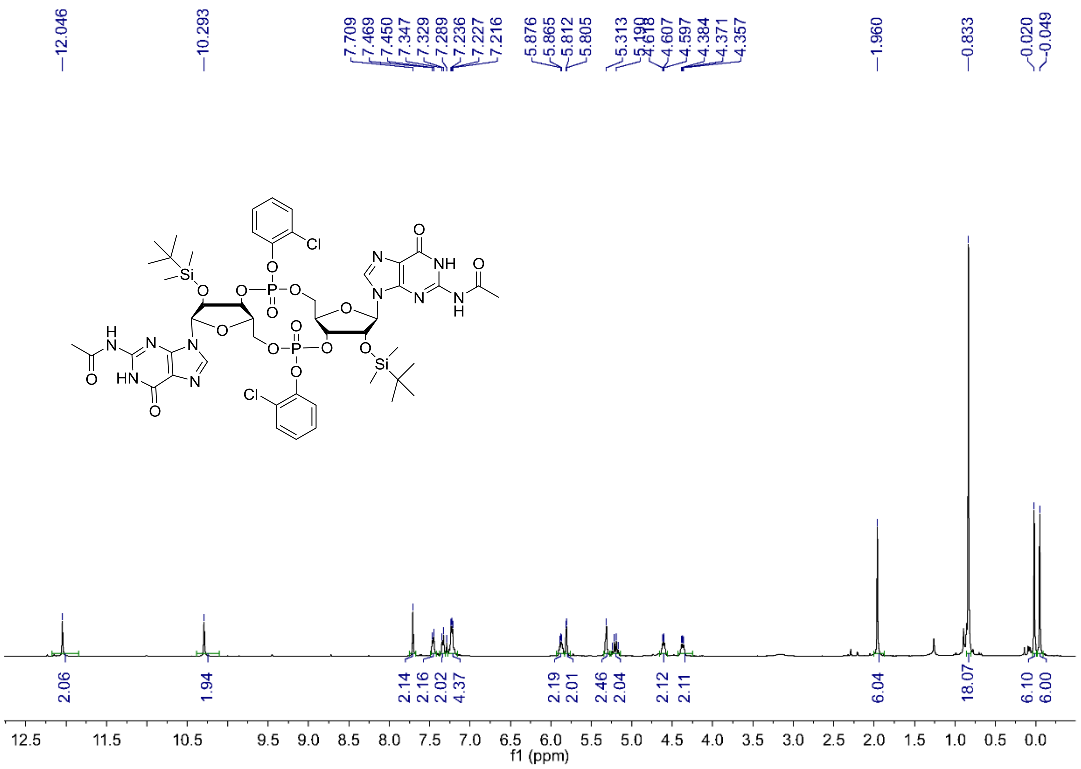

Figure S49. ^{1}H NMR in $CDCl_3$ of **5c**

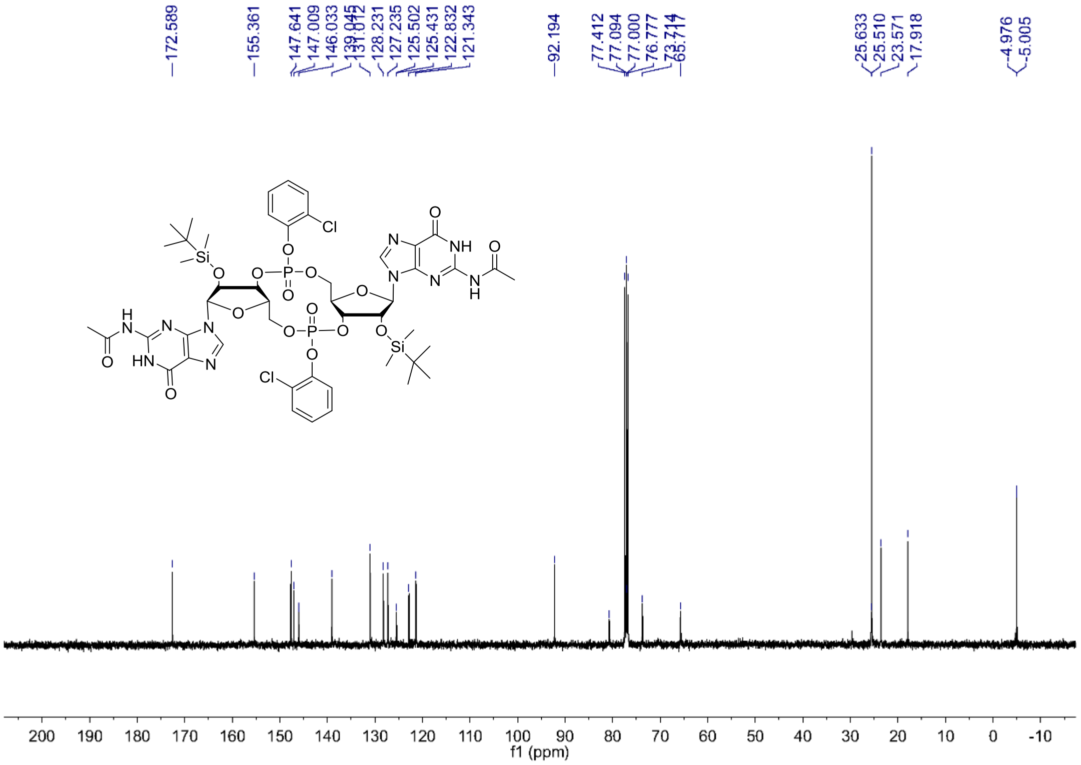

Figure S50. ^{13}C NMR in $CDCl_3$ of **5c**

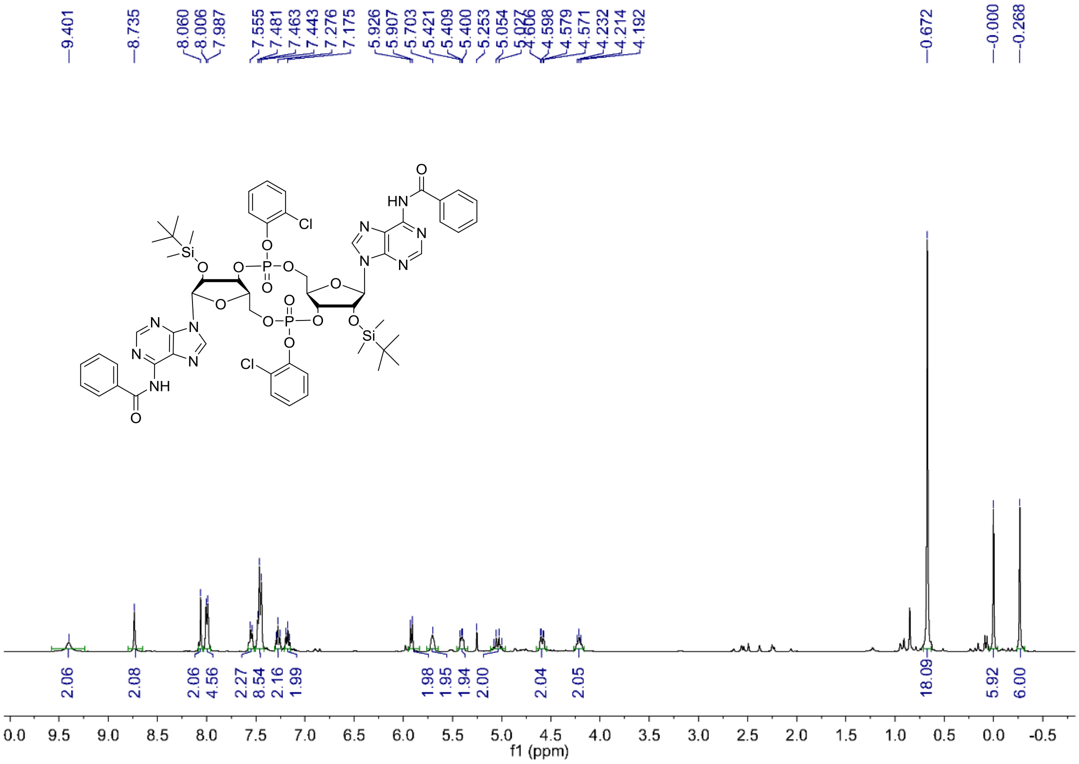

Figure S51. ^{1}H NMR in $CDCl_3$ of **5d**

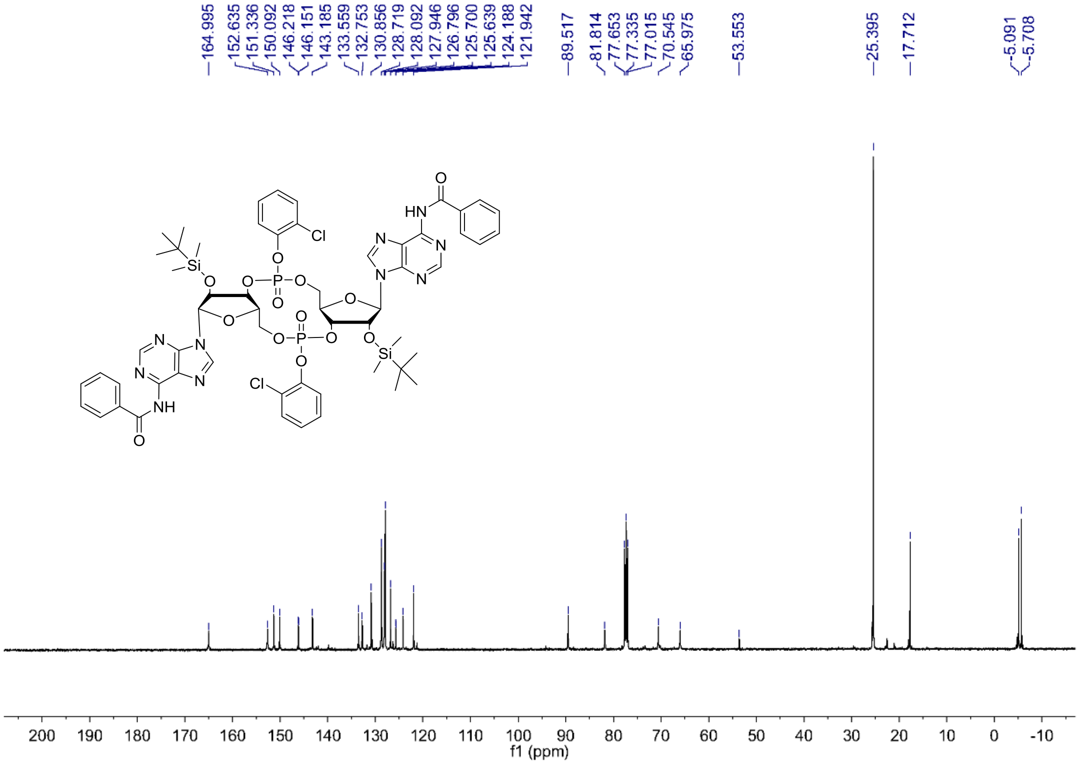

Figure S52. ^{13}C NMR in $CDCl_3$ of **5d**

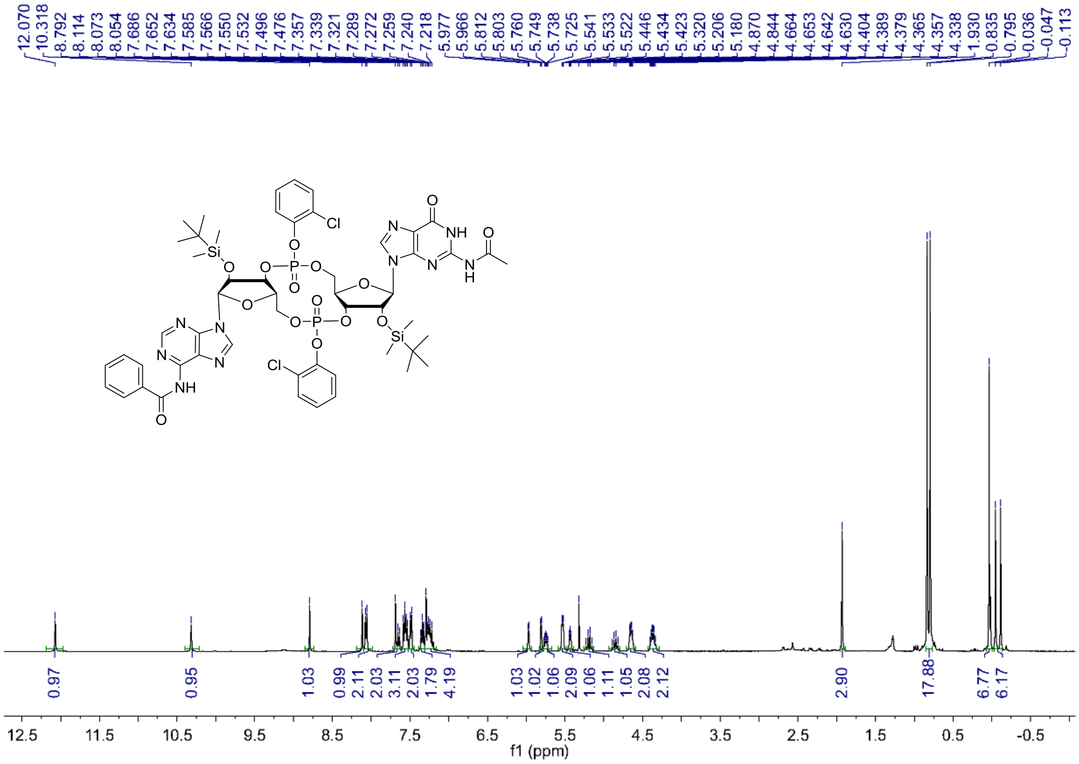

Figure S53. ^{1}H NMR in $CDCl_3$ of **5e**

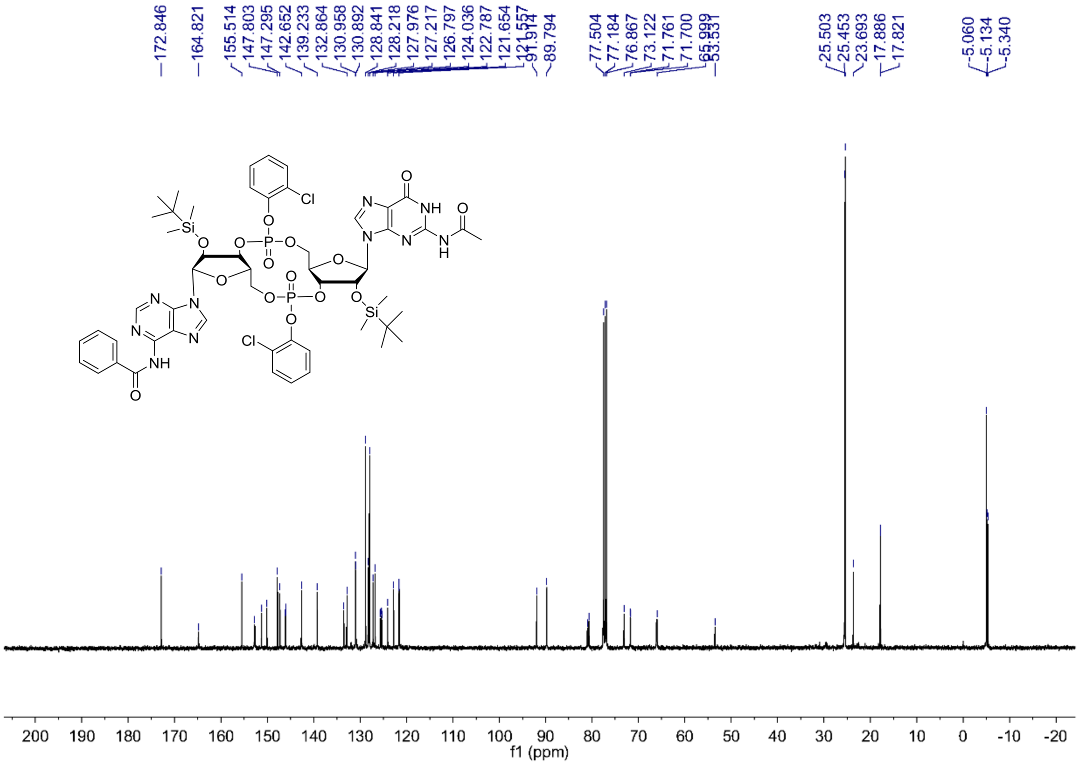

Figure S54. ^{13}C NMR in $CDCl_3$ of **5e**

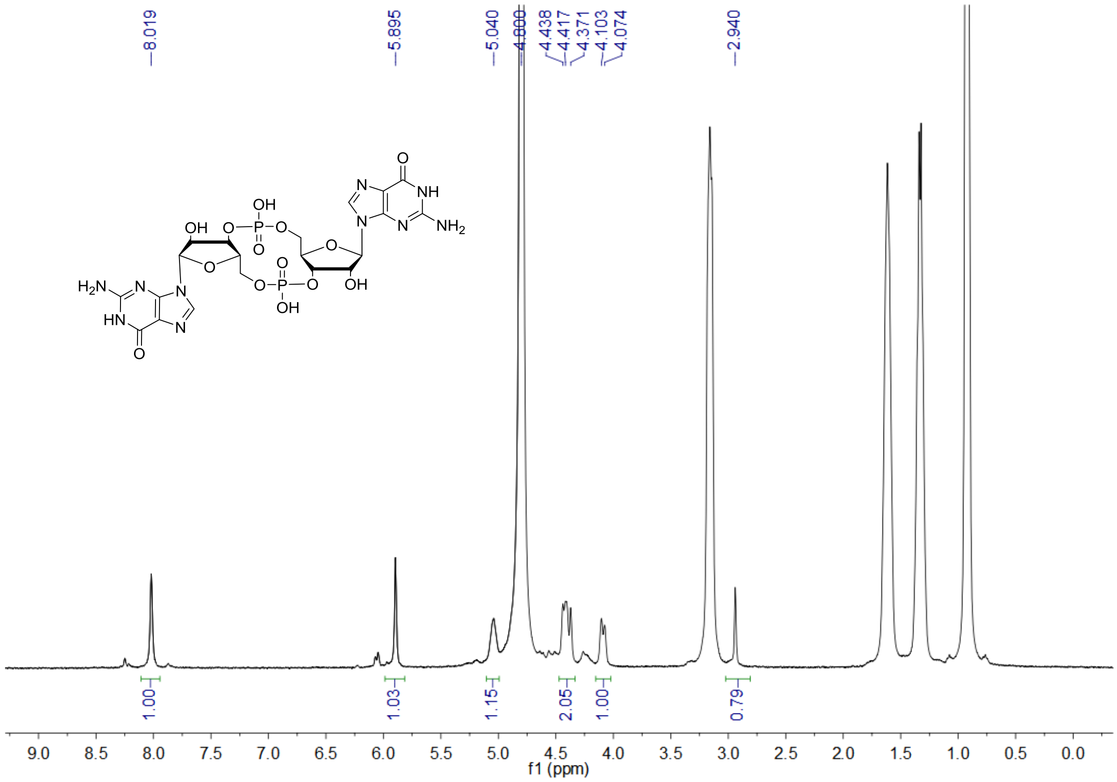

Figure S55. ^{1}H NMR in D_2O of **6c**

High resolution mass spectrometry (HRMS) for the characterization of the compound during the synthesis

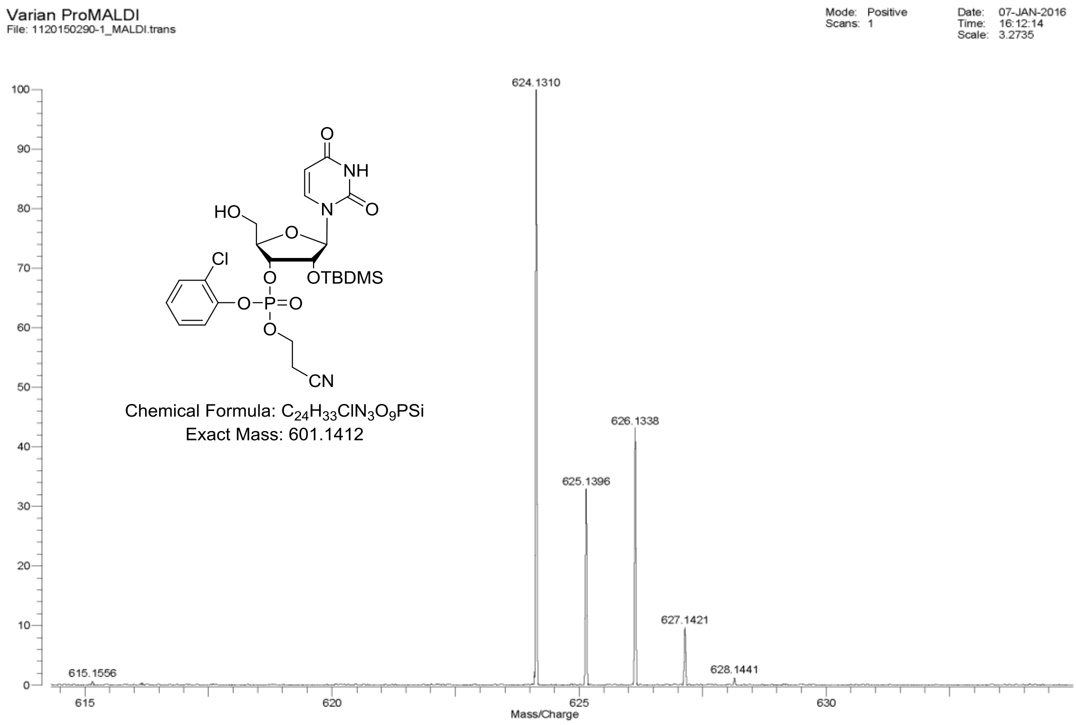

Figure S56. HRMS spectrum of **3a**

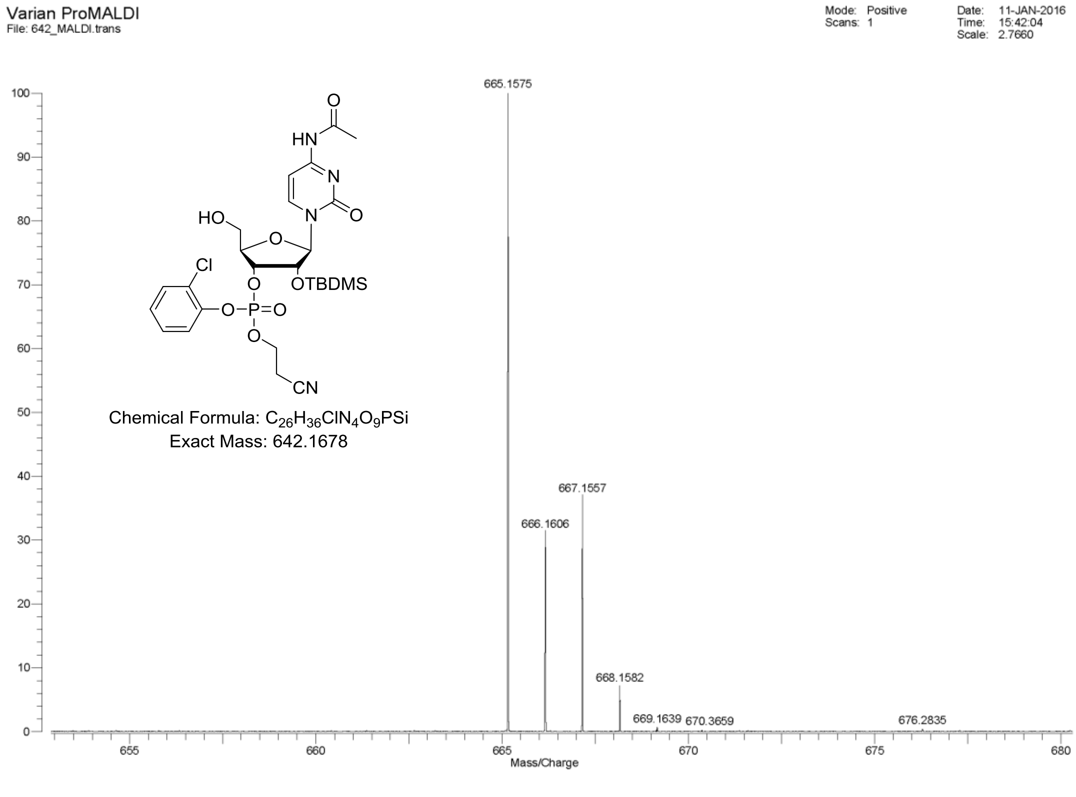

Figure S57. HRMS spectrum of **3b**

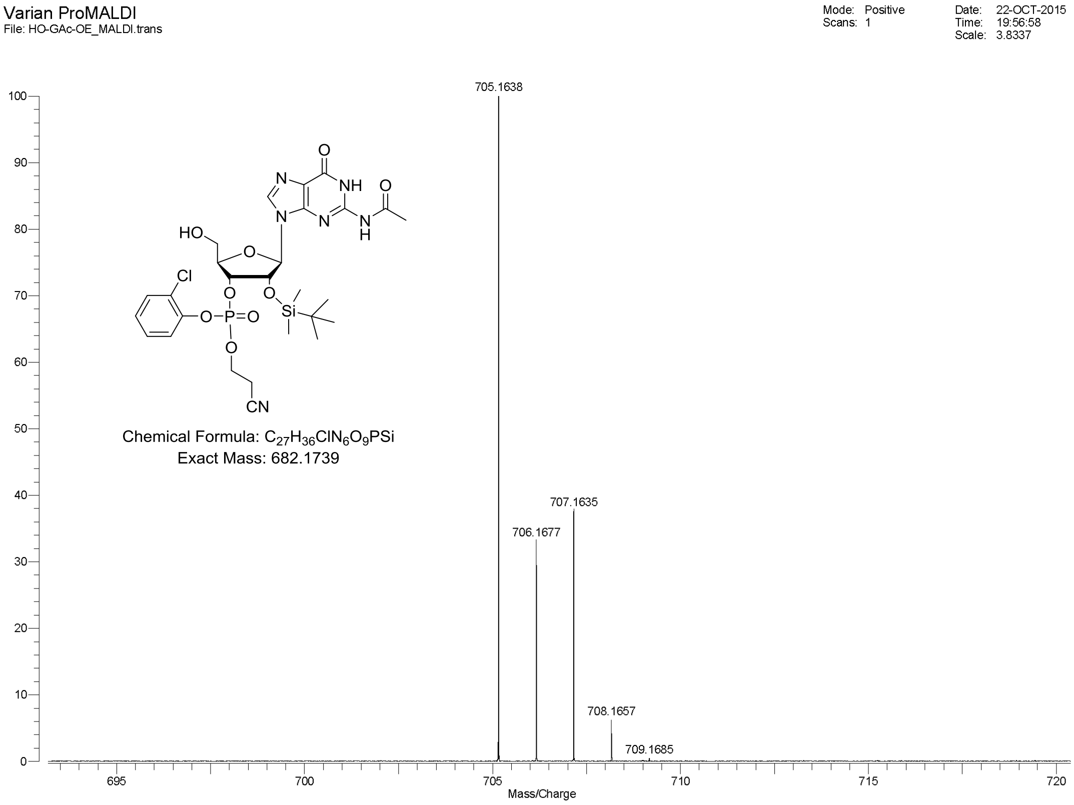

Figure S58. HRMS spectrum of **3c**

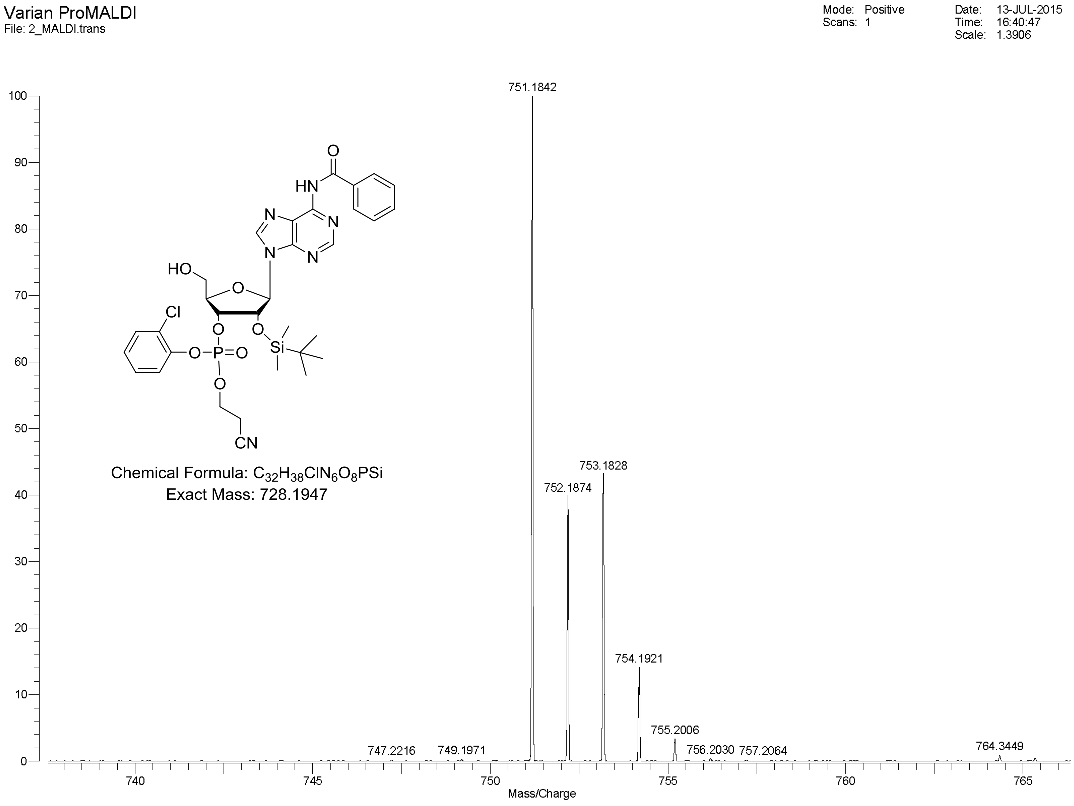

Figure S59. HRMS spectrum of **3d**

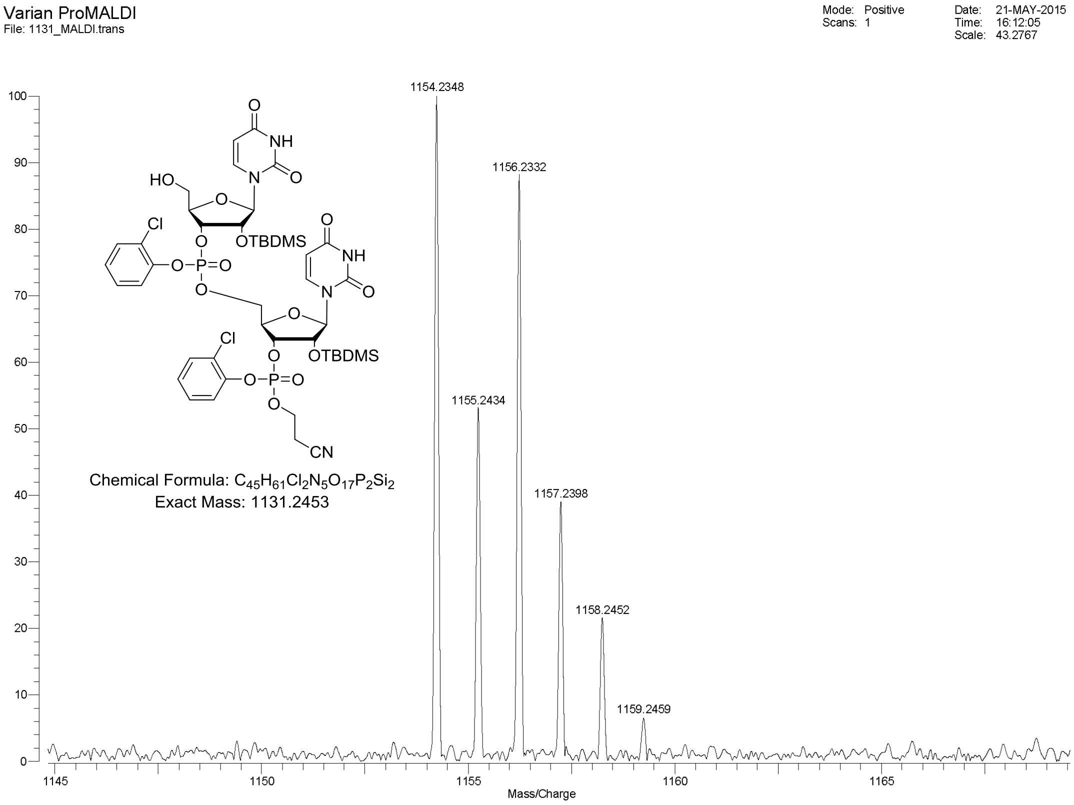

Figure S60. HRMS spectrum of **4a**

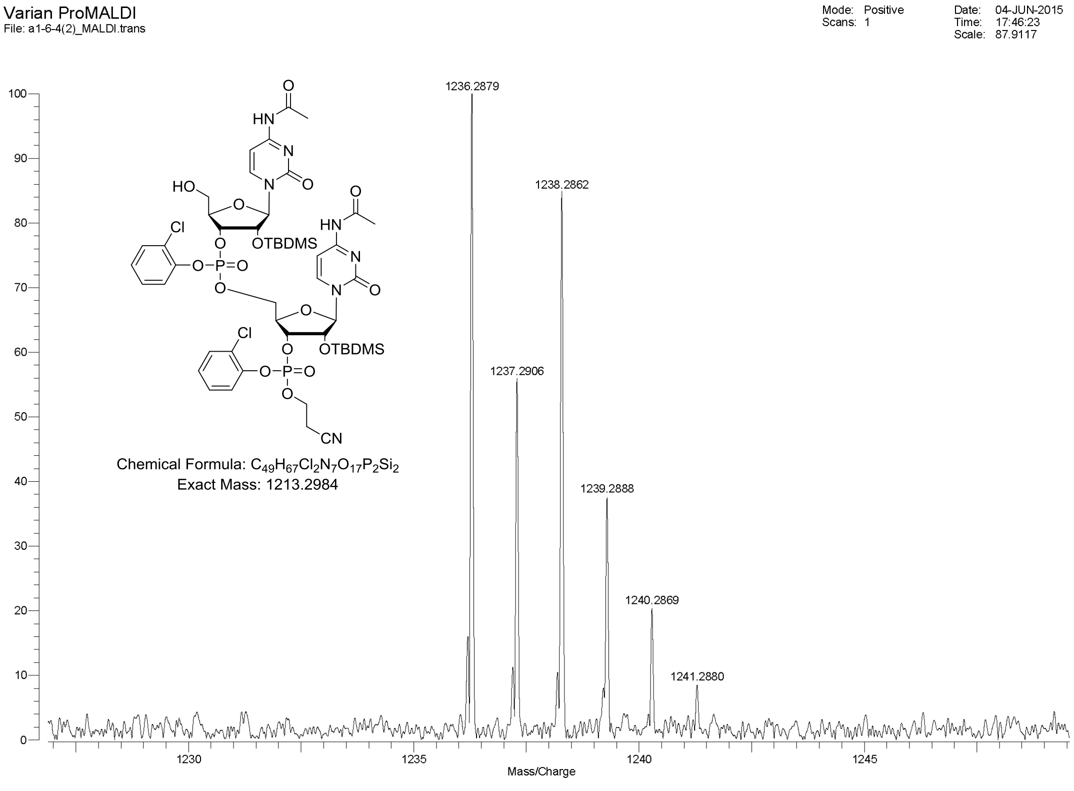

Figure S61. HRMS spectrum of **4b**

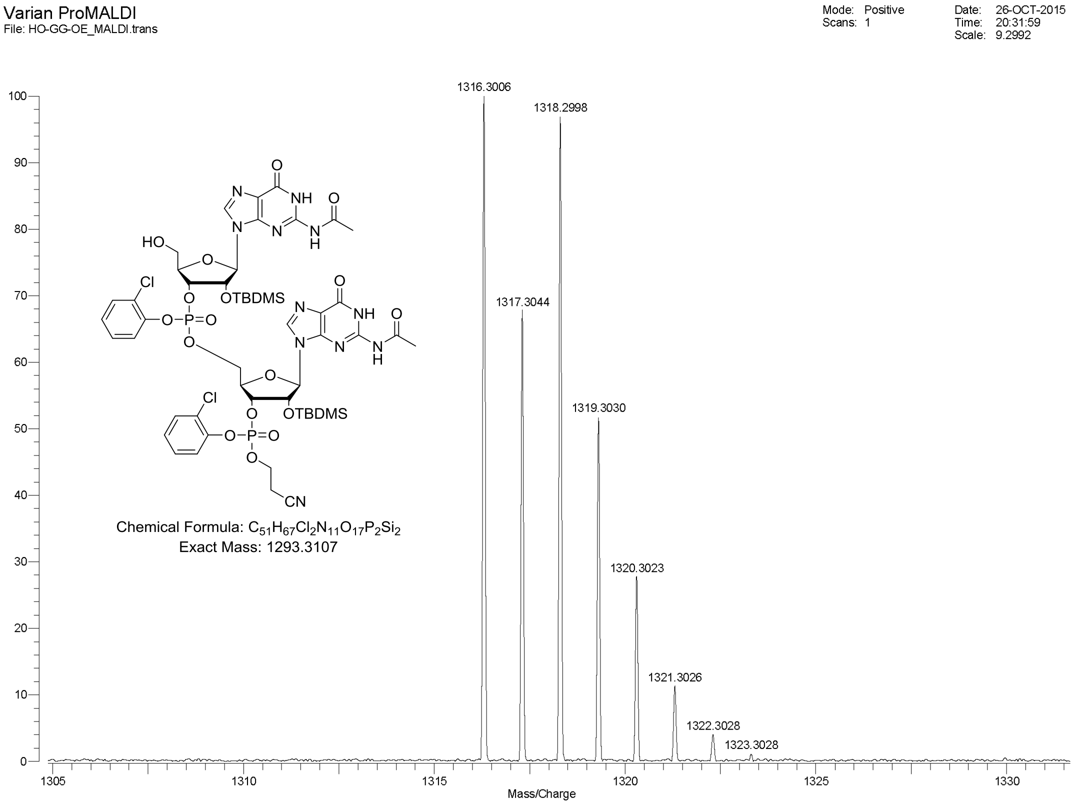

Figure S62. HRMS spectrum of **4c**

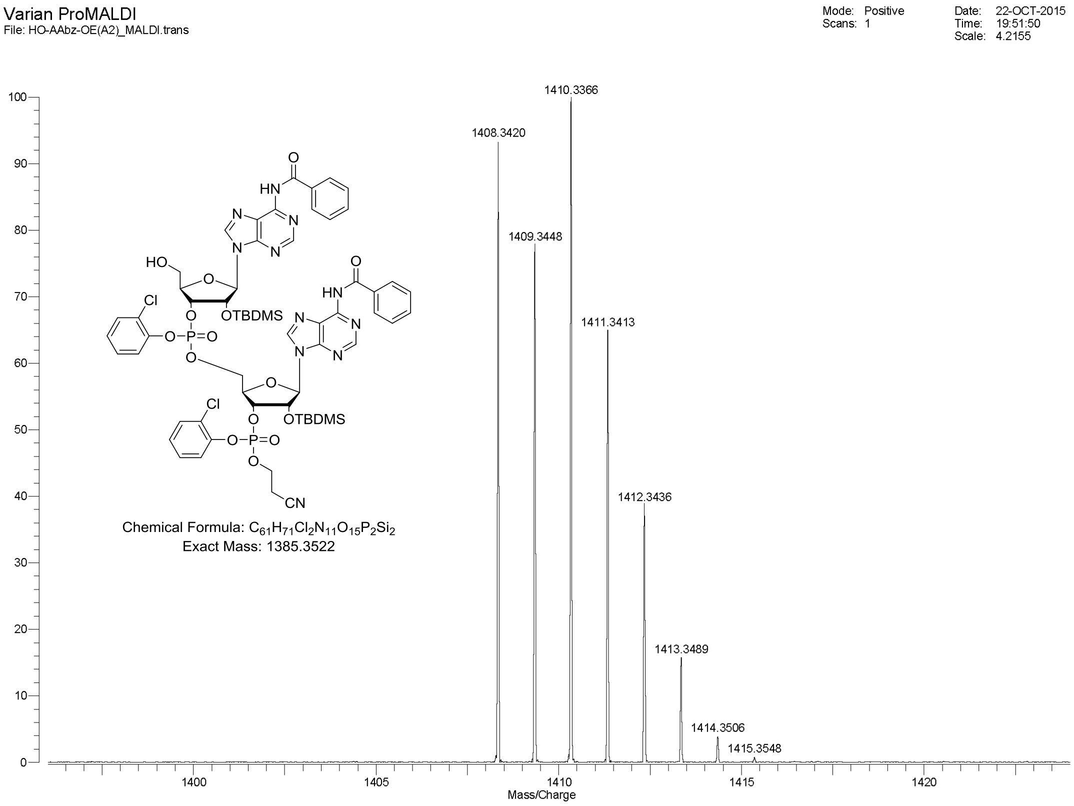

Figure S63. HRMS spectrum of **4d**

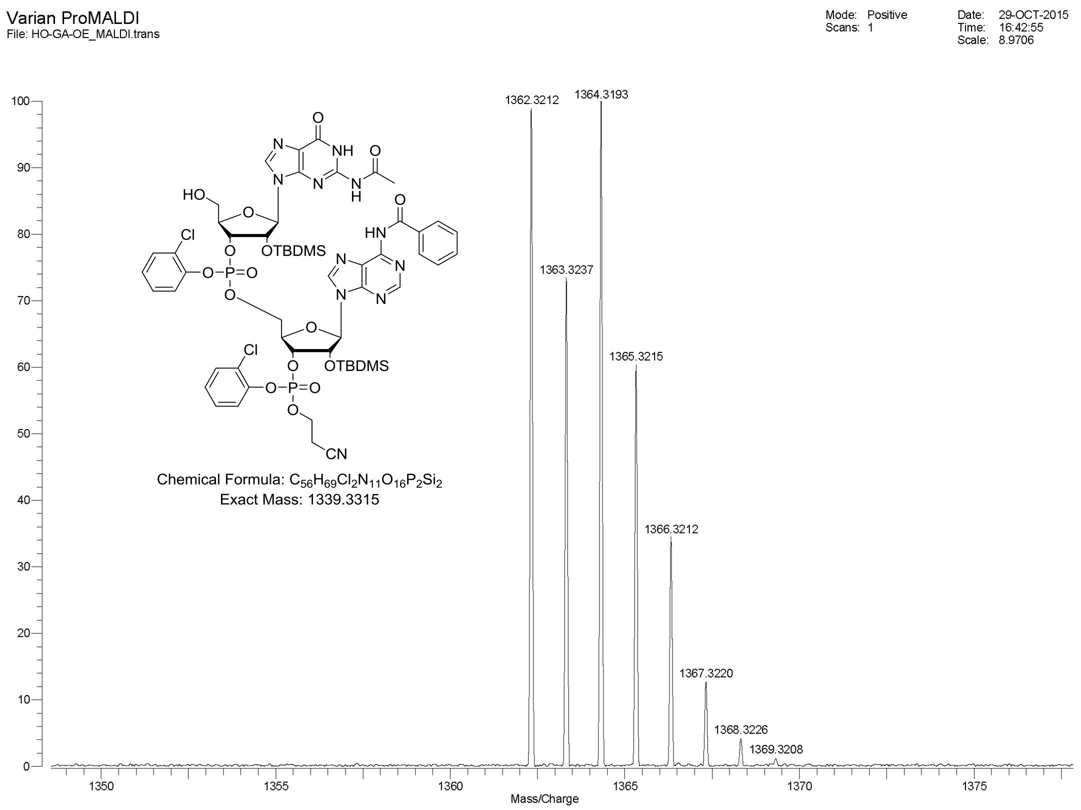

Figure S64. HRMS spectrum of **4e**

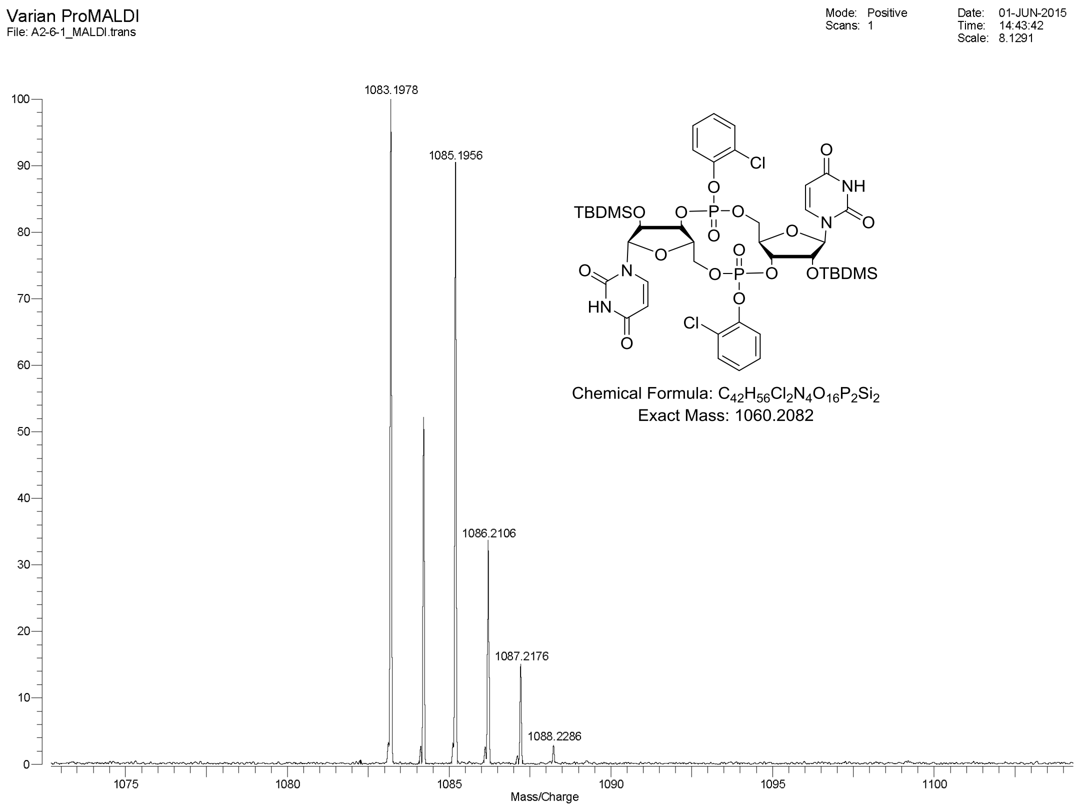

Figure S65. HRMS spectrum of **5a**

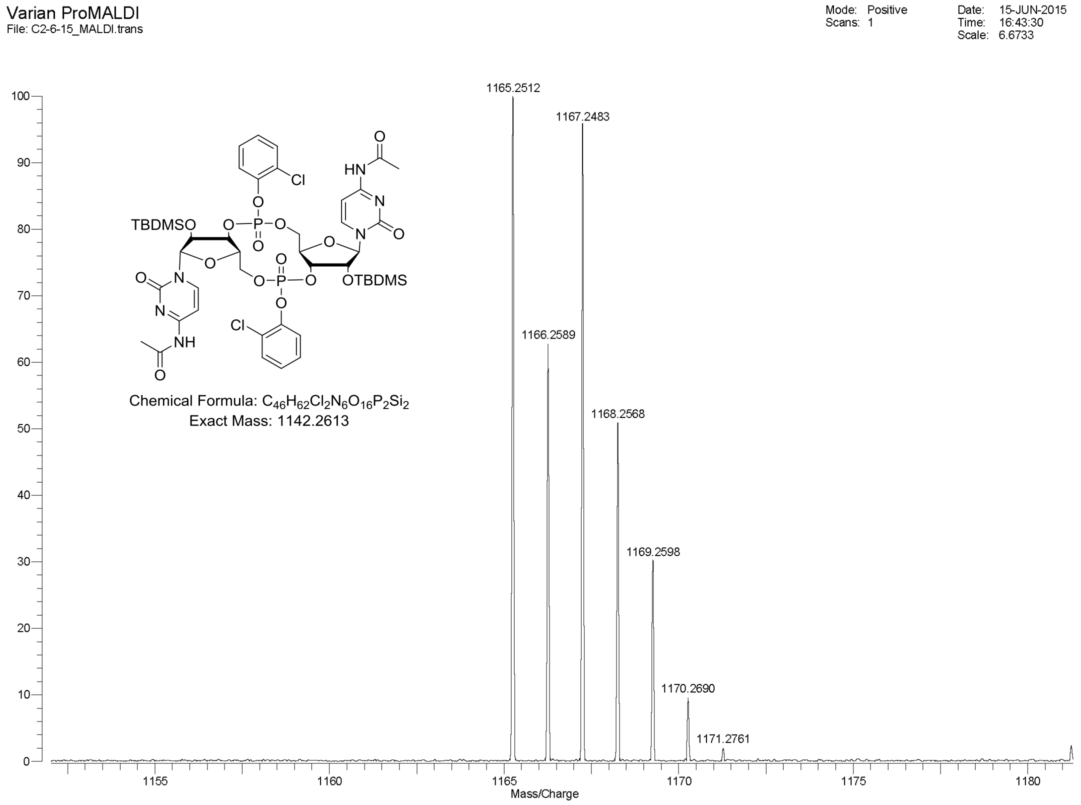

Figure S66. HRMS spectrum of **5b**

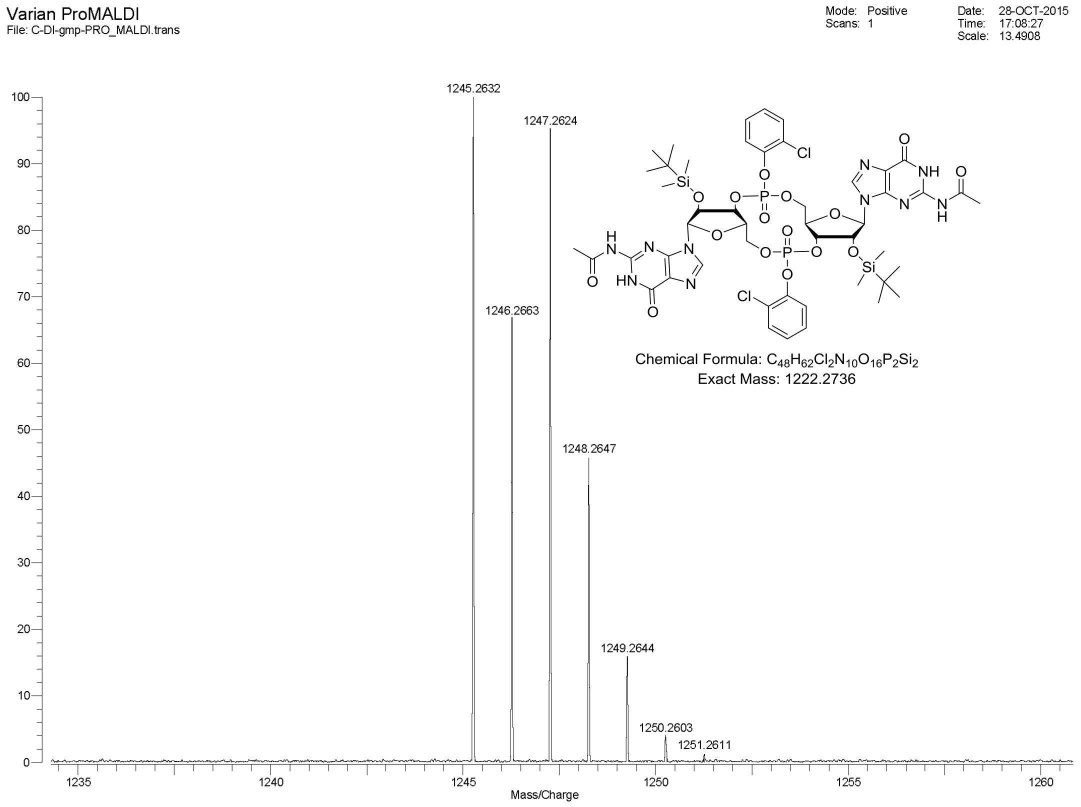

Figure S67. HRMS spectrum of **5c**

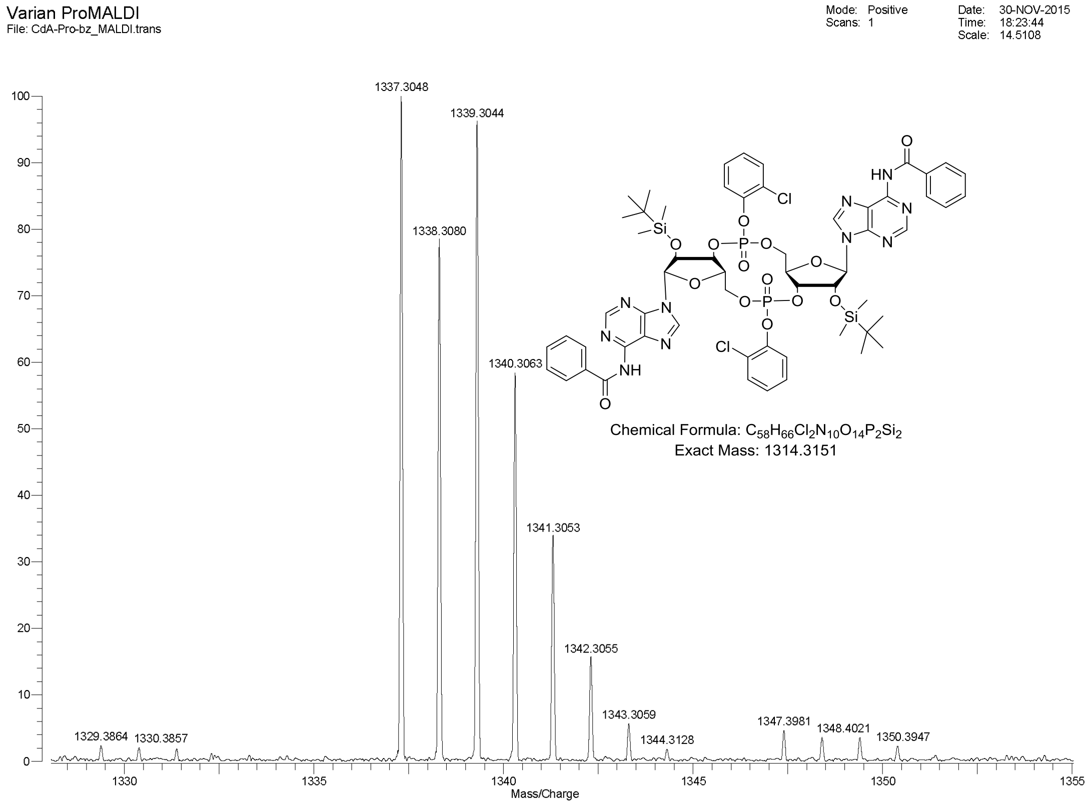

Figure S68. HRMS spectrum of **5d**

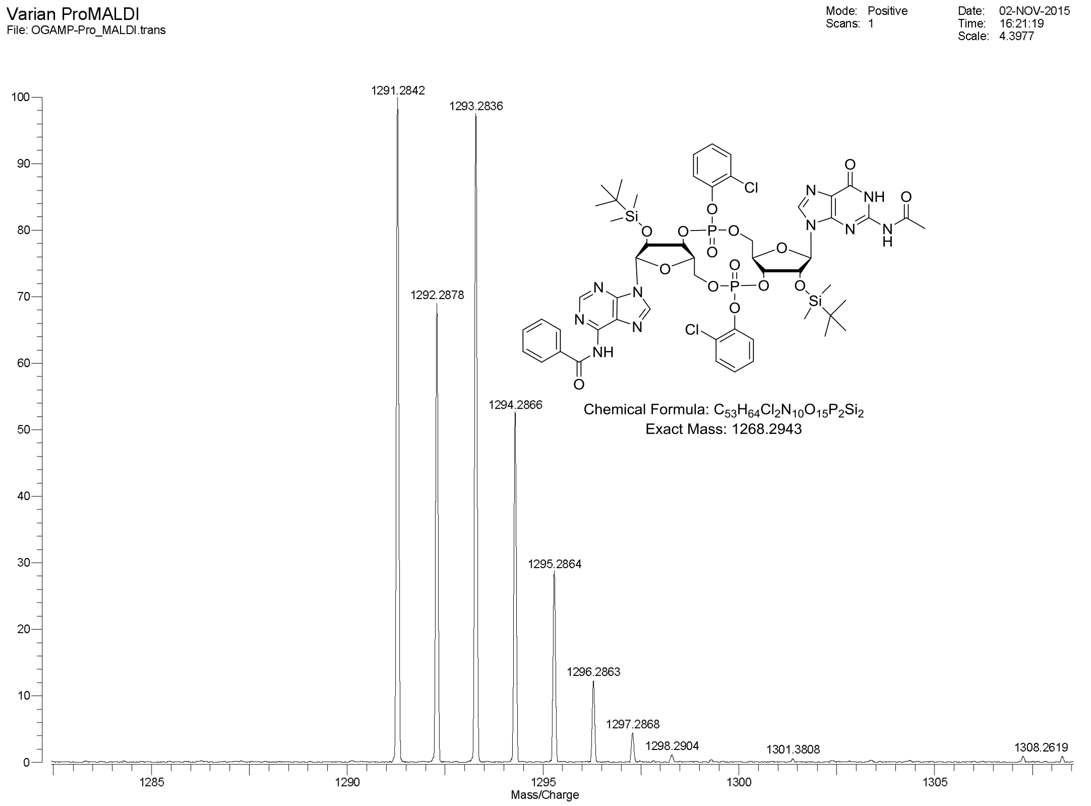

Figure S69. HRMS spectrum of **5e**

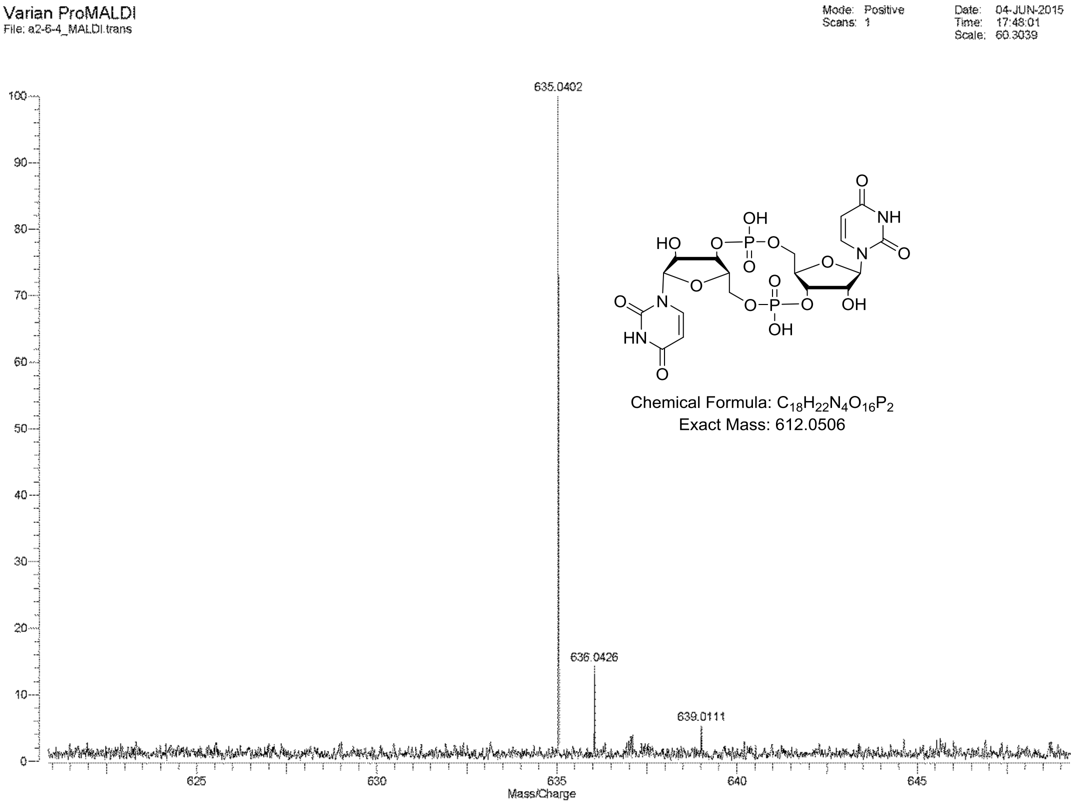

Figure S70. HRMS spectrum of **6a**

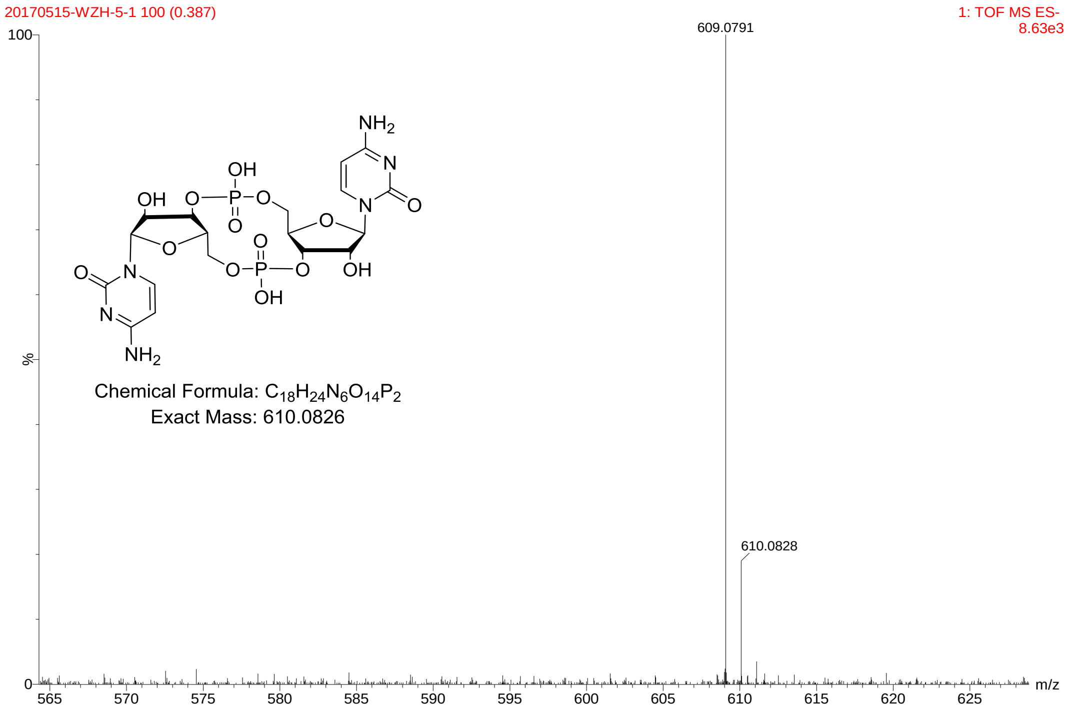

Figure S71. HRMS spectrum of **6b**

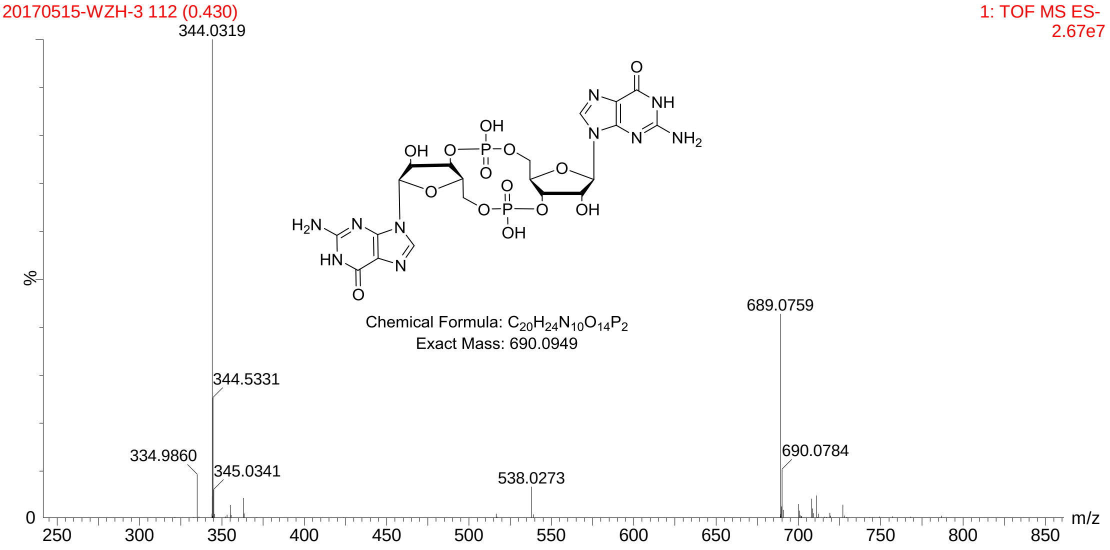

Figure S72. HRMS spectrum of **6c**

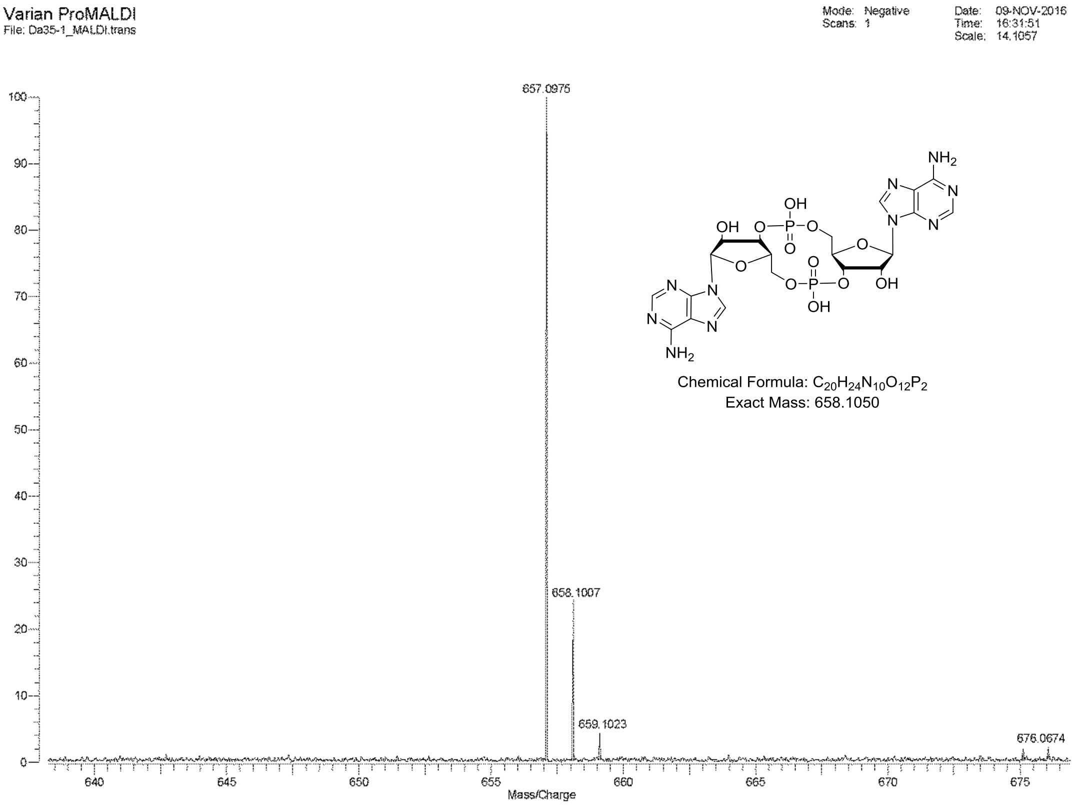

Figure S73. HRMS spectrum of **6d**

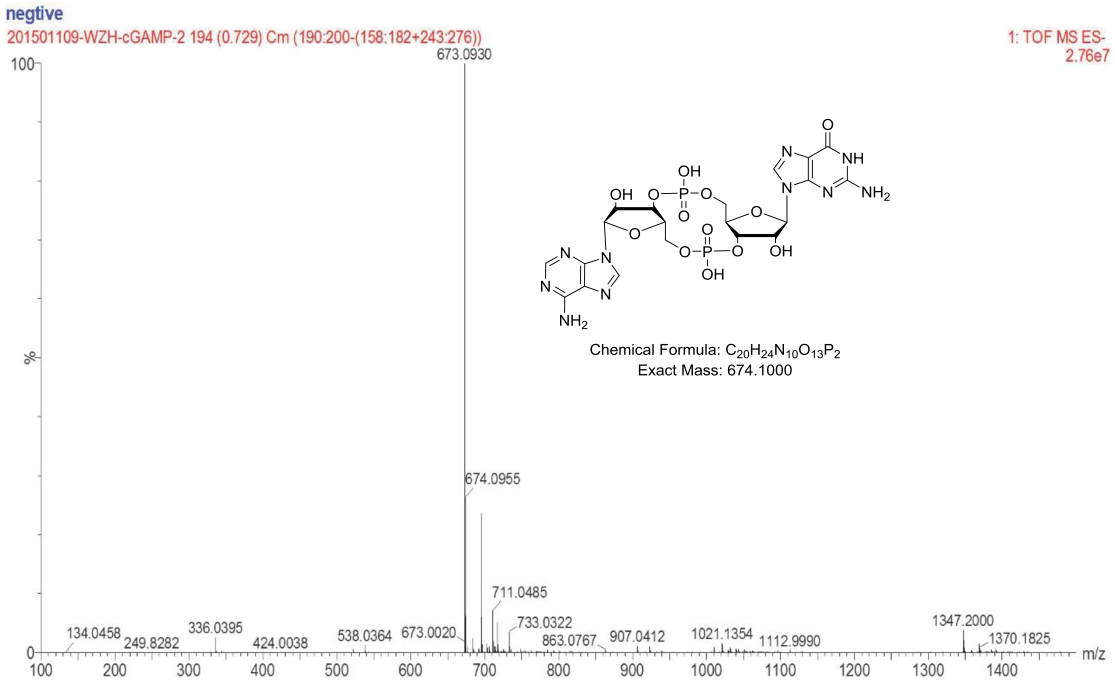

Figure S74. HRMS spectrum of **6e**
